# Biotinylated Diaryl Isoxazole Derivatives with Enhanced Janus Kinase 1 Inhibitory, Antiproliferative, and Apoptotic Activities in Cancer Cells

**DOI:** 10.1021/acsomega.5c08996

**Published:** 2026-06-17

**Authors:** Melike Ergüven, Hazal Beril Çatalak Yılmaz, Deniz Lengerli, Rana Acar, Servin Bagheralmoosavi, Yağmur Öykü Carus Şahin, Mehmet Gürçay, Burcu Çalışkan, Sreeparna Banerjee, Gurkan Yesiloz, Ozlen Konu, Erden Banoglu, Onur Cizmecioglu

**Affiliations:** † Department of Molecular Biology and Genetics, 52948 Bilkent University, Ankara 06800, Türkiye; ‡ Department of Biological Sciences, 52984 Orta Dogu Teknik Universitesi, Ankara 06800, Türkiye; § Department of Pharmaceutical Chemistry, Faculty of Pharmacy, 37511 Gazi University, Ankara 06560, Türkiye; ∥ National Nanotechnology Research Center (UNAM) Bilkent University, Ankara 06800, Türkiye

## Abstract

Isoxazole derivatives have emerged as promising scaffolds in anticancer drug discovery, yet their therapeutic potential remains to be fully harnessed. Here, we report on the synthesis and biological evaluation of two novel biotin-conjugated isoxazole derivatives (**C160** and **C161**) related to the parent compound **EB38**. Across a panel of liver, prostate, and breast cancer cell lines, both derivatives exhibited significantly enhanced antiproliferative and pro-apoptotic activities compared to **EB38**, with consistently lower IC_50_ values in 2D cultures and superior efficacy in 3D spheroid models. Mechanistic studies revealed that **C160** and **C161** robustly inhibit phosphorylation of JAK1 and STAT1, thereby suppressing JAK/STAT signaling and inducing apoptotic marker cleavage (PARP, Caspase-3, Caspase-7). Direct target engagement was further validated by CETSA and DARTS assays, which demonstrated thermal and proteolytic stabilization of endogenous JAK1 in the presence of **C160**. Molecular docking and HADDOCK simulations supported JAK1 as a direct cellular target, with binding affinities comparable to the clinically approved inhibitor Ruxolitinib. Importantly, zebrafish embryotoxicity assays confirmed that these derivatives do not elicit general cytotoxicity, even at doses substantially exceeding those effective *in vitro*. Collectively, our findings establish biotinylated isoxazole derivatives as potent JAK1-targeting anticancer agents with strong antiproliferative and pro-apoptotic activity, supporting their further development as promising candidates for targeted cancer therapy.

## Graphical abstract

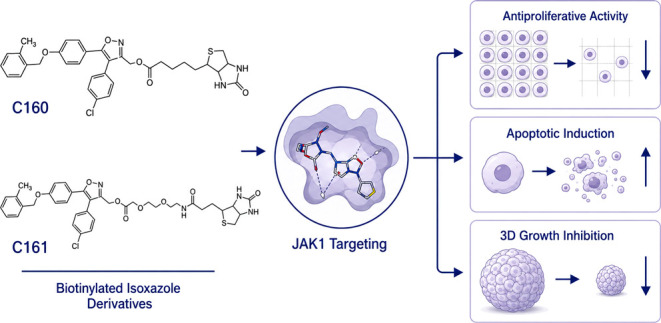

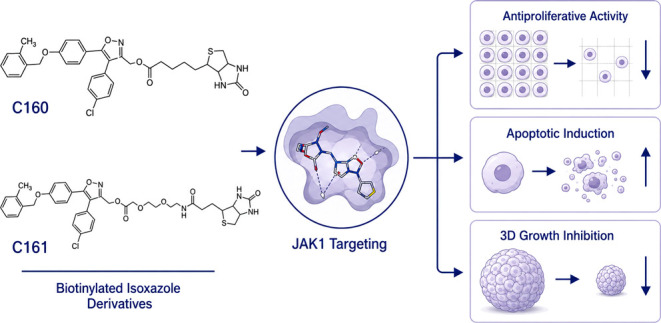

## Introduction

Cancer remains one of the leading causes of mortality worldwide, ranking among the top three causes of premature death from noncommunicable diseases.^1^ Despite significant advances in therapeutic strategies including surgery, chemotherapy, radiotherapy, targeted therapy, and immunotherapy, cancer continues to pose a substantial global health burden.^2^ Projections indicate that between 2022 and 2050, the number of new cancer cases will rise from 20 million to 35.3 million (International Agency for Research on Cancer, n.d.), while cancer-related deaths are expected to increase from 9.74 million to 18.5 million (International Agency for Research on Cancer, n.d.). Although current treatments have contributed to declining mortality trends, durable remissions and cures remain elusive due to challenges such as relapse, inherent or acquired drug resistance, and systemic toxicities that often limit treatment adherence.^3^

These limitations underscore the urgent need for novel anticancer agents that are both highly effective and selectively cytotoxic to cancer cells, while minimizing adverse effects. In this context, heterocyclic compounds have emerged as promising scaffolds in anticancer drug discovery. Characterized by ring structures containing at least one heteroatom such as nitrogen, oxygen, or sulfur,^4^ heterocycles are prevalent in biologically active molecules, including nucleic acid bases, amino acids, vitamins, and enzyme cofactors.^5^ Their structural versatility^6^ and ability to participate in a wide range of intermolecular interactions, such as hydrogen bonding, π–π stacking, metal coordination, and hydrophobic contacts make them ideal candidates for engaging diverse molecular targets.^7^

Among heterocyclic structures, isoxazoles, five-membered rings containing adjacent nitrogen and oxygen atoms, have attracted particular interest in cancer therapy.^8^ Isoxazoles are relatively easy to synthesize and chemically modifiable, allowing for a broad range of derivatives with diverse biological activities.^9^ Several isoxazole-based compounds have shown preclinical and clinical promise.^10^ For instance, the HSP90 inhibitor Luminespib (**NVP-AUY922**) has reached phase II clinical trials,^11^ while other isoxazole-containing agents such as **PNZ5** ^12^ and **XN05** ^13^ are in development for gastric and liver cancers (Figure 1). Additionally, FDA-approved anticancer drugs such as the VEGF inhibitor Tivozanib^14^ and the FLT3 inhibitor Quizartinib^15^ also incorporate isoxazole moieties, further highlighting their clinical relevance. We have also recently reported the anticancer potential of two diaryl heterocyclic compounds containing an isoxazole core: compound **1** (**EB38**) and its regioisomer (compound **2**), in which the positions of the nitrogen and oxygen atoms in **EB38** are interchanged (Figure 1). Both compounds exhibited notable antiproliferative activity against a panel of liver and breast cancer cell lines, with IC_50_ values ranging from 1.3 to 19 μM.^16^

**Figure 1:**
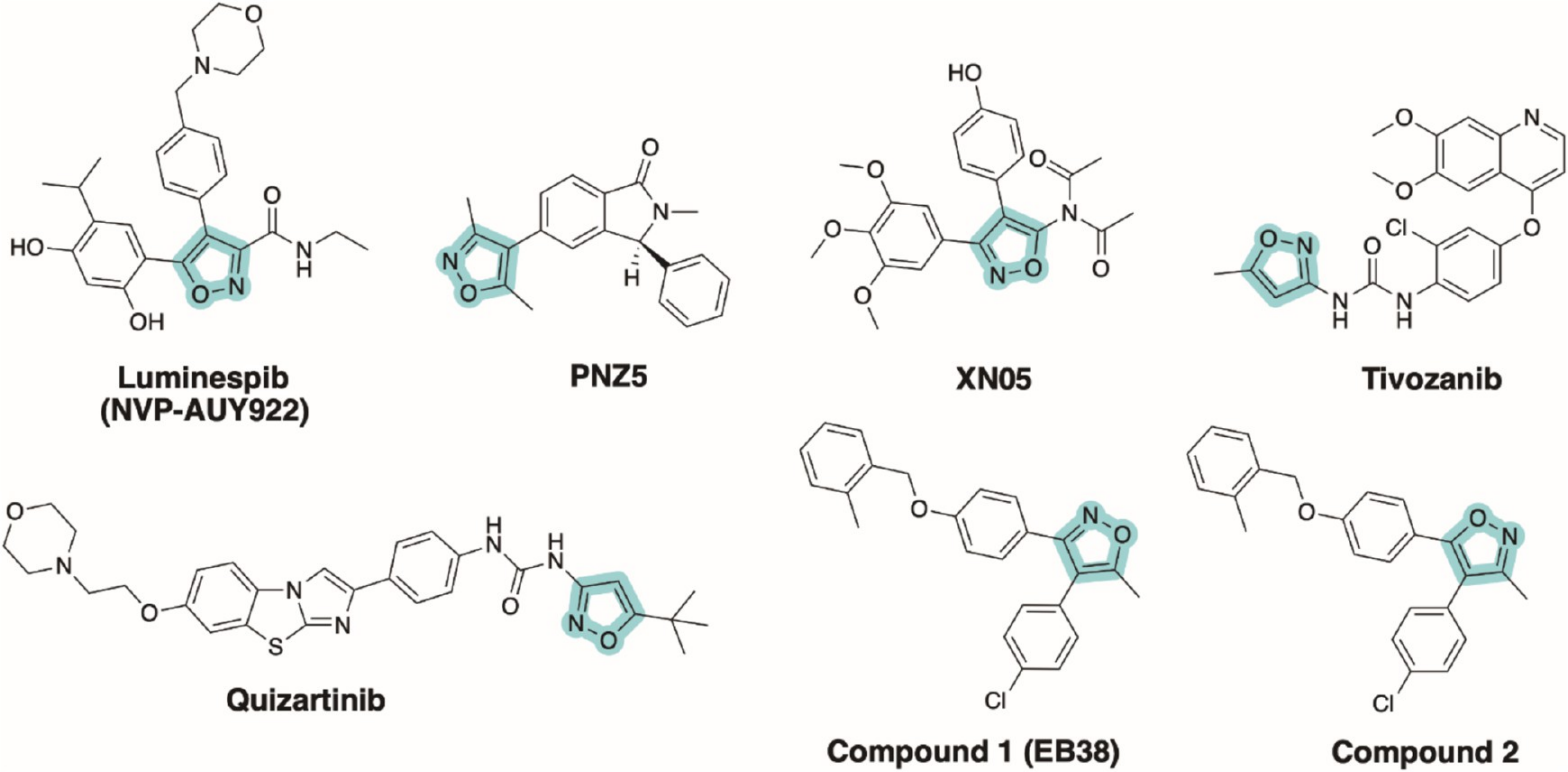
Examples of clinical drugs and experimental compounds with anticancer properties having an isoxazole group.

In addition to optimizing pharmacophores, enhancing drug delivery to tumor cells remains a key objective in cancer therapy. One promising strategy involves the conjugation of therapeutic agents to biotin, a vitamin that is often taken up at higher rates by cancer cells due to their increased metabolic activity.^17^ Studies have shown that many cancer cell lines overexpress biotin transporters, particularly sodium-dependent multivitamin transporters (SMVTs), thereby facilitating enhanced uptake of biotin-conjugated molecules. This approach has the potential to increase the selective accumulation of cytotoxic agents in cancer cells while sparing normal tissues, reducing the need for high systemic drug doses.

Despite the utility of this targeting strategy, the mechanisms underlying biotin-mediated cellular uptake remain incompletely understood. While the term “biotin receptor” is frequently used in the literature, no specific biotin receptors have been conclusively identified.^18^ Instead, SMVTs and, to a lesser extent, monocarboxylate transporters (MCTs) have been implicated in biotin uptake.^19^ Moreover, recent studies suggest that additional, transporter-independent mechanisms may contribute to cellular internalization. For example, it was reported that biotinylation enhanced intracellular accumulation of a fluorescent probe (Atto565), but this accumulation was not inhibited by free biotin, implying a noncompetitive uptake route.^20^ Similarly, it was demonstrated that the adhesion of cancer cells (HeLa and MCF7) to biotinylated surfaces was enhanced by excess free biotin but not by folic acid, another B vitamin, again suggesting alternative mechanisms.^21^ In line with these findings, recent work has challenged the assumption that biotin-conjugated drugs are internalized via the Sodium dependent multivitamin transporter (SMVT): in cell culture, uptake of biotin-conjugates was not competed by excess free biotin, suggesting that their internalization occurs through a SMVT-independent mechanism.^18^ Moreover, using biotin-furnished PAMAM dendrimers, uptake into HEK293 cells did not correlate with expression levels of the canonical biotin transporter SMVT; this suggests that internalization of biotin-conjugated nanoparticles often occurs via SMVT-independent mechanisms (e.g., alternative transporters or endocytosis) rather than via SMVT-mediated transport.^22^

These findings point to the need for a clearer mechanistic understanding of biotin-mediated delivery, as well as careful use of terminology in the field. Further investigation is required to define the contribution of canonical transporters versus nonclassical uptake pathways in mediating the intracellular delivery of biotin-conjugated agents.

Building on our earlier work synthesizing a library of vicinal diaryl-substituted heterocycles, including diaryl isoxazole derivatives (Compounds **1** and **2**, Figure 1), and demonstrating their potent anticancer activity,^16^ we now report the synthesis of two biotin-conjugated isoxazole analogs. This effort was driven by two goals: to assess whether biotinylation could enhance antitumor efficacy, and to elucidate the mechanistic basis of their action. Our findings reveal that the biotin-conjugated isoxazoles exhibit superior antiproliferative and pro-apoptotic activity in both 2D and 3D cancer models than the unbiotinylated **EB38**, which served as the reference compound in this study due to its greater antiproliferative potency over compound **2**. Moreover, integrative computational docking and biochemical validation identified JAK1 as a novel molecular target of **EB38** and two biotin conjugates, implicating the JAK/STAT signaling pathway as a key driver of their anticancer activity.

## Methods

### Chemistry

Starting chemicals, reagents, and solvents were purchased from local suppliers. Nuclear magnetic resonance (NMR) spectra (^1^H and ^13^C) were recorded in DMSO-*d*_6_ using a Bruker Avance Neo 500 MHz spectrometer or Varian Mercury 400 MHz spectrometer, with tetramethylsilane as the internal standard. Chemical shifts are reported in δ (ppm) and coupling constants in Hertz (Hz). High-resolution mass spectra (HRMS) were obtained on a Waters LCT Premier XE Mass Spectrometer in ESI­(+) mode, connected to an ultraperformance liquid chromatography (UPLC) system (Waters Corporation, Milford, MA, USA). The purity of all final compounds exceeded 95%, as determined by UPLC with UV detection, using a water/acetonitrile solvent gradient (0.1% formic acid, 20% → 80%) on an Aquity BEH C18 column (2.1 × 50 mm, 1.7 μm). Reactions were monitored by analytical thin-layer chromatography on precoated silica gel plates. Melting points were measured with a Stuart SMP50 automatic melting point apparatus (Schorpp Geraetetechnik, Germany) and were uncorrected. Chromatography was conducted using a Combiflash Rf Automatic Flash Chromatography System or a Reveleris Purification System (Buchi, New Castle, DE, USA) with RediSep columns (Teledyne-Isco, Lincoln, NE, USA) employing ethyl acetate/hexane or methanol/dichlorometane gradients. Microwave reactions were performed in Biotage sealed microwave vials using the Biotage Initiator+ microwave system.

#### 4-(4-Chlorophenyl)-5-methyl-3-(4-((2-methylbenzyl)­oxy)-phenyl)­isoxazole (**1**, **EB38**)

It was prepared as described previously.^16^ CAS: 2758520–84–0. Yield 35.0%; mp 117.8–118.5 °C. HRMS (*m*/*z*) [M + H]^+^ calcd for C_24_H_21_NO_2_Cl: 390.1261, found: 390.1243.

#### Ethyl 4-(4-Hydroxyphenyl)-2,4-dioxobutanoate (**4**)

Sodium ethoxide solution was freshly prepared by slowly adding small pieces of metallic sodium (58.8 mmol, 4.0 equiv) to 40 mL anhydrous ethanol under a nitrogen atmosphere with continuous stirring, while cooling the reaction mixture in an ice bath until the evolution of hydrogen gas ceased. 1-(4-hydroxyphenyl)­ethan-1-one (**3**) (14.7 mmol, 1.0 equiv) and diethyl oxalate (44.1 mmol, 3.0 equiv) were then added, and the mixture was stirred under nitrogen at room temperature for 5 h. After completion, slowly adding water to the reaction mixture, acidified with an aqueous HCl solution, and the resulting precipitate was collected by vacuum filtration, dried and used without further purification. CAS: 39974–01–1. Yield 94.0%; mp 145.7–147.8 °C. HRMS (*m*/*z*) [M + H]^+^ calcd for C_12_H_13_O_5_: 237.0763, found: 237.0752. ^**1**^ **H NMR (500 MHz**, **DMSO-** ***d***_***6***_ **):** δ 1.31 (3H, t, *J* = 7.1 Hz), 4.30 (2H, q, *J* = 7.1 Hz), 6.92 (2H, d, *J* = 8.8 Hz), 7.03 (1H, s), 7.98 (2H, d, *J* = 8.8 Hz), 10.70 (1H, s). HRMS (*m*/*z*) [M + H]^+^ calcd for C_12_H_13_O_5_: 237.0763, found: 237.0752. UPLC purity: 92.76%.

#### Ethyl 5-(4-Hydroxyphenyl)­isoxazole-3-carboxylate (**5**)

Compound **4** (1.0 equiv) and NH_2_OH•HCl (1.25 equiv) were refluxed in ethanol for 4 h. After completion, the reaction mixture was allowed to cool, and the resulting precipitate was collected by vacuum filtration and dried. The product was used directly without further purification. CAS: 1352896–92–4. Yield 93.0%; mp 181.5–183.0 °C. HRMS (*m*/*z*) [M + H]^+^ calcd for C_12_H_12_NO_4_: 234.0766, found: 234.0772. ^**1**^ **H NMR (500 MHz**, **DMSO-** ***d***_***6***_ **):** δ 1.33 (3H, t, *J* = 7.1 Hz), 4.38 (2H, q, *J* = 7.1 Hz), 6.91 (2H, d, *J* = 8.8 Hz), 7.21 (1H, s), 7.77 (2H, d, *J* = 8.8 Hz), 10.18 (1H, s). HRMS (*m*/*z*) [M + H]^+^ calcd for C_12_H_12_NO_4_: 234.0766, found: 234.0772. UPLC purity: 98.64%.

#### Ethyl 5-(4-((2-Methylbenzyl)­oxy)­phenyl)­isoxazole-3-carboxylate (**6**)

Compound **5** (1.0 equiv), 2-methylbenzyl bromide (1.3 equiv), and K_2_CO_3_ (1.7 equiv) were placed in a vial, followed by the addition of acetonitrile. The reaction was heated under microwave irradiation at 120 °C for 20 min. After cooling to room temperature, the mixture was poured into water and extracted with ethyl acetate. The organic layer was then dried, filtered, and evaporated to obtain the crude product, which was then washed with hexane. CAS: 1834560–37–0. Yield 92.0%; mp 114.3–115.2 °C. HRMS (*m*/*z*) [M + H]^+^ calcd for C_20_H_20_NO_4_: 338.1392, found: 338.1375. ^**1**^ **H NMR (500 MHz**, **DMSO-** ***d***_***6***_ **):** δ 1.34 (3H, t, *J* = 7.1 Hz), 2.33 (3H, s), 4.39 (2H, q, *J* = 7.1 Hz), 5.18 (2H, s), 7.21 (2H, d, *J* = 8.9 Hz), 7.22–7.28 (3H, m), 7.35 (1H, s), 7.43 (1H, d, *J* = 7.3 Hz), 7.91 (2H, d, *J* = 8.9 Hz). HRMS (*m*/*z*) [M + H]^+^ calcd for C_20_H_20_NO_4_: 338.1392, found: 338.1375. UPLC purity: 99.16%.

#### Ethyl 4-Bromo-5-(4-((2-methylbenzyl)­oxy)­phenyl)­isoxazole-3-carboxylate (**7**)

Compound **6** (1.0 equiv) dissolved in acetonitrile, then *N*-bromosuccinimide (NBS) (1.5 equiv) and ceric ammonium nitrate (CAN) (0.45 equiv) was added and stirred at room temperature overnight. The reaction mixture was taken into ice–water and extracted with ethyl acetate; the organic layer was dried with anhydrous Na_2_SO_4_ and evaporated. The crude product was purified by automated flash column chromatography (0% → 60% EtOAc in Hexane). CAS: 1834560–58–5. Yield 75.0%; mp 84.2–85.9 °C. HRMS (*m*/*z*) [M + H]^+^ calcd for C_20_H_19_BrNO_4_: 416.0497, found: 416.0478. ^**1**^ **H NMR (500 MHz**, **DMSO-** ***d***_***6***_ **):** δ 1.35 (3H, t, *J* = 7.1 Hz), 2.34 (3H, s), 4.42 (2H, q, *J* = 7.1 Hz), 5.20 (2H, s), 7.20–7.26 (3H, m), 7.28 (2H, d, *J* = 9.0 Hz), 7.43 (1H, d, *J* = 7.4 Hz), 7.98 (2H, d, *J* = 9.0 Hz). HRMS (*m*/*z*) [M + H]^+^ calcd for C_20_H_19_BrNO_4_: 416.0497, found: 416.0478. UPLC purity: 99.14%.

#### Ethyl 4-(4-Chlorophenyl)-5-(4-((2-methylbenzyl)­oxy)­phenyl)­isoxazole-3-carboxylate (**8**)

Compound 7 (1.0 equiv) and Na_2_CO_3_ (6.0 equiv) were dissolved in a Dioxane:H_2_O (5:1, v/v) and degassed with nitrogen for 15 min, followed by the addition of 4-chlorophenylboronic acid (1.3 equiv) and PdCl_2_(PPh_3_)_2_ (0.05 equiv). The reaction was stirred at 80 °C for 6 h. At the end of the reaction period, the mixture was poured into water and extracted with ethyl acetate. The organic layer was dried over anhydrous Na_2_SO_4_ and evaporated. The resulting crude product was purified by automated flash column chromatography (0% → 30% EtOAc in Hexane) and recrystallized from methanol. CAS: 1834560–76–7. Yield 62.0%; mp 116.6–117.8 °C. HRMS (*m*/*z*) [M + H]^+^ calcd for C_26_H_23_ClNO_4_: 448.1316, found: 448.1294. ^**1**^ **H NMR (500 MHz**, **DMSO-** ***d***_***6***_ **):** δ 1.15 (3H, t, *J* = 7.1 Hz), 2.30 (3H, s), 4.24 (2H, q, *J* = 7.1 Hz), 5.11 (2H, s), 7.12 (2H, d, *J* = 9.0 Hz), 7.19–7.25 (3H, m), 7.39 (1H, d, *J* = 7.5 Hz), 7.40–7.43 (4H, m), 7.53 (2H, d, *J* = 8.6 Hz). HRMS (*m*/*z*) [M + H]^+^ calcd for C_26_H_23_ClNO_4_: 448.1316, found: 448.1294. UPLC purity: 99.26%.

#### (4-(4-Chlorophenyl)-5-(4-((2-methylbenzyl)­oxy)­phenyl)­isoxazol-3-yl)­methanol (**9**)

Compound **8** (1.0 equiv) was dissolved in a THF:MeOH (4:1, v/v) with NaBH_4_ (3.0 equiv) and stirred at 0 °C for 5 h. After completion, the reaction mixture was added to water, acidified with an aqueous HCl solution, and the resulting precipitate was filtered. The crude product was purified by automated flash column chromatography (0% → 60% EtOAc in Hexane). Yield 84.0%; mp 160.4–162.4 °C. ^**1**^ **H NMR (400 MHz**, **DMSO-** ***d***_***6***_ **):** δ 2.31 (3H, s), 4.44 (2H, d, *J* = 5.6 Hz), 5.11 (2H, s), 5.45 (1H, t, *J* = 5.6 Hz), 7.11 (2H, d, *J* = 8.4 Hz), 7.18–7.27 (3H, m), 7.39–7.46 (5H, m), 7.52 (2H, d, *J* = 8.4 Hz). HRMS (*m*/*z*) [M + H]^+^ calcd for C_24_H_21_ClNO_3_: 406.1210, found: 406.1194. UPLC purity: 95.13%.

#### (4-(4-Chlorophenyl)-5-(4-((2-methylbenzyl)­oxy)­phenyl)­isoxazol-3-yl)­methyl.5-(2oxohexahydro-1H-thieno­[3,4-*d*]­imidazol-4-yl)­pentanoate (**10**, **C160**)

Compound **9** (1.0 equiv) was dissolved in DMF, followed by the addition of biotin (5.9 equiv), 1-ethyl-3-(3-(dimethylamino)­propyl)­carbodiimide (EDC) (8.9 equiv) and 4-dimethylaminopyridine (DMAP) (2.2 equiv). The reaction mixture was stirred at room temperature overnight. Upon completion, it was poured into water, neutralized with NaHCO_3_, and extracted with ethyl acetate. The organic layer was dried over anhydrous Na_2_SO_4_, and the crude product was purified by preparative column chromatography (0% → 20% MeOH in DCM). Yield 78.0%; mp 81.8–83.3 °C. ^**1**^ **H NMR (500 MHz**, **DMSO-** ***d***_***6***_ **):** δ 1.22–1.27 (2H, m), 1.38–1.41 (3H, m), 1.42–1.44 (1H, m), 2.18 (2H, t, *J* = 7.4 Hz, H2), 2.30 (3H, s, H35), 2.58 (1H, d, *J* = 12.4 Hz), 2.82 (1H, dd, *J* = 12.4, 5.1 Hz), 3.05–3.07 (1H, m), 4.11–4.14 (1H, m), 4.30 (1H, dd, *J* = 7.8, 5.1 Hz), 5.11 (2H, s, H30), 5.12 (2H, s, H17), 6.38 (1H, s, H9), 6.44 (1H, s, H12), 7.11 (2H, d, *J* = 9.0 Hz, H25), 7.18–7.27 (3H, m), 7.38–7.44 (5H, m, H24, H38), 7.55 (2H, d, *J* = 8.5 Hz, H37). ^**13**^ **C NMR HSQC** **& HMBC (125 MHz**, **DMSO-** ***d***_***6***_ **):** δ 18.9, 24.7, 28.3, 28.4, 33.3, 55.7, 56.5, 59.6, 61.4, 68.5, 113.6, 115.8, 119.6 (C23- H25,25^ı^), 126.2, 128.6, 128.7, 128.8, 129.1, 129.6, 130.6, 132.0, 133.7, 134.8, 137.1, 159.4 (C20–H17), 160.5 (C26–H30), 163.1 (C8- H9, H12), 165.5 (C22–H24,24^ı^), 172.5 (C1, H2). HRMS (*m*/*z*) [M + H]^+^ calcd for C_34_H_35_ClN_3_O_5_S: 632.1986, found: 632.1995. UPLC purity: 97.42%.

#### (4-(4-Chlorophenyl)-5-(4-((2-methylbenzyl)­oxy)­phenyl)­isoxazol-3-yl)­methyl.2-(2-(2-(5­(2-oxohexahydro-1H-thieno­[3,4-*d*]­imidazol-4-yl)­pentanamido)­ethoxy)­ethoxy) acetate (**11**, **C161**)

It was synthesized from compound **9** using pegylated biotin under the same conditions employed for the preparation of **C160**. The resulting crude product was purified by flash column chromatography (0% → 20% MeOH in DCM). Yield 63.0%; mp 99.3–100.7 °C. ^**1**^ **H NMR (500 MHz**, **DMSO-** ***d***_***6***_ **):** δ 1.28–1.31 (2H, m), 1.46–1.48 (3H, m), 1.49–1.51 (1H, m), 2.06 (2H, t, *J* = 7.4 Hz, H12), 2.31 (3H, s, H47), 2.58 (1H, d, *J* = 12.4 Hz), 2.81 (1H, dd, *J* = 12.4, 5.1 Hz), 3.08–3.09 (1H, m), 3.19 (2H, q, *J* = 5.8 Hz), 3.39 (2H, t, *J* = 5.9 Hz, H9), 3.50 (4H, m), 4.04 (2H, s, H2), 4.12–4.13 (1H, m), 4.28–4.30 (1H, m), 5.11 (2H, s, H40), 5.19 (2H, s, H27), 6.35 (1H, s, H19), 6.41 (1H, s, H20), 7.12 (2H, d, *J* = 8.9 Hz, H35), 7.18–7.27 (3H, m), 7.38–7.44 (5H, m, H34, H48), 7.55 (2H, d, *J* = 8.5 Hz, H47), 7.80 (1H, t, *J* = 5.8 Hz, H17). ^**13**^ **C NMR HSQC** **& HMBC (125 MHz**, **DMSO-** ***d***_***6***_ **):** δ 18.9, 25.7, 28.5, 28.6, 35.5 (C12), 38.8 (C10–H17), 55.8, 56.6 (C27), 59.6, 61.5, 67.8 (C2), 68.6 (C40), 69.6, 69.8­(C9), 70.4, 113.7, 115.8 (C35, 35^ı^-H34,34^ı^), 119.6 (C33–H35,35^ı^), 126.2, 128.5, 128.7, 128.8, 129.0, 129.6, 130.6, 132.1, 133.7, 134.8, 137.1, 159.1 (C30–H27), 160.5 (C36–H38), 163.1 (C18–H19, H22), 165.6 (C32–H34), 169.9 (C1–H2, H27), 172.5 (C11–H12, H17). HRMS (*m*/*z*) [M + H]^+^ calcd for C_40_H_45_ClN_4_O_8_S: 777.2725, found: 777.2747. UPLC purity: 96.50%.

### Cell Culture

All cell lines were routinely cultured at 37 °C in a humidified environment with 5% CO_2_. Mahlavu and MDA-MB-231 cells were cultured in high glucose DMEM (Gibco) supplemented with 10% fetal bovine serum (FBS) (Capricorn), 100 units/mL penicillin-100 μg/mL streptomycin (Gibco), and 1× MEM non-essential amino acids (VivaCell). SNU475 cells were cultured in high glucose DMEM (Gibco) supplemented with 10% FBS (Capricorn), 100 units/mL penicillin-100 μg/mL streptomycin (Gibco), 2 mM l-glutamine (Cytiva), and 1× MEM non-essential amino acids (VivaCell). C4–2, PC3, and DU145 cells were cultured in RPMI-1640 (Gibco) supplemented with 10% FBS (Capricorn), and 100 units/mL penicillin-100 μg/mL streptomycin (Gibco). MCF10A cells were cultured in DMEM/F12 (Gibco) supplemented with 4% FBS (Capricorn), 100 units/mL penicillin-100 μg/mL streptomycin (Gibco), 1× l-glutamine (Cytiva), 0.01 ng/mL EGF (GenScript), 25 ng/mL hydrocortisone, 0.01 mg/mL insulin (Capricorn). T47D cells were cultured in high glucose DMEM (Gibco) supplemented with 8% FBS (Capricorn), 100 units/mL penicillin-100 μg/mL streptomycin (Gibco), 2 mM l-glutamine (Cytiva), 1× MEM non-essential amino acids (VivaCell), and 4 μg/mL insulin (Capricorn). Cells were routinely checked for mycoplasma contamination.

### Sulforhodamine B Assay

MCF10A, Mahlavu, SNU475, MDA-MB-231, PC3, and DU145 cells were seeded into 96-well plate at a density of 2000 cells per well. C4–2 cells were seeded into 96-well plate at a density of 10,000 cells per well. T47D cells were seeded into 96 well plate at a density of 3000 cells per well. Cells were treated with compounds at concentrations ranging from 0.3 to 10 μM for 3 days after they have grown for 2 days in 96 well plates. DMSO (Serva) was utilized as negative control. Following the end of treatment with compounds, cells were fixed with 10% trichloroacetic acid (TCA) (w/v) (Sigma-Aldrich) for 1 h at 4 °C. 96-well plates were washed with dH_2_O for 5 times. Cells were incubated with 0.4% SRB (w/v) (Sigma-Aldrich) in acetic acid for 30 min at room temperature in the dark. Cells were washed with 1% acetic acid until removing all unbound stain and left for air-dry. Ten mM Tris-base was added to extract bound SRB dye from cells. Absorbances were read at 564 nm using Biotek Synergy HT microplate reader. Experiments were performed in 3 biological replicates with 3 technical replicates.

### Immunoblotting

Sub confluent Mahlavu and DU145 cells were treated with compounds for either 24 h and/or 48 h (1 μM, 5 μM, and DMSO (Serva) as negative control). After treatment ends, cells were rinsed with 1× PBS and scraped within 1× ice-cold PBS on ice. Cell pellets were obtained after centrifugation at 1500*g* for 8 min at 4 °C. Cell pellets were incubated with lysis buffer composed of RIPA (Intron Biotechnology) (150 mM NaCl, 50 mM Tris-HCl pH 7.5, 0.5% sodium deoxycholate, 1% Triton-X, 0.1% SDS, 2 mM EDTA), 1 mM DTT (Thermo Fisher), 1 mM sodium orthovanadate (Na3VO4) (Sigma-Aldrich), 1× phosphatase inhibitor (Serva), 1× protease inhibitor (GlpBio) for 30 min on ice. Lysates were collected by centrifuging at 13000 rpm for 15 min at 4 °C. Protein concentrations in the lysates were determined with Bicinchoninic Acid (BCA) protein assay (Thermo Fisher). 10–20 μg of proteins were loaded into SDS-PAGE gel for protein separation. Proteins were transferred to nitrocellulose membrane (GVS) through wet transfer method. Membranes were blocked with 5% Bovine Serum Albumine (w/v) (Capricorn) in TBS-T for 1 h at room temperature. Following blocking, membranes were incubated with primary antibodies overnight at 4 °C with shaking. Catalog numbers of primary antibodies used are given in the table. Next day, membranes were washed with TBS-T 3 times, incubated with secondary antibodies for 1 h at room temperature, and washed with TBS-T 3 times. Proteins were visualized with ECL (Advansta) using Amersham Imager 600. The following antibodies were used in this study: anti-JAK1 (Cell Signaling Technologies, Cat. No. 50996), antiphospho-JAK1 (Tyr1022) (St John’s Laboratory, Cat. No. STJ90314), anti-STAT1 (Cell Signaling Technologies, Cat. No. 9172), and antiphospho-STAT1 (Tyr701) (Cell Signaling Technologies, Cat. No. 9167). For the PI3K/AKT pathway, antibodies against AKT1 (BioLegend, Cat. No. 680302) and phospho-AKT (Ser473) (Cell Signaling Technologies, Cat. No. 9271) were employed. As loading controls, β-actin (Sigma/Merck, Cat. No. 66009) and GAPDH (Santa Cruz, Cat. No. SC-47724) antibodies were used. To assess apoptosis, antibodies against cleaved Caspase-3 (Cell Signaling Technologies, Cat. No. 9661), cleaved Caspase-7 (Cell Signaling Technologies, Cat. No. 8438), and cleaved PARP (Cell Signaling Technologies, Cat. No. 5625) were utilized. Secondary antibodies included HRP-conjugated goat antimouse IgG (H + L) (Advansta, Cat. No. R-05071) and goat antirabbit IgG (H + L) (Advansta, Cat. No. R-05072).

### Drug Affinity Responsive Target Stability (DARTS)

Mahlavu cells were grown to 90% confluency. Cells were washed and scraped with ice cold PBS. Cell pellets were lysed with NP-40 lysis buffer (0.5% v/v NP-40, 50 mM Tris-HCl pH 7.5, 150 mM NaCl, 5 mM EDTA) supplemented with protease inhibitor cocktail (GlpBio), phosphatase inhibitor (Serva), 1 mM DTT (Thermo Fisher), 1 mM sodium orthovanadate (Sigma-Aldrich) for 30 min on ice. Lysates were quantified with Bicinchoninic Acid (BCA) protein assay (Thermo Fisher). 99 μL of lysates with concentration of 2.5 μg/μL were split into three as vehicle control and treatment groups. Lysates were incubated with 1 μL of DMSO, **AZD1480**, and **C160** in final concentration of 10 μM for 30 min on rotator. 100 μL were split into 20 μL samples and treated with different dilutions of Pronase E (MedChemExpress). Different concentrations of Pronase E solutions were prepared in 1× TNC buffer. Samples were incubated with different dilutions of Pronase E (2 μL) for 20 min at room temperature. Activity of Pronase E was quenched with addition of 2 μL of 20× protease inhibitor and they were incubated for 10 min on ice. Each sample was mixed with 8 μL of 4× SDS-PAGE loading buffer and heated at 70 °C for 10 min. Samples were analyzed with Western blotting.

### Cellular Thermal Shift Assay

Mahlavu cells were grown to 90% confluency. Cells were washed and scraped with ice cold PBS. Cell pellets were lysed with RIPA lysis buffer (Intron Biotechnology) (150 mM NaCl, 50 mM Tris-HCl pH 7.5, 0.5% sodium deoxycholate, 1% Triton-X, 0.1% SDS, 2 mM EDTA) supplemented with protease inhibitor cocktail (GlpBio), phosphatase inhibitor (Serva), 1 mM DTT (Thermo Fisher), 1 mM sodium orthovanadate (Sigma-Aldrich) for 30 min on ice. Lysates were quantified with Bicinchoninic Acid (BCA) protein assay (Thermo Fisher). Lysates were divided into two groups as vehicle control and treatment group. Lysates were incubated with 5 μM of **C160** (or DMSO) for 1 h at room temperature. After incubation ends, samples were divided into PCR tubes and heated at indicated temperatures for 3 min. After heating, they were placed on ice. Supernatant were collected by centrifuging at 16.000 rcf for 20 min at +4 °C.

### Spheroid Formation and Compound Testing

A method outlined in previous studies^23^ was used to develop platforms for generating C4–2 spheroids. Briefly, a customized mold was utilized to form a platform containing 24 μm-sized wells. Each platform was designed to fit in one well of a 96-well plate. These platforms were sterilized via UV (ultraviolet) before cell seeding. Approximately, 400 cells per microwell were seeded in each platform. Three days after cell seeding, when the cells formed tight spheroids, the media with varying concentrations of the compounds (0.75, 1.5, 3, 5, and 10 μM) were added to the spheroids. Spheroids were treated with compounds for 3 days. Individual spheroid diameters were measured for growth analysis using Carl Zeiss Axio Observer 7 microscope.

Liquid overlay-based method was used to generate T47D spheroids. Surface of 96-well plate was covered with 50 μL of 1% agarose. After agarose was solidified, 3000 cells in 200 μL were seeded into each well. The cells were incubated for 5 days to allow for spheroid generation. Treatment with the indicated concentrations were initiated on day 5 of spheroid generation, which was designated as day 0. After spheroids were formed, their media were refreshed every 3 days with compound-containing media. Spheroid images were taken with Evos FL Auto imaging system and spheroid volumes were measured with AnasP Software.^24^

### p*K*_a_ Predictions

The p*K*_a_ values of the synthesized ligands and Ruxolitinib were predicted by using an online tool based on graph neural networks called MolGpKa.^25^ The p*K*_a_ values of Ruxolitinib were also obtained from DrugBank (https://go.drugbank.com/drugs/DB08877, retrieved on 29.11.2025).

### Computational Docking Stimulations

The SMILES formulas of molecules synthesized were used in Avogadro to generate their 3D structures.^26^ The three molecules were built by using their SMILES formulas on Avogadro as they were not crystallized before. To find out their most optimal conformations, the 3D structures generated by Build function on Avogadro were used in Optimize Geometry function. Following that, their best conformations were determined by Conformer Search function on Avogadro. Systematic rotor search option was utilized in Conformer Search function. The polar hydrogens on the ligands were added in AutoDock Tools.^27^ The protonated side groups were checked by using MolGpKa.^25^ The cocrystallized ligands with JAK1 models were removed from the kinase structure on PyMOL. (The PyMOL Molecular Graphics System, Version 3.0 Schrödinger, LLC). The docking was performed by using AutoDock Vina software.^28,29^ For that, configuration files for each ligand and protein couple were prepared on the following criteria on docking space, gridbox size and locations, and the total number of modes. The docking space was determined for each kinase model by using AutoDock Tools GridBox function. The size of the gridbox was set to 18 × 18 × 18 Å^3^ and the grid spacing to 1 Å to accommodate the full catalytic pocket of JAK1. The gridbox locations were determined as the catalytic site which was previously filled by the cocrystallized ligand and they were adjusted for each JAK1 kinase model. The total number of modes in the configuration files were set to 10. The ligands were set to be flexible and the kinase models were set to be rigid. Furthermore, the catalytic sites previously occupied by ligands were confirmed as the kinase domains. The PDBQT files were generated by using AutoDock Tools utility scripts. The configuration scripts and the PDBQT files were used to run on Vina. All of the cocrystallized ligands were used in self-docking studies after being subjected to the same conformer search process. The RMSD cutoff was set to 2 Å as it was accepted to be the correct bond structure.^28,29^ The PDBID list of crystal structures used for Vina is as follows: 6N7A, 6N77, 6N78, 6N79, 6N7B, 6N7C, 6N7D, 6RSB, 6RSC, 6RSD, 6RSE, 6BBU, 5E1E, 5HX8, 6DBN, 6TPE, 6W8L, 6SM8, 6SMB, 6GGH, 6ELR, 6RSH, 5WO4, 6HZU.^30–39^

In order to obtain statistical significance from the affinity values from Vina for the three newly synthesized ligands and Ruxolitinib, each docking simulation was performed for one hundred times by using an automated batch-processing script. The affinity values for each iteration were obtained from log files and used in generating bar plots on Python and GraphPad Prism 10. (GraphPad Prism version 10.0.0 for Windows, GraphPad Software, Boston, Massachusetts USA, www.graphpad.com) The statistical analyses of affinity values obtained for each ligand were performed on GraphPad Prism 10.

HADDOCK simulations were performed by using the default settings for protein and small molecule dockings.^40^ The active residues were determined by using PyMOL. (The PyMOL Molecular Graphics System, Version 3.0 Schrödinger, LLC.) For each protein model, the active residues were selected according to the already-docked ligand to the original protein model and checked whether the pocket is the kinase domain. For each ligand, the protein models that generated the lowest affinity values from Vina were selected to be run on HADDOCK. For **C160** and **C161**, the protein structure with the lowest affinity value was 6RSB (Structure 07). For **EB38**, 6N79 (Structure 04) generated the best affinity score. For both of the protein structures, Ruxolitinib counterparts were also docked for comparison.

The online tool PLIP was used to carve out the active sites for labeling fast and effectively.^41^ To compare different binding modalities of the ligands, an overall most representative protein structure was selected. The selection criteria included a −8.37 kcal/mol affinity value for each ligand as well as exhibiting the same order of affinity scores as the one hundred docking iterations.

### Statistical Analysis and IC_50_ Calculations

GraphPad Prism 10 was used for statistical analysis and calculation of IC_50_ values of the compounds. Concentration values were transformed using the formula *X* = log­(*X*) and normalized. Nonlinear regression (curve fit) model was used. Log (inhibitor) vs normalized response-variable slope equation was used to draw IC_50_ curves. Significant differences were determined based on ns > *p* 0.05; * *p* < 0.05; **p < 0.01; ***p < 0.001; ****p < 0.0001. Two-way ANOVA and one-way ANOVA (followed by multiple comparison tests) were used to compare experimental groups statistically.

### Lysis of Cells

Lysates of sub confluent cells treated with the three different compounds for 24 h [1 μM, 5 μM, and DMSO (Serva) as negative control] were obtained using a urea-based lysis protocol. C4–2 cells were washed 3 times with ice-cold 1× PBS containing 1 mM Na_3_VO_4_ (Sigma-Aldrich) and 1 mM NaF (Merck). Cells were scraped in lysis buffer composed of 8 M urea (Sigma-Aldrich) in 20 mM HEPES (pH 8.0), 1 mM Na_3_VO_4_ (Sigma-Aldrich), 1 mM NaF (Merck), 1 mM β-glycerol phosphate, 2.5 mM disodium pyrophosphate (Na_2_H_2_P_2_O_7_) (Sigma-Aldrich). Cells were sonicated at 40% amplitude for 15 s 4 times, including 25 s rest between the sonification steps. Protein lysates were obtained after centrifugation at 14000 rpm for 20 min at 4 °C.

### Plasmids and Generation of Stable Cell Line

The PH-BTK-GFP vector (Addgene, Plasmid #51463) was digested with *Eco*RI (NEB) and HincII (NEB) restriction enzymes and cloned into pBabe-Puromycin vector digested with *Eco*RI and HincII enzymes. HEK293 cells were transfected with pCMV-VSV-G (Addgene Cat#8454), gag/pol (Addgene Cat#14887) and the cloned vector with lipofectamine 3000 (Thermo Fisher) according to instructions of manufacturer. C4–2 cells were transduced with retroviral particles generated in HEK293 cells and underwent puromycin selection.

### Fluorescence Microscopy

PH-BTK-GFP-C4–2 cells were seeded at a density of 350.000 cells per well to 6 well plates with coverslips inserted. Next day, cells were treated with 5 μM **EB38**, 5 μM **C160**, 3 μM **GDC-0941** for 6 h. At the end of the treatment, the wells were washed with 1× PBS and fixed with 4% paraformaldehyde (PFA) (w/v) in 1× PBS for 15 min at room temperature. The wells were washed 3 times with 1× PBS. The coverslips with the adhered cells were pried up and washed with ddH2O and any excessive water was wicked away using tissue paper. The coverslips were mounted on a drop of mounting medium with DAPI and incubated overnight in the dark at room temperature. Next day, the coverslips were visualized under Zeiss AXIO Imager A1. Cells transfected with the PH-BTK- GFP construct exhibit diffused cytoplasmic GFP signals under basal conditions. Upon PI3K activation, the GFP tagged protein localizes to the cell membrane with expression of the GFP reporter in the cell periphery.

### Zebrafish Husbandry and Acute Aquatic Toxicity Assay

Adult wild-type zebrafish (AB strain) were maintained at 27 ± 1 °C in a circulating water system with 14 h light:10 h dark cycles. Embryos were collected at noon and incubated in a Petri dish with E3 media (4.91 mM NaCl, 0.17 mM KCl, 0.33 mM CaCl_2_.2H_2_O, 0.33 mM MgSO_4_.7H2O, and methylene blue) at 28 °C in an incubator until 2 days-postfertilization (dpf) with regular media renewal.

For the toxicity assay, healthy 2 dpf larvae were distributed to a 48 well plate at a density of six larvae per well (*n* = 3 wells with a total of 18 larvae per treatment group) after physical dechorionation. Larval groups were treated with varying concentrations of compounds (2, 5, 10 μM or 10, 30, 50 μM) in 500 μL E3 media per well for 72 h from 2 to 5 dpf with daily renewal of each treatment. As positive control treatments, **AZD1480** and sorafenib (**SFB**) at 2–10 μM were used. Survival rates were recorded, and post hoc power analysis on survival rate of **SFB** and its DMSO control groups was performed in R using pwr.2p2n.test function of pwr package (v1.3).^42^ At 5 dpf, larvae were anesthetized using 0.04% tricaine (MS-222), laterally positioned in 3% methylcellulose (w/v), and brightfield images of representative larvae (*n* = 5–6) were taken using Leica MZ10F stereomicroscope for morphometric assessments of standard length, eye size, swim bladder inflation rate, and edema formation by ImageJ.^43^

## Results

### Chemistry

Compound **1** (**EB38**), as the reference compound, was synthesized following our previously reported procedure.^16^ Compound **2** was chosen for the synthesis of biotinylated isoxazole derivatives due to its favorable synthetic accessibility and the ready availability of starting materials, as depicted in Figure 1. The diketone derivative **4** was first prepared by reacting 1-(4-hydroxyphenyl)­ethan-1-one (**3**) with diethyl oxalate in a sodium ethoxide solution. Compound **4** was then cyclized using hydroxylamine hydrochloride (NH_2_OH·HCl) to afford compound **5**.^44^ Subsequently, **5** underwent O-alkylation with 2-methylbenzyl bromide under microwave irradiation to yield compound **6**. Bromination of **6** using N-bromosuccinimide (NBS) and cerium ammonium nitrate (CAN) resulted in the synthesis of compound **7**, which was subjected to Suzuki coupling with 4-chlorophenylboronic acid to obtain compound **8**.^45^ Reduction of 8 with NaBH_4_ produced compound **9**. Finally, **9** was coupled with either biotin or pegylated biotin (which might improve solubility and flexibility) using 1-ethyl-3-(3-(dimethylamino)­propyl)­carbodiimide (EDC) and 4-dimethylaminopyridine (DMAP), resulting in the formation of compounds **10** (**C160**) and **11** (**C161**), respectively (Figure 2).^46^

**Figure 2:**
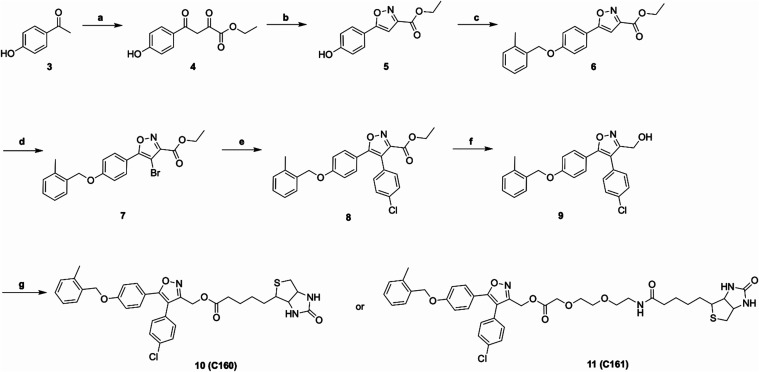
Reactions conditions and reagents for **C160** and **C161**: (a) Diethyl oxalate, NaOEt, EtOH rt, 5 h; (b) NH_2_OH•HCl, EtOH, reflux, 4 h; (c) 2-methylbenzyl bromide, K_2_CO_3_, MeCN, MWI, 120 °C, 20 min; (d) NBS, CAN, MeCN, rt, overnight; (e) Na_2_CO_3_, 4-chlorophenylboronic acid, PdCl_2_(PPh_3_)_2_, Dioxane:H_2_O, 80 °C, 6 h; (f) NaBH_4_, THF:MeOH, 0 °C, 5 h; (g) Biotin (for **C160**) or pegylated biotin (for **C161**), EDC, DMAP, DMF, rt, overnight.

### Cellular Biology

Given that biotin conjugation can enhance the bioavailability of chemical compounds, we aimed to assess the comparative potency of **EB38** and the biotinylated isoxazole derivatives (**C160** and **C161**) in cellular proliferation assays across various cancer cell lines. Although compound **2** represents the direct precursor of the biotin conjugates, **EB38** was selected as the reference compound because it is the more potent and biologically representative regioisomer of this scaffold class, exhibiting 2–3-fold stronger activity than compound **2** in Huh7, Mahlavu, and MCF-7 cells, as previously reported.^16^ Therefore, using this benchmark allowed us to determine whether biotin conjugation could enhance the activity of the most active member of the scaffold family. To this end, we performed sulforhodamine B (SRB) proliferation assays in prostate (PC3, DU145, C4–2), liver (Mahlavu, SNU475), and breast (T47D, MDA-MB-231) cancer cell lines, which collectively represent major solid tumor types. Across all tested cancer models, the biotin- and PEG-biotin-conjugated isoxazole derivatives (**C160** and **C161**, respectively) consistently exhibited greater antiproliferative activity compared to the **EB38** (Figure 3A–G). Importantly, this enhanced efficacy exceeds that of not only **EB38** but also the less active regioisomer, compound **2**, underscoring that the improvement cannot be attributed to intrinsic scaffold differences alone. In contrast, in quasi-normal MCF10A breast epithelial cells, the conjugates showed markedly reduced inhibitory effects (Figure 3H), consistent with a degree of cancer-selective activity.

**Figure 3:**
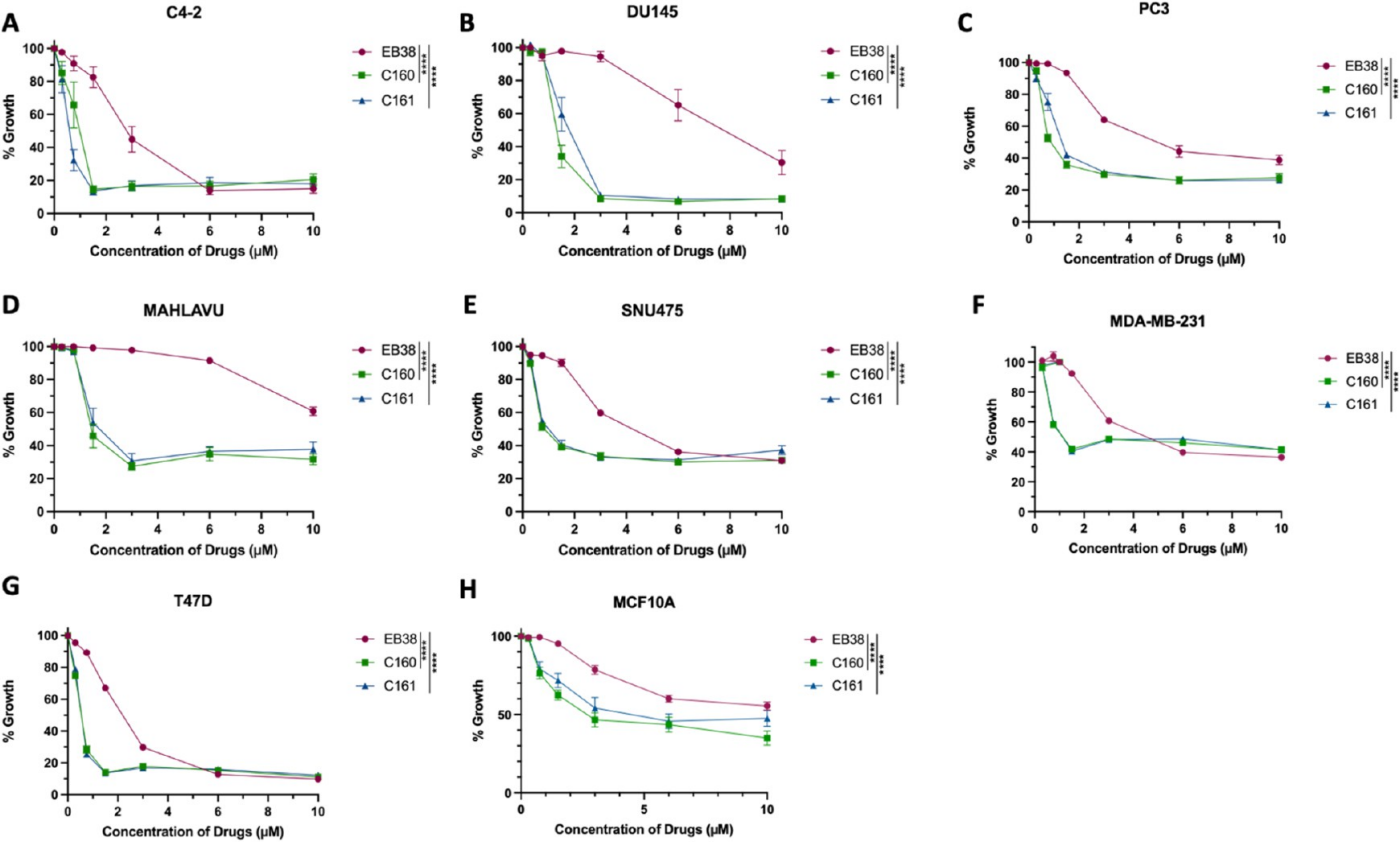
Biotinylated compounds exhibited broad-spectrum antiproliferative activity in multiple cancer cell models. Cellular proliferation was quantified using the Sulforhodamine B (SRB) assay on cancer cell lines treated with the compounds for 72 h at the indicated concentrations. Dose-growth response curves were drawn for (A) C4–2, (B) DU145, (C) PC3, (D) Mahlavu, (E) SNU475, (F) MDA-MB-231, (G) T47D, (H) MCF10A cell lines. The experiments were carried out as multiple independent biological replicates with three technical replicates. Absorbance values were normalized to DMSO- treated controls. The means of three independent biological replicates are shown for C4–2, DU145, Mahlavu and MDA-MB-231. The means of four independent biological replicates are shown for PC3, SNU475, T47D and MCF10A. Statistical analysis was performed by two-way ANOVA followed by Tukey’s multiple comparisons test. Error bars depict SEM ****p < 0.0001.

In addition, the enhanced potency of the biotin conjugated compounds (**C160** and **C161**) compared to the parental compound (**EB38**) was reflected in the lower IC_50_ values of **C160** and **C161** across the panel of tested cell lines (Table 1).

**Table 1:** IC50 ± SEM Values of Different Cell Lines (uM)

cell line	EB38	C160	C161
C4–2	2.93 ± 0.74	0.89 ± 0.28	0.67 ± 0.2
DU145	7.49 ± 0.48	1.39 ± 0.17	1.76 ± 0.39
PC3	6.15 ± 0.88	1.41 ± 0.27	1.63 ± 0.1
MAHLAVU	11.41 ± 0.66	2.73 ± 1.04	3.56 ± 1.67
SNU475	4.61 ± 0.33	1.48 ± 0.23	1.78 ± 0.35
MDA-MB-231	4.87 ± 0.30	3.05 ± 0.50	3.1 ± 0.33
T47D	2.11 ± 0.14	0.52 ± 0.05	0.52 ± 0.03
MCF10A	10.74 ± 1.65	6.10 ± 2.4	8.14 ± 3.17

To elucidate the molecular mechanisms underlying the superior antiproliferative effects of **C160** and **C161**, we next focused on the Mahlavu liver cancer cell line, where the antitumor effects of the lead compound were previously demonstrated in vitro as well as in vivo xenograft models.^16^ Cells were separately treated with 1 or 5 μM of **EB38**, **C160**, or **C161** for 48 h, followed by protein extraction. Immunoblot analysis revealed that among the pathways tested, treatment with 5 μM **EB38** reduced the phosphorylation of JAK1, STAT1 and AKT. This suggests the dual inhibition of both the JAK/STAT and PI3K/AKT pathways (Figure 4). Strikingly, even low doses (1 μM) of **C160** led to robust inhibition of both pSTAT1 and pAKT, suggesting enhanced potency at the signaling level compared to the parent compound.

**Figure 4:**
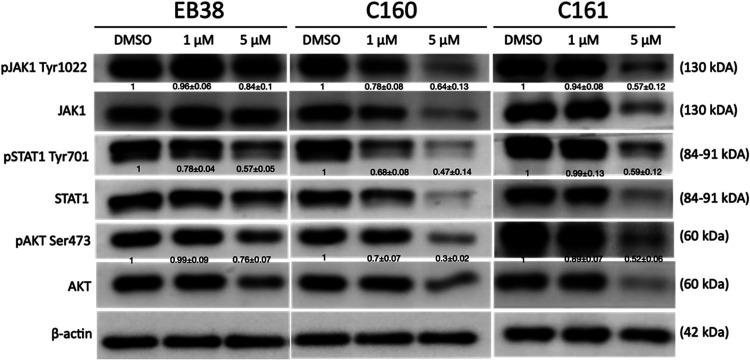
Long-term (48 h) incubation of Mahlavu cells with the diaryl isoxazole compound **EB38** and its biotinylated derivatives **C160** and **C161** inhibit the JAK/STAT and the PI3K pathways. Mahlavu cells that were treated with 1 μM and 5 μM doses of compounds for 48 h. Western blot analysis was carried out using the p-JAK1 (Tyr1022), p-STAT1 (Tyr701), p-AKT­(Ser473), JAK1, STAT1, and AKT antibodies. β-actin was used as loading control. A representative blot of multiple biological replicates (*n* = 3) has been shown. Densitometric quantification of phosphorylated proteins normalized to loading control was performed using biological replicates.

To further investigate whether the PI3K pathway was a direct target of these compounds, we employed a GFP-tagged PIP_3_ biosensor^47^ in C4–2 cells which display active PI3K signaling. The GFP tagged biosensor localizes to the cell membrane when the PI3K pathway is active and is retained in the cytosol as a diffuse signal when the pathway is inhibited. C4–2 cells were treated for 6 h (short-term treatment) with **EB38**, **C160**, or the pan-PI3K inhibitor **GDC-0941** (positive control), followed by epifluorescence microscopy for detection of the GFP signals. While **GDC-0941** markedly decreased the membrane-localized GFP signal, indicative of PIP_3_ depletion, neither **EB38** nor **C160** significantly altered the localization of the biosensor (Figure 5A,B). These findings suggest that the PI3K pathway is unlikely to be the primary direct target of either compound. The observed decrease in the phosphorylation of Akt (Ser 473) may instead have resulted from indirect gene expression changes on upstream receptors, adaptor proteins, or parallel signaling pathways that converge on Akt activation.

**Figure 5:**
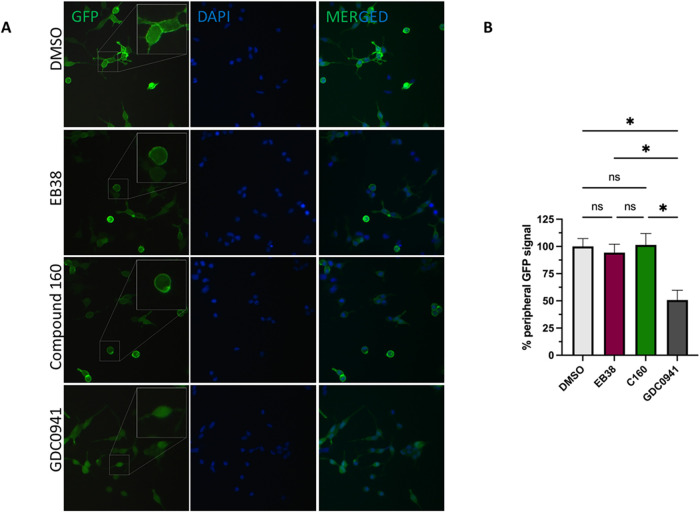
Lack of a direct inhibitory effect of diaryl isoxazole compound **EB38** and its biotinylated derivative **C160** on PI3K signaling. PH^BTK^ – GFP expressing C4–2 cells were incubated with **EB38**, **C160**, and **GDC-0941** as positive control for 6 h. (A) Representative epifluorescence images of C4–2 cells in the green channel and DAPI channel as well as the merged images are shown. Insets in the green channel represent enlarged images of the depicted cells. DAPI was used to stain the nuclei. (B) Peripheral GFP signal as a proxy for PI3K activation was quantified by normalizing the number of cells with peripheral GFP signal to the total number of cells. Experiments were carried out as three independent biological replicates. Statistical analysis was performed by one-way ANOVA. Error bars depict SEM * *p* < 0.05; ns, not significant.

We next focused on the effect of short-term incubation of different cell lines with the isoxazole derivatives on the JAK/STAT pathway. Treatment of Mahlavu, DU145, and C4–2 cells with **EB38**, **C160**, or **C161** revealed that, while **EB38** modestly decreased the phosphorylation of STAT1 (Tyr701), both **C160** and **C161** caused pronounced inhibition of the phosphorylation of STAT1 at low and moderate concentrations (Figure 6A–C). Notably, phosphorylated JAK1 (Tyr1022) levels were also markedly reduced by **C160** and **C161**, suggesting a direct catalytic inhibition of JAK1 kinase activity (Figure 6A,B). In contrast, the effect of **EB38** treatment at equimolar concentrations on pJAK1 was more modest.

**Figure 6:**
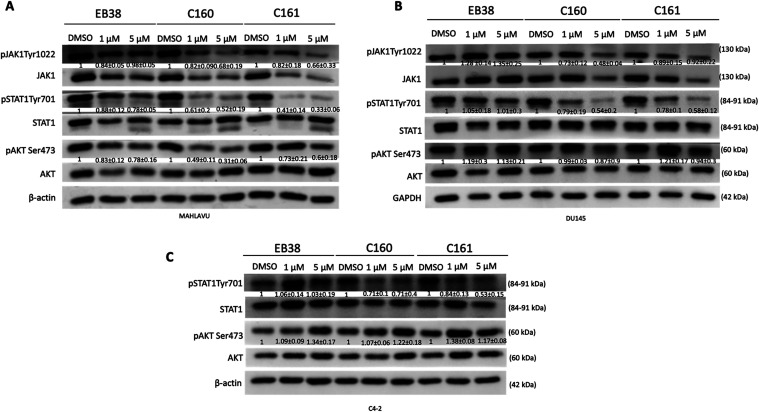
Targeting of the JAK/STAT pathway by the diaryl isoxazole **EB38** and its biotinylated derivatives **C160** and **C161** upon short-term (24 h) treatment. Western blot analysis was with the p-JAK1 (Tyr1022), p-STAT1 (Tyr701), p-AKT­(Ser473), JAK1, STAT1, and AKT antibodies in (A) Mahlavu, (B) DU145, (C) C4–2 cells. The cell lines were separately treated with 1 and 5 μM of the **EB38**, **C160**, and **C161** or vehicle (DMSO) for 24 h. Either β-actin and GAPDH were used as the loading control. A representative blot from multiple biological replicates (For Mahlavu and DU145; *n* = 3, C4–2; *n* = 2) is shown. Densitometric quantification of phosphorylated proteins normalized to loading control was performed using biological replicates.

To determine whether the compound **C160** directly interacts with its putative target, we performed DARTS and CETSA assays. In the DARTS experiment (Figure 7A), treatment with **C160** resulted in a clear, dose-dependent protection of JAK1 from Pronase-mediated digestion, compared with the DMSO control. The pattern of protection was comparable to that induced by **AZD1480**; a known JAK1/2 inhibitor used here as a positive control. Densitometric analysis confirmed that **C160** increased the relative abundance of the undigested JAK1 across the protease dilution series (1:1000 and 1:100), indicating direct physical engagement that reduces proteolytic susceptibility.

**Figure 7:**
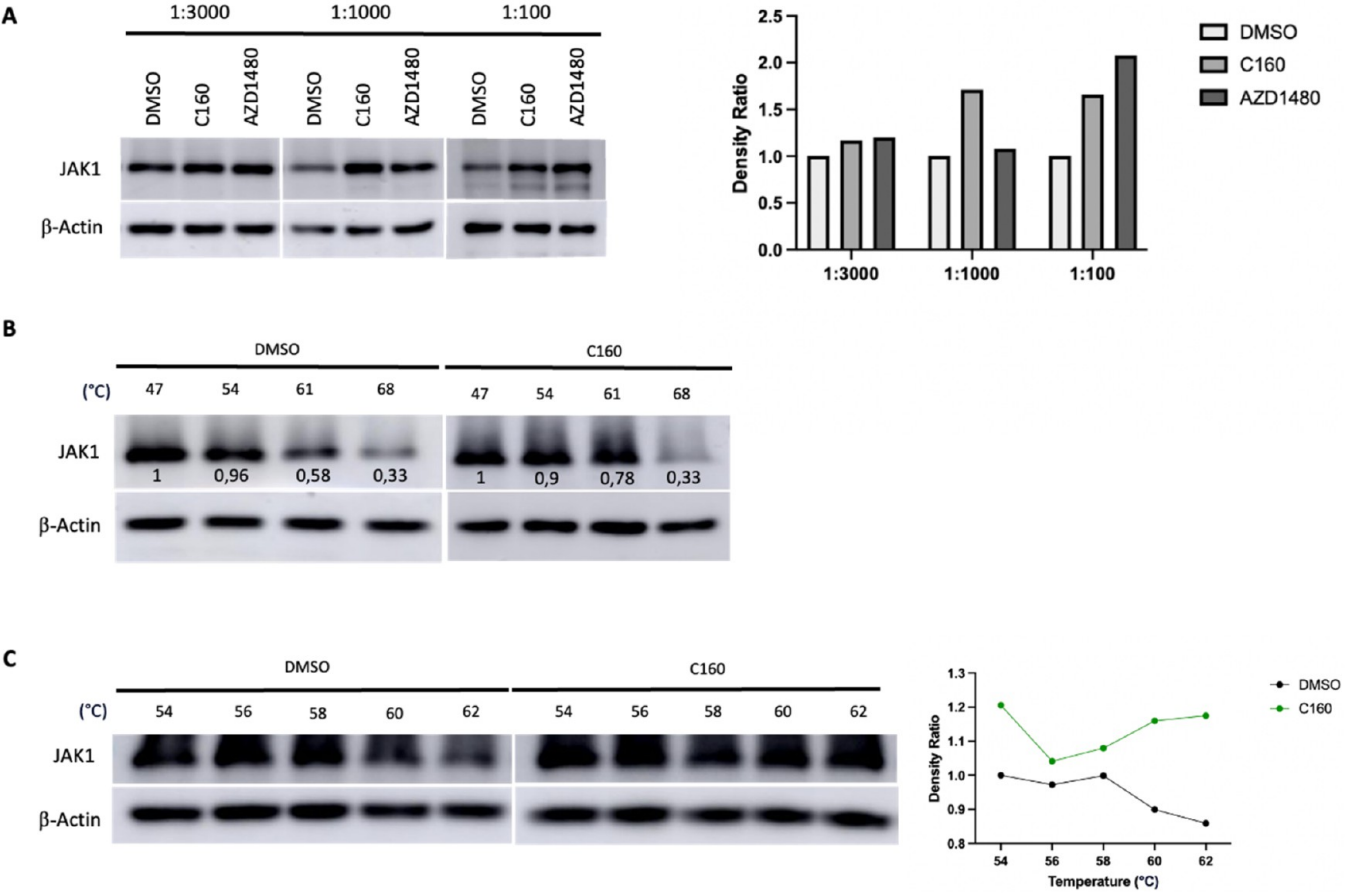
DARTS and CETSA analyses of **C160** binding to its target protein. (A) Drug Affinity Responsive Target Stability (DARTS) assay. Cell lysates were incubated with DMSO, **C160**, or the JAK1/2 inhibitor **AZD1480** followed by limited proteolysis with increasing Pronase dilutions (1:3000, 1:1000, 1:100). Western blotting was performed to assess the proteolytic protection of the target protein. Densitometric quantification is shown on the right, normalized to DMSO controls. Increased band intensity reflects protease resistance and indicates compound–protein binding. (B) CETSA with broad temperature range. Cells were treated with DMSO or **C160** and subjected to cellular thermal shift analysis (CETSA) at 47, 54, 61, and 68 °C. Western blotting was used to assess thermal stability of the target protein across these temperatures. Densitometric quantification of remaining soluble protein is shown on the right. Increased thermal stabilization in **C160**-treated samples indicates compound engagement. (C) CETSA with refined temperature resolution. To precisely map the **C160**-induced thermal shift, cells were treated with DMSO or **C160** and heated at narrower temperature increments (54, 56, 58, 60, and 62 °C). Western blots demonstrate soluble protein levels after heating, with quantification shown on the right. A distinct stabilization window in **C160**-treated samples supports specific binding and thermal protection of the target protein. β-actin is used as loading control.

To further validate target engagement, we conducted CETSA experiments using both broad and fine temperature gradients. In the initial temperature screen (Figure 7B), **C160** treatment shifted the melting profile of the target protein, showing enhanced stability at intermediate temperatures (notably around 61 °C) relative to DMSO. This stabilization suggested the approximate melting temperature range at which the compound–protein interaction occurs.

A refined CETSA with smaller temperature increments (Figure 7C) revealed a more defined stabilization window. **C160** consistently preserved higher levels of soluble protein between 58 and 62 °C, whereas DMSO-treated samples showed a progressive loss of signal. The densitometric profile demonstrated a reproducible thermal shift, supporting the conclusion that **C160** increases the thermodynamic stability of the target protein in cells.

Together, the DARTS and CETSA data demonstrate that **C160** directly engages and stabilizes JAK1. These findings provide strong biochemical evidence for on-target activity of **C160**.

To investigate whether apoptosis contributes to the observed antiproliferative effects of the tested compounds, we examined the cleaved levels of the apoptotic markers PARP, Caspase-3, and Caspase-7. Treatment of all tested cell lines with equimolar doses of **C160** and **C161** induced robust cleavage of these markers (Figure 8A–C). In particular, Mahlavu and C4–2 cells exhibited significant apoptotic responses even at low doses of the biotinylated derivatives (Figure 8A,C) compared to the parent compound (**EB38**), underscoring their enhanced pro-apoptotic potential.

**Figure 8:**
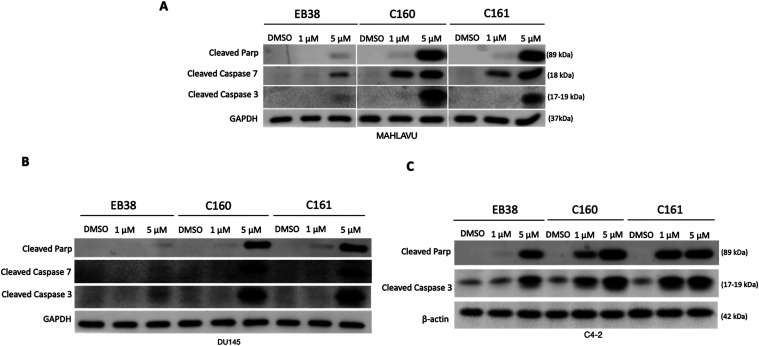
Induction of robust activation of apoptosis in liver and prostate cancer cell lines treated with the biotinylated diaryl isoxazole derivatives. Western blot analysis reflects robust cleavage of PARP, caspase 3, and caspase 7 in (A) Mahlavu, (B) DU145, (C) C4–2 cells treated with 1 and 5 μM doses of the depicted compounds for 24 h. Either β-actin and GAPDH were used as the loading control. A representative blot from multiple biological replicates (For Mahlavu and C4–2 *n* = 3, DU145 *n* = 2) is shown.

To exclude the possibility that the observed effects *in vitro* were due to general cytotoxicity mediated by the compounds, we evaluated the safety profiles of **EB38**, **C160**, and **C161** via zebrafish *in vivo* acute toxicity tests. Larval zebrafish model was selected because of their well-established sensitivity to genotoxic stress during early development.^48^ The exposure window from 2 to 5 dpf was chosen to coincide with the time period of critical organogenesis, parallel to the EURL ECVAM recommendation on the Zebrafish Embryo Acute Toxicity Test Method.^49,50^ The larvae were treated with increasing doses of the compounds (2, 5, 10 μM or 10, 30, 50 μM) for 72 h as in *in vitro* assays (Figure 9A,B). Concentrations of the compounds comparable to those that were used in cellular assays (2, 5, 10 μM) resulted in no morphological abnormalities or developmental defects (Figure 9C) unlike the positive control treatments, i.e., JAK inhibitor **AZD1480** and the multikinase inhibitor sorafenib (**SFB**), the latter with known teratogenicity on zebrafish larvae, under the same concentrations (Figure 9D–F).^51^ While **AZD1480** was not lethal at up to 10 μM, characteristic teratogenic phenotypes were observed at 5 dpf by 0% swim bladder inflation at 5–10 μM, indicating developmental delay, with reduced eye size and cardiac edema formation at 10 μM (Figure 9E,F).^52^ **SFB** treatment, used as a low dose teratogenic positive control, led to shortened body length, eye size reduction, and severe cardiac, yolk sac and eye edema formations at 5 μM, with 100% lethality at 10 μM (Figure 9D–F). On the other hand, the compounds of interest did not cause lethality at up to 50 μM (Figure 9D). A highly significant survival deficit was observed for pooled treatment (**SFB**, *n* = 6 × 18) versus vehicle (DMSO, *n* = 2 × 18) of positive control groups by Fisher’s exact test (*p* = 4.5^–12^). Moreover, high power values of 0.998 and 0.846 were achieved to accurately detect ≥20% and 8% lethality, respectively in between **SFB** versus vehicle treatment groups, using the post hoc power analysis for two-sample proportion test.^53^ Accordingly, a controlled sample size equaling to 18 larvae per treatment group was used for survival analyses of compound toxicity series (three doses per compound for low and high toxicity, each), in line with the 3R’s principles. We found that even at supraphysiological concentrations of the compounds (50 μM, well above the concentrations used in 2D cell proliferation assays), the larvae displayed normal morphology with no signs of toxicity (Figure 9B,C).

**Figure 9:**
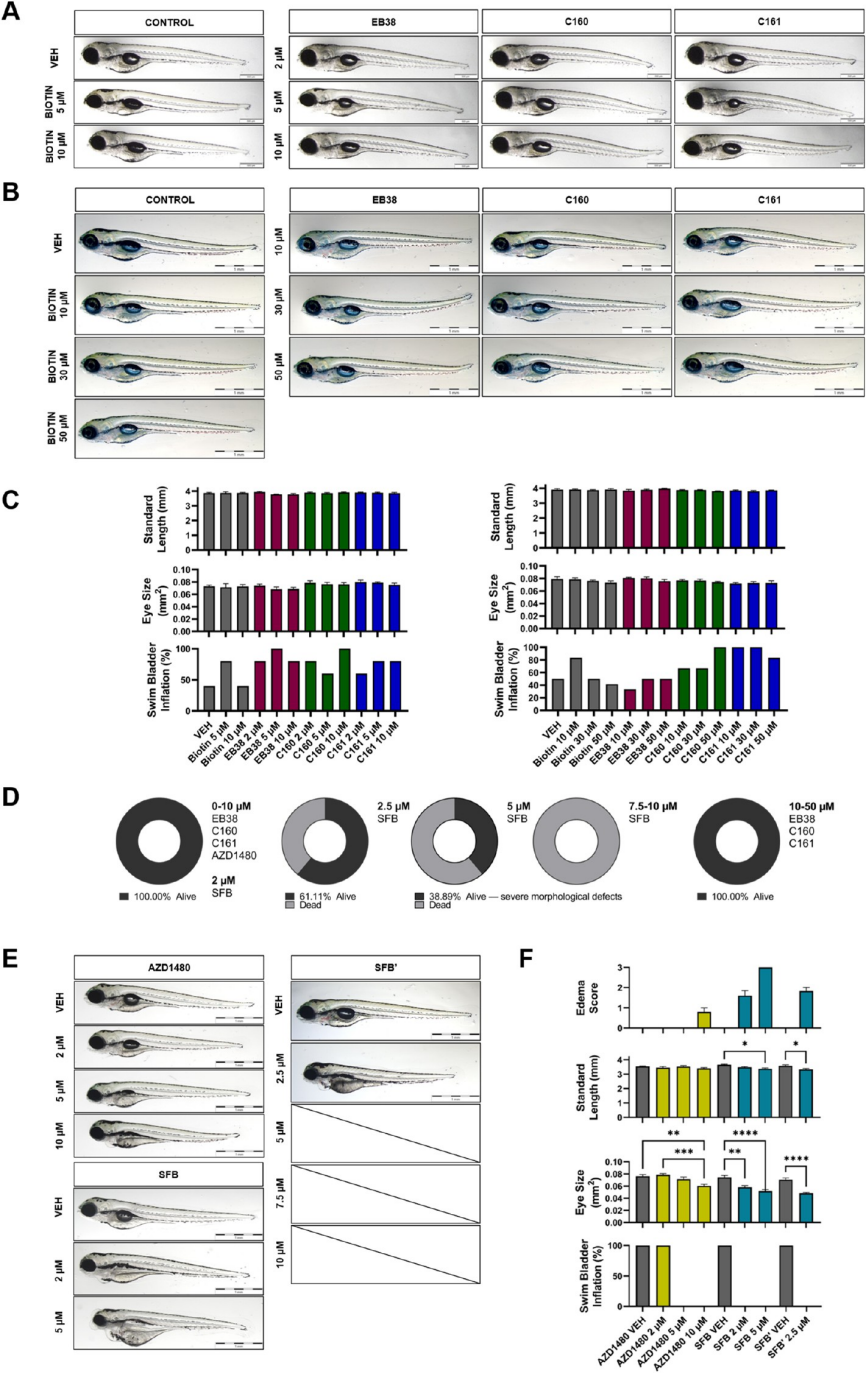
Acute aquatic toxicity test results of the diaryl isoxazole **EB38** and its biotinylated derivatives **C160** and **C161** along with negative (DMSO and Biotin) and positive control (**AZD1480** and sorafenib/**SFB**) treatments on early developing zebrafish larvae treated over a 72-h window from 2 to 5 dpf. (A) Brightfield images of 5 dpf larvae treated with low to intermediate doses of EB38 and derivatives (2–10 μM). (B) Brightfield images of 5 dpf larvae treated with high doses EB38 and derivatives (10–50 μM). (C) Bar graph showing morphometric measurements of larvae treated with low to intermediate doses (*n* = 5 per group) and high doses (*n* = 6 per group) of EB38 and derivatives. (D) Controlled survival rates of 5 dpf larvae treated with different doses of drugs and **SFB**, as positive control (*n* = 18 per group). (E) Brightfield images of 5 dpf larvae treated with **AZD1480** and **SFB** at low to intermediate doses (2–10 μM). (F) Bar graph showing morphometric measurements of larvae treated with **AZD1480** or **SFB** at different doses (*n* = 5 per group). Measurements of 5 dpf larvae were performed by image analysis via ImageJ; error bars depict SEM; edema score 1: cardiac edema only; 2: cardiac and yolk sac edema; 3: cardiac, yolk sac, and eye edema; statistical analysis was performed by one-way ANOVA; * *p* < 0.05; ** < 0.01; *** < 0.001; **** < 0.0001.

Regarding the potential instability of the ester linkage in **C160** and **C161**, we evaluated their plasma stability in both human and mouse matrices. Our results showed that **C160** and **C161** exhibited substantially greater stability in human plasma, retaining 89% and 67% of the parent compound, respectively, after 120 min of incubation. In contrast, both compounds were highly unstable in mouse plasma, with only 0–4% of the parent remaining at the same time point (Table S1). These interspecies differences highlight the need for further mechanistic investigations, particularly to elucidate the contribution of plasma esterases and support the exploration of more stable amide analogs, as well as the careful selection of appropriate species for subsequent pharmacokinetic and in vivo efficacy studies.

The p*K*_a_ values obtained from MolGpKa exhibited that **C160** and **C161** have basic side groups under physiological conditions, with p*K*_a_ values more than 13, rendering molecules to behave as acids. These groups were protonated in the docking analyses. As **EB38** does not have any ionizable groups, this ligand cannot be protonated, also predicted by MolGpKa. For Ruxolitinib, MolGpKa predicted five pKas, ranging from strongly acidic to basic for different side groups.^25^ The basic side groups were protonated in the docking analyses (Table S2).

To explore whether the compounds directly bind to the JAK1 catalytic site, we performed molecular docking simulations using AutoDock Vina, a fast, open-source molecular docking engine that combines efficient multithreaded search with an empirical scoring function to predict ligand–receptor binding.^28,29^ Although all JAK1 structures used had cocrystallized ligands, we included Ruxolitinib, a clinically approved JAK1 inhibitor, as a positive control for comparative purposes.^54^ All ligands, including **EB38**, **C160**, **C161**, and Ruxolitinib, were docked with the protein target in one hundred iterations. Figure 10, panels A displays the binding affinities (kcal/mol) for 20 crystal structures of human JAK1 submitted to the PDB in all of the iterations and the corresponding PDB IDs were listed (Table 2). The average binding affinity of Ruxolitinib across all 20 structures was – 8.37 kcal/mol. Based on this, a threshold of – 8.37 kcal/mol was selected to indicate high-affinity binding and is represented by red dashed lines (Figure 10A). Upon taking all Vina iterations into consideration, **C160** and **EB38** generated affinities with means significantly lower than that of Ruxolitinib (Figure S21, Figure S22). On the other hand, the mean of affinities obtained from **C161** was significantly higher than that of Ruxolitinib, which could be interpreted as the larger size of **C161** having more tendency to introduce steric clashes (Figure 10A).

**Figure 10:**
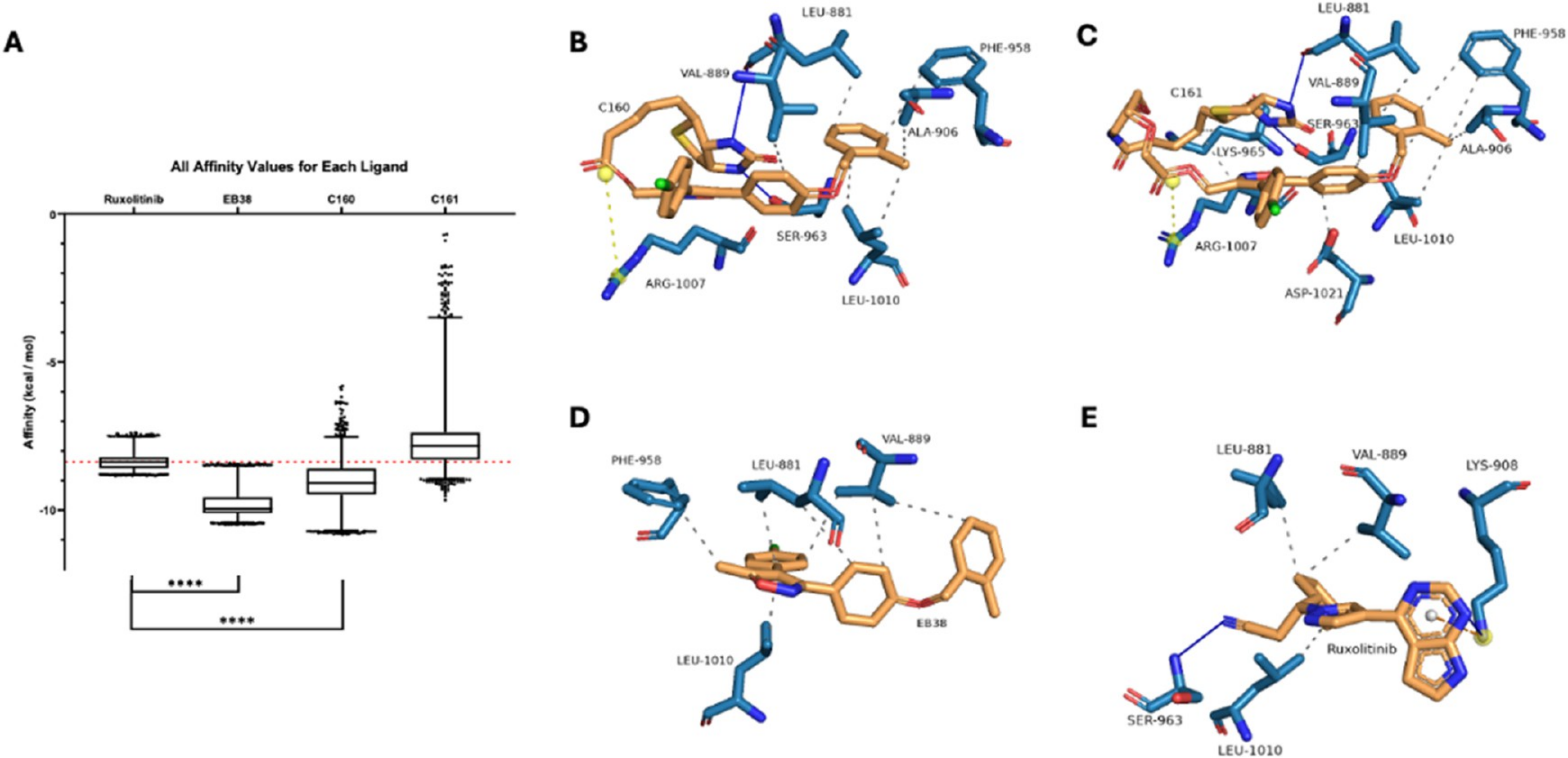
Docking experiments to identify the binding efficacies for Ruxolitinib and the diaryl isoxazole derivatives (**EB38**, **C160** and **C161**) for JAK1. Panel A displays 20 affinity values obtained from one hundred iterations on AutoDock Vina for each ligand to test the significance of obtained affinity scores. The mean affinity scores of **C160** and **EB38** are significantly lower than Ruxolitinib. (****) signifies *p* < 0.0001 in unpaired *t* test with Welch’s correction. The gray dashed lines mark −8 kcal/mol threshold, which is the average value obtained from the docking of Ruxolitinib. The structure that showed the best binding modality for all four ligands was used to depict the different possible binding conformations of (B) **C160**, (C) **C161**, (D) **EB38**, and (E) Ruxolitinib. Gray dashed lines depict hydrophobic interactions, blue continuous lines H-bonds, and yellow dashed lines salt bridges.

**Table 2:** PDB IDs of Crystal Structures

PDB ID	structure number
6N7A	Structure 01
6N77	Structure 02
6N78	Structure 03
6N79	Structure 04
6N7B	Structure 05
6N7D	Structure 06
6RSB	Structure 07
6RSC	Structure 08
6RSD	Structure 09
6SM8	Structure 10
6SMB	Structure 11
5E1E	Structure 12
5HX8	Structure 13
6DBN	Structure 14
6W8L	Structure 15
6GGH	Structure 16
6ELR	Structure 17
6RSH	Structure 18
5WO4	Structure 19
6HZU	Structure 20

Both **C160** and **EB38** demonstrated a favorable binding mode to the catalytic site of JAK1, comparable to that of Ruxolitinib (Figure 10A). In contrast, **C161** exhibited more variable binding affinities, with an average of −7.65 kcal/mol. This variation can be attributed to increased conformational flexibility in the side chains of **C161**. As the largest compound among the four tested inhibitors, **C161** is likely more prone to steric clashes, which may affect its consistent interaction with the catalytic pocket.

The crystal structure that yielded mean affinity values that are less than −8.37 kcal/mol for each ligand and exhibits average affinity values for ligands fitting to the pattern we observed on Figure 10A (**C161** > Ruxolitinib > **C160** > **EB38**) was Structure 13. We selected this structure that could be considered as the overall most representative to show potential binding interactions of all ligands. To find out the most representative pdbqt file for each of the ligand and Structure 13 among their iterations, we selected the pdbqt file whose affinity value is the closest to the median of all one hundred iterations for each ligand. By using these pdbqt files, we utilized PLIP (protein–ligand interaction profiler) to annotate the interactions.^41^ **C160** is stabilized by hydrogen bonds, hydrophobic interactions, as well as a salt bridge formed between Arg1007 of Structure 13 and the carboxylate group of **C160** (Figure 10B). Likewise, **C161** engages in similar interactions, along with a salt bridge formed with the same Arg1007 residue and its carboxylate group (Figure 10C). **EB38** interacts with the catalytic site exclusively through hydrophobic contacts (Figure 10D), whereas Ruxolitinib forms π-Cation interactions and hydrogen bonds in addition to hydrophobic interactions (Figure 10E).

To further validate our docking findings, we employed HADDOCK, a flexible docking method that accounts for conformational adjustments of protein and ligand. For each compound and Ruxolitinib, the top three JAK1 models with the lowest binding affinity scores were selected. HADDOCK scores are calculated by taking the calculated energies of van der Waals, electrostatic, and desolvation into consideration. Buried surface area is a measure for solvent accessibility and how compatible are the ligand and the target pocket on the protein. The positive results of buried surface area indicate the docked complexes are less accessible to solvents in comparison to when they are not docked, an indication of formed complexes being stable. These analyses revealed that EB38, C160, and C161 all form energetically favorable complexes with JAK1, as reflected by their consistently negative HADDOCK scores and favorable van der Waals and electrostatic contributions. When compared directly with ruxolitinib docked into the same JAK1 structure (6RSB, Str 07), C160 displayed a comparable or slightly more favorable overall HADDOCK profile, including a similarly low RMSD and a robust *Z*-score (below −1), indicating a reliable and well-defined binding pose. In contrast, C161 showed modestly weaker HADDOCK scores relative to ruxolitinib, consistent with the steric burden of its larger PEG–biotin extension, yet still remained within the range of stable protein–ligand complexes. Buried surface area values were similar across ligands, suggesting engagement of overlapping binding interfaces. Overall, the HADDOCK results support that C160 binds JAK1 with an affinity and pose quality comparable to ruxolitinib, whereas C161 interacts productively but with slightly reduced docking favorability, in line with its bulkier structure (Table 3).

**Table 3:**
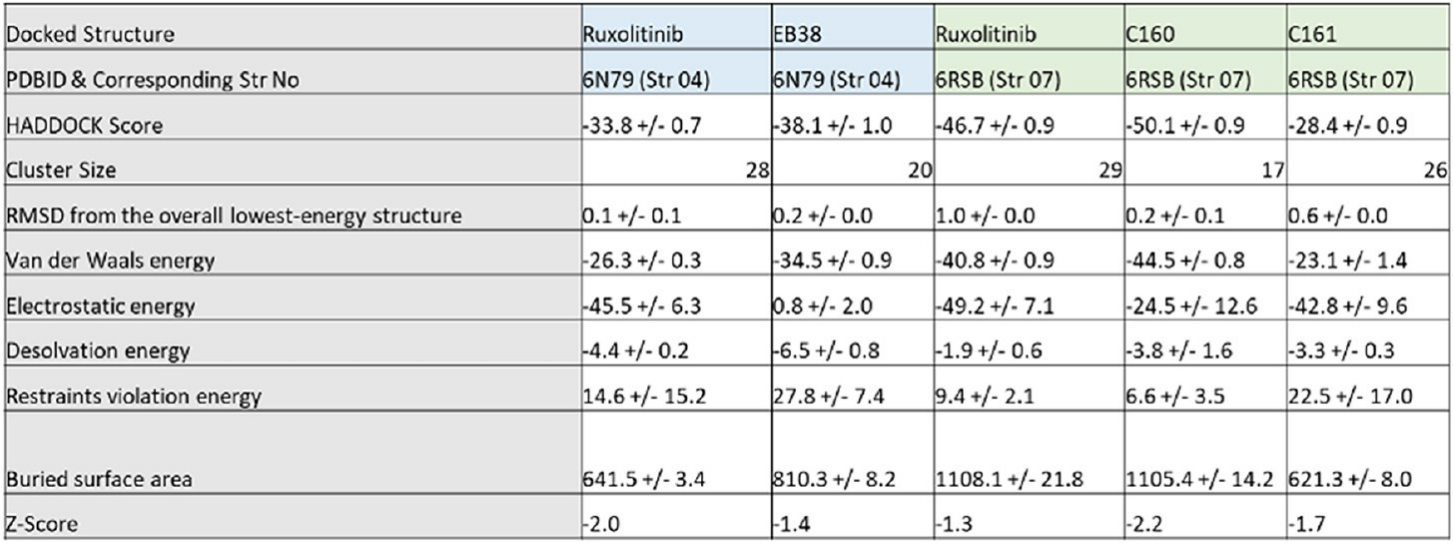
HADDOCK Scores of the Best Three Crystal Structures for the Diaryl Isoxazolc Derivatives

To examine the antiproliferative effects of the compounds in a 3D cancer cell model that is more physiologically relevant, we performed spheroid growth assays using C4–2 cells. **EB38** demonstrated antiproliferative effects at a concentration of 5 μM (Figure 11A). However, **C160** showed superior potency even at lower concentrations (1.5 and 3 μM), as evidenced by an overall smaller size and disrupted spheroid morphology and increased signs of apoptosis (Figure 11A,B). C161 treatment produced an intermediate phenotype, with spheroid inhibition greater than that of EB38 but consistently less pronounced than the effects observed with C160 especially at lower inhibitor concentrations.

**Figure 11:**
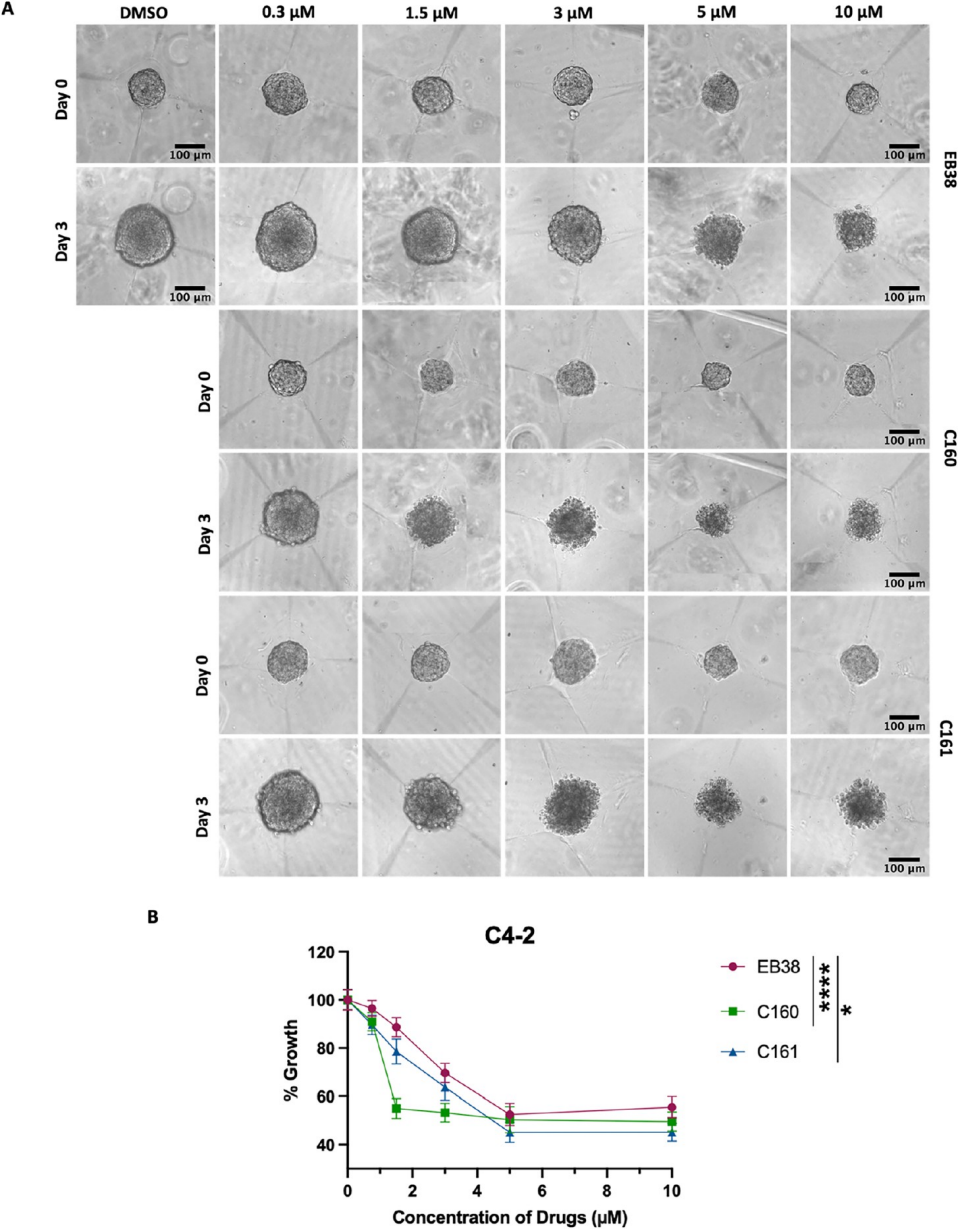
Superior suppressive effect of **C160** compared to **EB38 and C161** on the size of 3D spheroids generated from C4–2 cells. The spheroids were treated with the biotinylated diaryl isoxazole derivative **C160**, **C161** the parent compound **EB38** for 3 days. (A) Representative images of C4–2 spheroids treated with different concentrations of the compounds or vehicle. (B) Dose-response graphs showing growth of spheroids (spheroid size) upon treatment with the compounds or vehicle. The growth percentages were normalized with respect to day 0 measurements, followed by normalization with respect to the vehicle (DMSO) treated spheroids. The experiments were carried out in multiple biological replicates. Statistical analysis was performed by two-way ANOVA followed by Tukey’s multiple comparisons test. Error bars depict SEM *p < 0.05; ****p < 0.0001.

To further assess the functional efficacy of our **EB38**-derived inhibitors in an alternative 3D cellular system, we performed long-term spheroid growth assays using T47D cells. Spheroids were treated with DMSO, **EB38**, its biotinylated derivatives (**C160** or **C161**), or the JAK1/2 inhibitor **AZD1480** as a mechanistic comparator, using two concentrations (0.75 and 5 μM) over a 10-day period.

At 0.75 μM, both **C160** and **C161** produced a pronounced antiproliferative effect (Figure 12A). Spheroids treated with either compound progressively regressed in size, in clear contrast to DMSO-treated spheroids, which continued to expand. **EB38** displayed only modest growth inhibition at this low dose, while **AZD1480** elicited partial suppression without inducing regression. These findings reinforce the notion that the biotinylated analogues retain and further enhance the inhibitory potency of the parent **EB38** compound in 3D culture.

**Figure 12:**
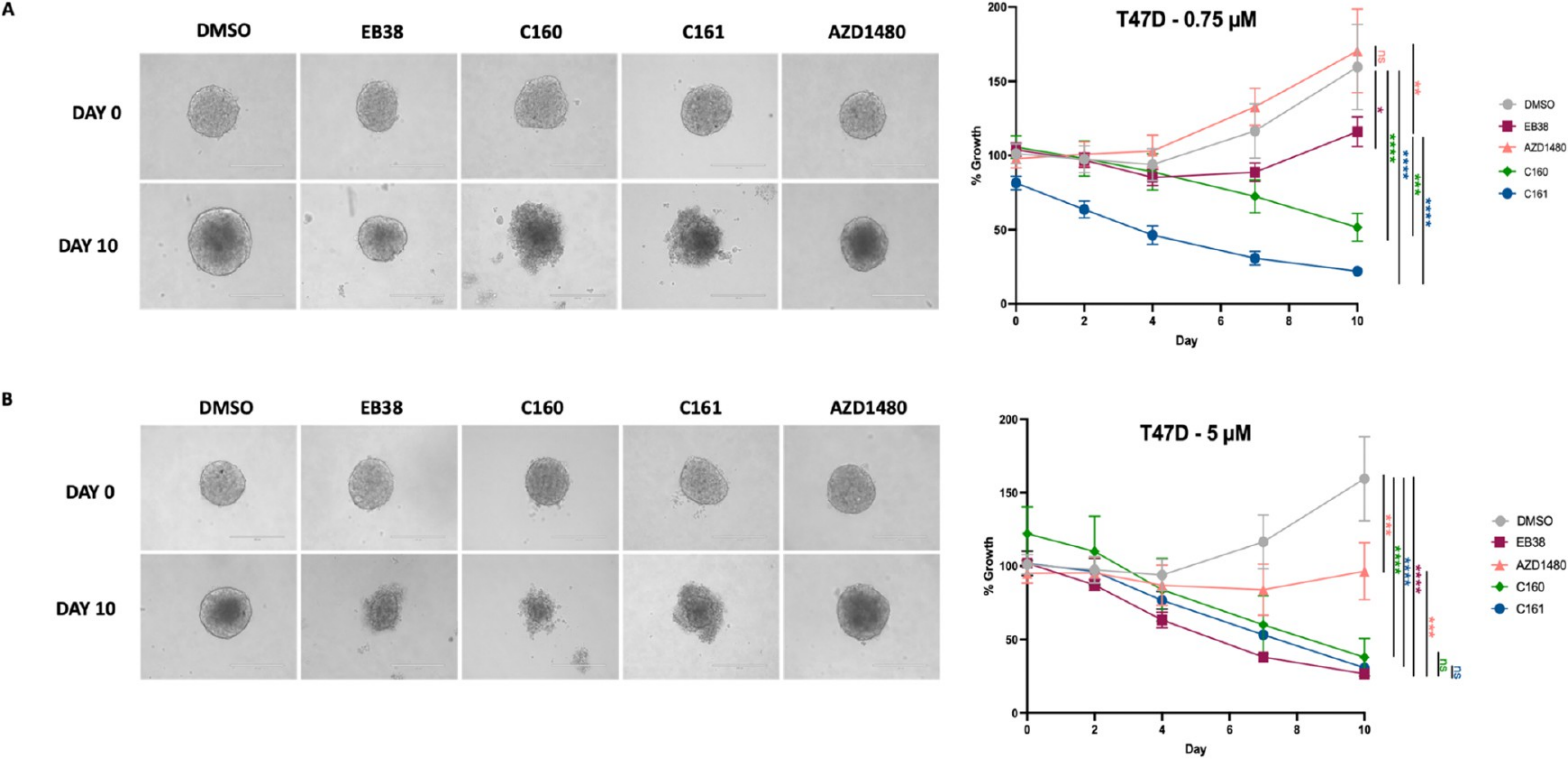
**EB38**-derived inhibitors suppress T47D spheroid growth more potently than a JAK1/2 inhibitor. (A) Low-dose treatment (0.75 μM). T47D spheroids were generated on agarose and treated with DMSO, **EB38** (0.75 μM), its biotinylated analogues **C160** (0.75 μM) and **C161** (0.75 μM), or the JAK1/2 inhibitor **AZD1480** (0.75 μM) for 10 days. Representative bright-field images are shown at the indicated time points. Scale bars: 100 μm. Quantified spheroid growth curves (right) show normalized spheroid area relative to day 0 (mean ± SEM, *n* = 12 spheroids). At 0.75 μM, **C160** and **C161** markedly suppress spheroid expansion, whereas **EB38** and **AZD1480** display only modest inhibitory activity. (B) Intermediate-dose treatment (5 μM). T47D spheroids were treated with DMSO, **EB38** (5 μM), **C160** (5 μM), **C161** (5 μM), or **AZD1480** (5 μM) under identical conditions. Representative spheroid images (left) and normalized growth curves (right) are shown. At 5 μM, **EB38** demonstrates strong growth-repressive activity comparable to **C160** and **C161**. **AZD1480** induces spheroid stasis but does not produce regression. Data represent mean ± SEM of *n* = 12 spheroids per condition. Error bars depict SEM *p < 0.05; **p < 0.01; ***p < 0.001, ****p < 0.0001; ns; not significant.

At 5 μM, **EB38** exhibited strong growth-suppressive activity that closely matched the effects of **C160** and **C161** (Figure 12B). All three inhibitors substantially reduced spheroid growth relative to control, demonstrating dose-dependent activity of the **EB38** scaffold. Under the same conditions, **AZD1480** induced spheroid stasis but did not promote a measurable decrease in spheroid size, underscoring its weaker efficacy in this experimental setting.

Together, these results show that **C160** and **C161** outperform both **EB38** and the JAK1/2 inhibitor at low concentrations, and that **EB38** becomes comparably effective at intermediate dosing, validating the superior potency and 3D-efficacy of the biotinylated derivatives in C4–2 and T47D spheroid models.

## Discussion

In the current study we have demonstrated markedly enhanced antiproliferative and pro-apoptotic activities of the isoxazole-based biotin-conjugated derivatives **C160** and **C161** compared to **EB38**, an isoxazole-containing reference compound. Building upon previous findings that established the cytotoxic potential of **EB38**, our results reveal that strategic biotinylation of **EB38** not only improves the antiproliferative efficacy in 2D monolayer cultures but also translates into superior suppression of growth in 3D spheroid model systems that more closely mimic the in vivo 3D tumor structure.^55^

Biotinylation of small molecules has been widely explored as a drug delivery strategy due to the elevated biotin uptake mechanisms often observed in cancer cells.^56^ Our findings support this approach, as both **C160** and **C161** outperformed the nonbiotinylated **EB38** in inhibiting cell proliferation and inducing apoptosis. The increased potency can be partially attributed to enhanced uptake via biotin transporters such as SMVT that are highly expressed in cancer cells. More importantly, the toxicity assays carried out using zebrafish embryos suggest that these compounds are not broadly cytotoxic, even at doses significantly higher than those required for antiproliferative effects in vitro. This selective activity toward cancer cells underscores the therapeutic promise of these derivatives and further validates biotin conjugation as a means to enhance tumor selectivity without compromising safety.

Mechanistically, our data reveal that **C160** and **C161** exert their effects through potent inhibition of the JAK/STAT signaling pathway, a pathway that is frequently dysregulated in numerous malignancies and associated with resistance to conventional therapies.^57^ Compared to **EB38**, both derivatives achieved greater suppression of pSTAT1 and pJAK1 at lower concentrations, implicating a possible direct interaction with the JAK1 protein. This hypothesis was supported by molecular docking analyses, where both rigid (AutoDock Vina) and flexible (HADDOCK) simulations consistently identified JAK1 as a favorable binding partner, with binding modes and interaction patterns comparable to those observed for ruxolitinib, a clinically approved JAK1 and JAK2 inhibitor.^58^ C161, which carries a PEG–biotin extension, exhibited weaker predicted affinity for JAK1 in AutoDock Vina compared with ruxolitinib and the smaller isoxazole derivatives. This reduced affinity is attributable to the increased steric bulk of the PEG–biotin moiety, which can restrict optimal ligand accommodation within the ATP-binding pocket and introduce steric clashes that limit productive interactions. However, biochemical validation confirmed that both **C160** and **C161** elicited more pronounced downregulation of JAK1 signaling components than **EB38**. Given the high degree of structural conservation among JAK family kinases, definitive selectivity within the JAK family cannot yet be concluded and will require systematic profiling against JAK2, JAK3, and TYK2.

Notably, our investigations using a PIP3 biosensor^59^ suggest that the PI3K/AKT signaling pathway was not a primary target of **EB38** or its derivatives, despite indications from immunoblotting experiments that these pathways may be affected. The lack of biosensor displacement from the cell membrane to the cytosol following short-term exposure to the compounds suggests that any reduction in pAKT levels was likely to be indirect, rather than through any inhibition of PI3K, possibly downstream of JAK1 inhibition or due to compensatory signaling alterations.^60^ This clarification highlights the importance of using orthogonal validation strategies to dissect pathway-specific effects of novel compounds.

DARTS analysis showed that **C160** increases the protease resistance of JAK1, indicating a direct interaction that stabilizes the protein against enzymatic degradation. Consistently, CETSA assays demonstrated a measurable upward shift in JAK1 thermal stability in the presence of **C160**, first detected in the broad-range screen and then confirmed with fine-resolution temperature points. Together, these results provide orthogonal evidence that **C160** directly engages JAK1 and alters its conformational stability, supporting its on-target activity within the inhibitor series, while we acknowledge that formal enzymatic IC_50_/*K*_i_ measurements were not determined and remain an important direction for future mechanistic studies.

The pro-apoptotic activity of **C160** and **C161**, as evidenced by the cleavage of PARP and caspases 3 and 7, further distinguishes these biotinylated compounds from their parent scaffold. This enhanced apoptotic signaling is consistent with JAK1 pathway suppression, which has been previously associated with sensitization to apoptotic cell death in various tumor types.^61^ The robustness of these effects across liver, prostate, and breast cancer models emphasizes the broad applicability of these derivatives in various antitumor therapy settings. Another point of interest is the potential role of Arg1007 within the JAK1 catalytic pocket. Our docking analyses indicate that both C160 and C161 can form stabilizing interactions with this residue, which is situated near the hinge region and may help position inhibitors within the ATP-binding site. Although Arg1007 is not part of any well-established resistance hotspot (such hotspots are predominantly described for JAK2, not JAK1), its involvement in ligand anchoring suggests that it may still influence inhibitor potency and thus merits further investigation.

From a drug development standpoint, our findings offer several compelling insights. First, the improved efficacy of **C160** and **C161** without increased toxicity suggests a favorable therapeutic index, a critical consideration in oncology. Second, the clear linkage between structure (biotin conjugation), mechanism (JAK1 inhibition), and function (proliferation and apoptosis suppression) provides a mechanistic rationale for further preclinical development. Finally, the success of docking experiments in predicting compound-target interactions reinforces the utility of integrated computational and experimental pipelines in current medicinal chemistry approaches.^62^

Despite these advances, several open questions remain. While biotin conjugation improves potency, the precise mechanisms of cellular uptake and subcellular trafficking of these conjugates are yet to be fully elucidated. At present, the available data do not permit discrimination between enhanced uptake and alternative explanations such as altered physicochemical properties, subcellular distribution, or protein binding. In addition, the reduced stability observed specifically in mouse plasma raises the theoretical possibility of context-dependent, partial prodrug-like behavior; however, the molecular basis of this instability and its relevance to intracellular settings, including any contribution of esterases, remain to be determined. Given emerging evidence for transporter-independent pathways for biotin uptake,^18^ further investigation into the intracellular fate of these compounds, potentially involving endocytosis or lysosomal processing, may yield additional mechanistic insight. Moreover, in vivo efficacy and pharmacokinetics remain to be evaluated to determine translational potential.

In summary, our study identifies the biotinylated isoxazole derivatives **C160** and **C161** as promising anticancer agents with enhanced potency and selectivity. Through a combination of cellular, molecular, and computational approaches, we establish JAK1 inhibition as a central mechanism underlying their activity. These findings not only extend the therapeutic utility of the isoxazole scaffold but also reinforce the broader potential of biotin-based targeting strategies in cancer drug development.

## Conclusions

Biotinylated isoxazole derivatives (**C160** and **C161**) exhibited significantly enhanced antiproliferative and pro-apoptotic activity compared to the parent compound **EB38** in both 2D and 3D cancer models. These compounds robustly inhibited JAK1 signaling and induced apoptotic responses across multiple cancer cell lines, without exerting general cytotoxicity in zebrafish embryos. Furthermore, DARTS and CETSA analyses identify JAK1 as a direct and stabilizable target of our compounds. Collectively, our findings support the potential of biotin-conjugated isoxazoles as promising candidates for further development as targeted anticancer agents.

## Supplementary Material



## Notes

^1^H NMR spectrum of intermediates (compounds 4–9); NMR spectra of biotinylated compounds (C160 and C161); UPLC and HRMS analysis of compounds 4–9, C160, and C161; AutoDock Vina binding affinities for compounds ruxolitinib, EB38, C160, and C161; stability of test and control compounds in human and mouse plasma; and p*K*_a_ values of the test and control compounds (PDF)
