# Supplementary material for: Biotinylated Diaryl Isoxazole Derivatives with Enhanced Janus Kinase 1 Inhibitory, Antiproliferative, and Apoptotic Activities in Cancer Cells
Source: ACS Omega. 2026 Jun 17;11(25):36501–19. doi: 10.1021/acsomega.5c08996 (PMC13325105; doi:10.1021/acsomega.5c08996)
Supplement: Supplementary file 1 [file ao5c08996_si_001.pdf]

Supporting Information

Biotinylated Diaryl Isoxazole Derivatives with Enhanced Janus Kinase 1 Inhibitory, Antiproliferative, and Apoptotic Activities in Cancer Cells

Melike Ergüven [1], Hazal Beril Çatalak Yılmaz [2], Deniz Lengerli [3], Rana Acar [1], Servin Bagheralmoosavi [4], Yağmur Öykü Carus Şahin [1], Mehmet Gürçay [1], Burcu Çalışkan [3], Sreeparna Banerjee [2], Gurkan Yesiloz [4], Ozlen Konu [1], Erden Banoglu [3], Onur Cizmecioglu [1]*

[1] Department of Molecular Biology and Genetics, Bilkent University, Ankara, Türkiye.
[2] Department of Biological Sciences, Orta Dogu Teknik Universitesi, Ankara, Türkiye.
[3] Department of Pharmaceutical Chemistry, Faculty of Pharmacy, Gazi University, 06560 Ankara, Türkiye.
[4] National Nanotechnology Research Center (UNAM) Bilkent University, 06800, Ankara, Türkiye.

Table of Contents

^{1}H NMR Spectrum of Intermediates (Compounds 4–9)………………………….. S2
Figure S1. ^{1}H NMR and ^{13}C NMR analysis of Compound 4………………………………… S2
Figure S2. ^{1}H NMR and ^{13}C NMR analysis of Compound 5………………………………… S2
Figure S3. ^{1}H NMR and ^{13}C NMR analysis of Compound 6………………………………… S3
Figure S4. ^{1}H NMR and ^{13}C NMR analysis of Compound 7………………………………… S3
Figure S5. ^{1}H NMR and ^{13}C NMR analysis of Compound 8………………………………… S4
Figure S6. ^{1}H NMR and ^{13}C NMR analysis of Compound 9………………………………… S4
NMR Spectra of Biotinylated Compounds (C160 and C161)…………………….. S5
Figure S7. ^{1}H NMR and ^{13}C NMR analysis of C160………………………………………… S5
Figure S8. HSQC and HMBC (^{1}H-^{13}C) NMR analysis of C160…………………………….. S6
Figure S9. Partial HMBC (^{1}H-^{13}C) spectra of C160………………………………………… S7
Figure S10. ^{1}H NMR and ^{13}C NMR analysis of C161……………………………………….. S8
Figure S11. HSQC and HMBC (^{1}H-^{13}C) NMR analysis of C161……………………………. S9
Figure S12. Partial HMBC (^{1}H-^{13}C) spectra of C161……………………………………... S10
UPLC and HRMS Analysis of the Compound 4-9, C160 and C161……………... S11
Figure S13. UPLC and HRMS chromatograms of Compound 4…………………………….. S11
Figure S14. UPLC and HRMS chromatograms of Compound 5…………………………….. S12
Figure S15. UPLC and HRMS chromatograms of Compound 6…………………………….. S13
Figure S16. UPLC and HRMS chromatograms of Compound 7…………………………….. S14
Figure S17. UPLC and HRMS chromatograms of Compound 8…………………………….. S15
Figure S18. UPLC and HRMS chromatograms of Compound 9…………………………….. S16
Figure S19. UPLC and HRMS chromatograms of C160…………………………………….. S17
Figure S20. UPLC and HRMS chromatograms of C161…………………………………….. S18
Figure S21. AutoDock Vina binding affinities for Compounds Ruxolitinib and EB38……… S19
Figure S22. AutoDock Vina binding affinities for Compounds C160 and C161…………….. S20
Table S1. Stability of Test and Control Compounds in human and mouse plasma…………... S20 S21

Table S2. pKA Values of the Test and Control Compounds…………………………………..

^{1}H NMR Spectrum of Intermediates (Compounds 4–9)

Figure S1. ^{1}H NMR and ^{13}C NMR analysis of **Compound 4**

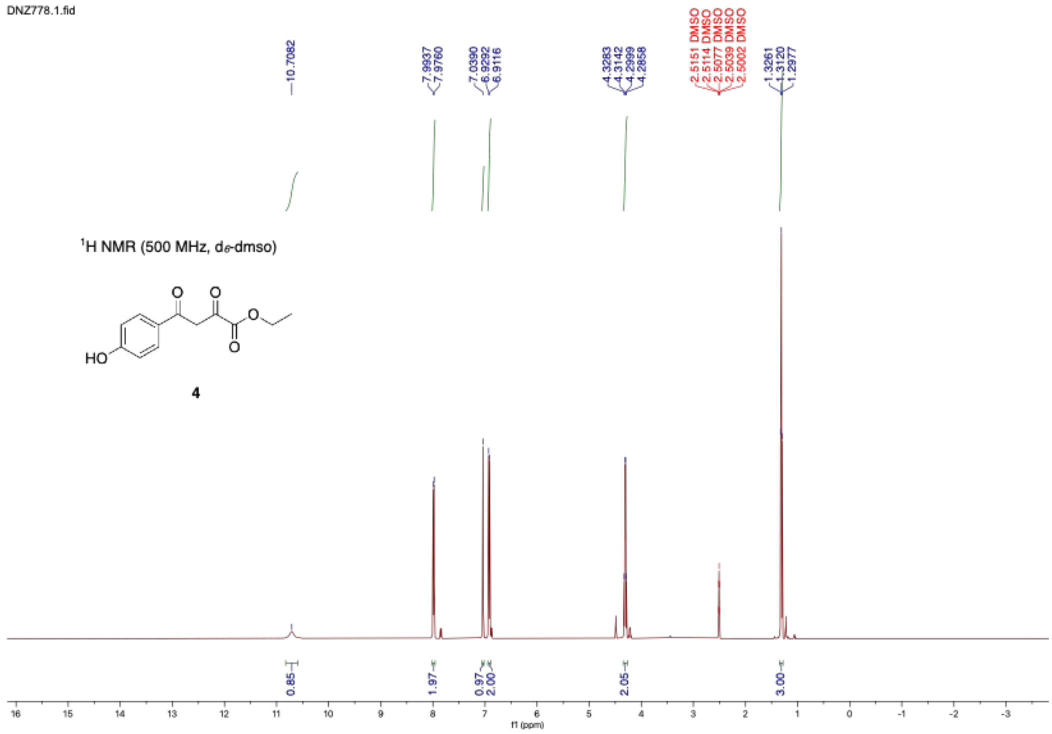

Figure S2. ^{1}H NMR and ^{13}C NMR analysis of **Compound 5**

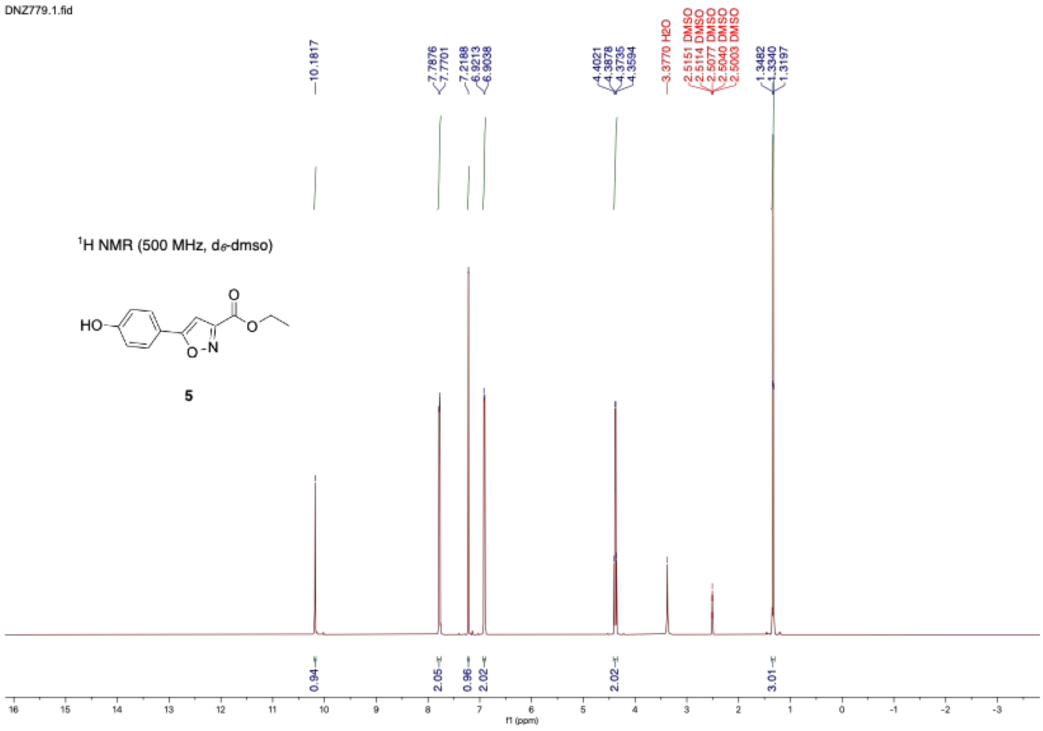

Figure S3. ^{1}H NMR and ^{13}C NMR analysis of **Compound 6**

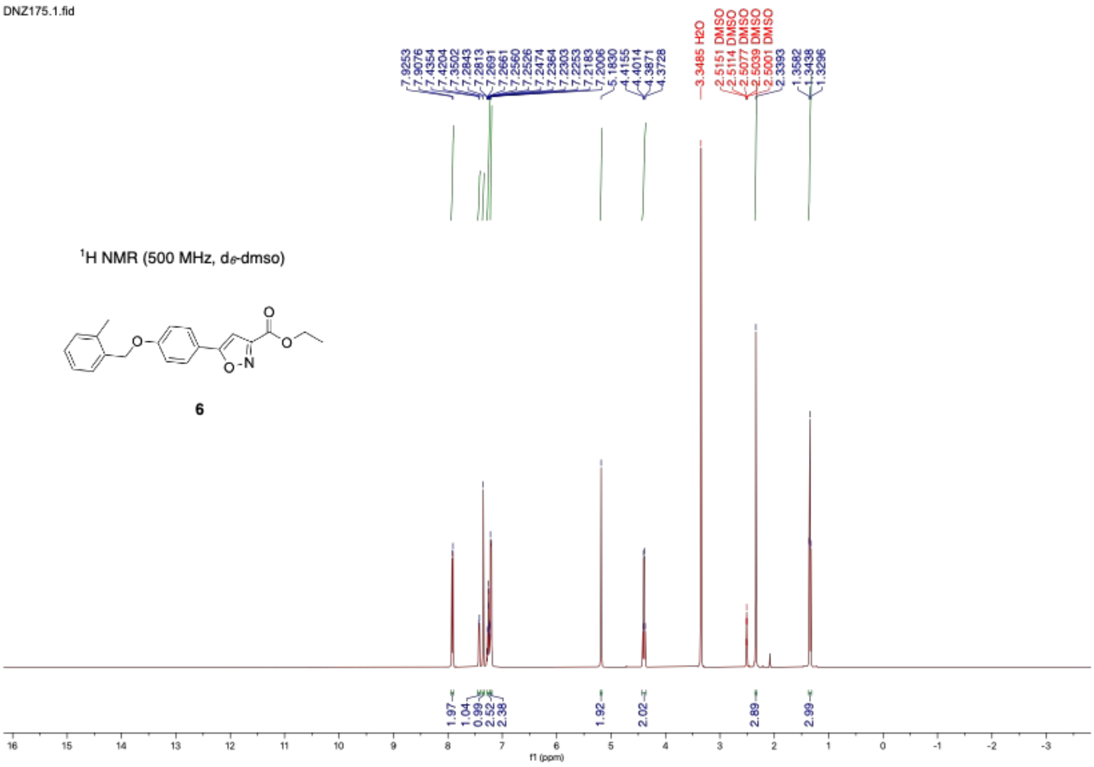

Figure S4. ^{1}H NMR and ^{13}C NMR analysis of **Compound 7**

DNZ178.1.fid

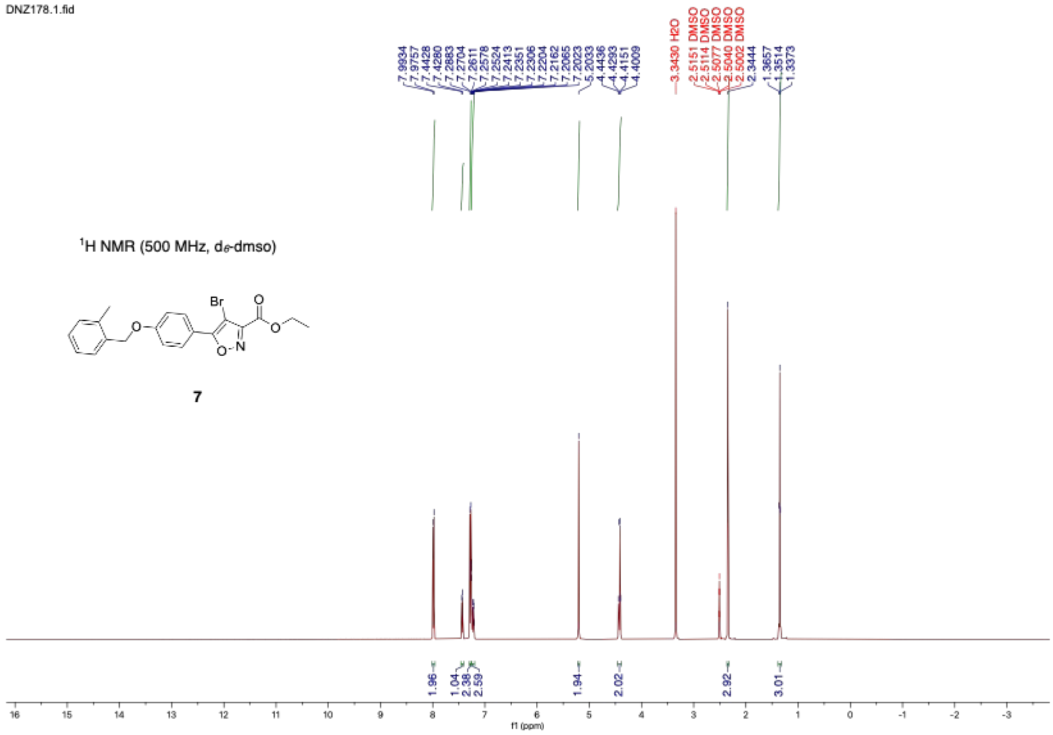

^{1}H NMR (500 MHz, d_6-dmso)

7

Figure S5. ^{1}H NMR and ^{13}C NMR analysis of **Compound 8**

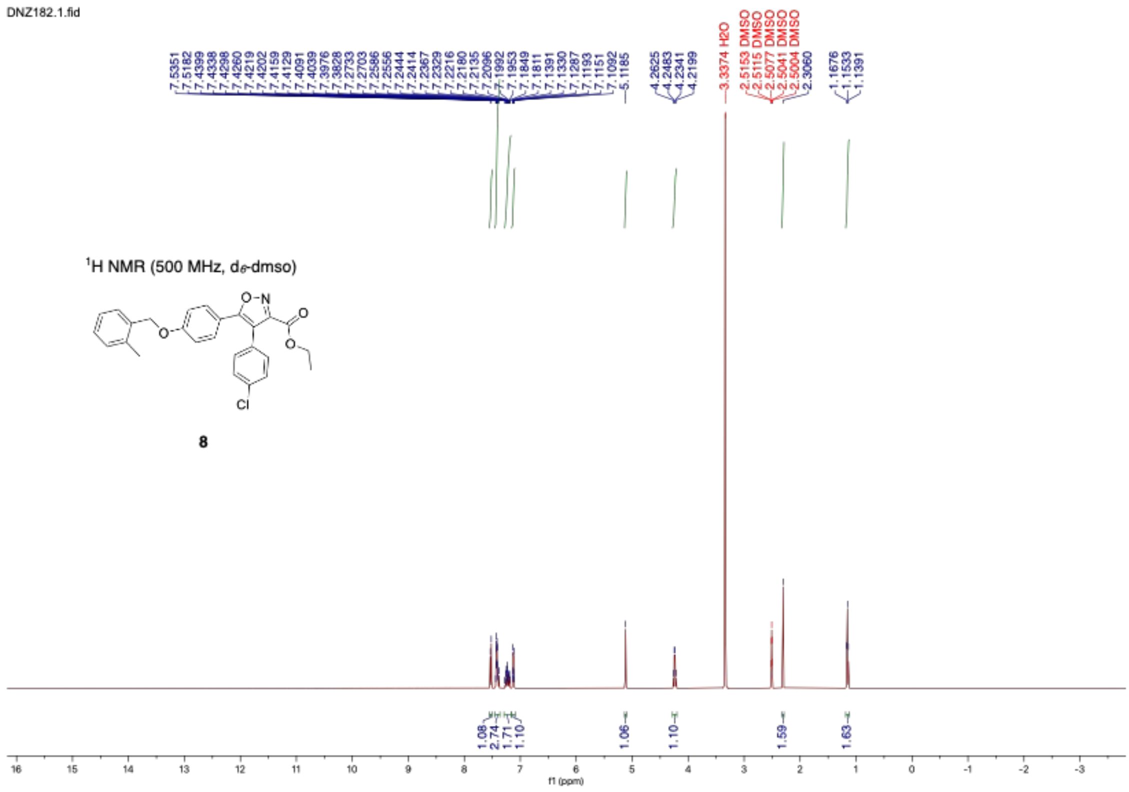

Figure S6. ^{1}H NMR and ^{13}C NMR analysis of **Compound 9**

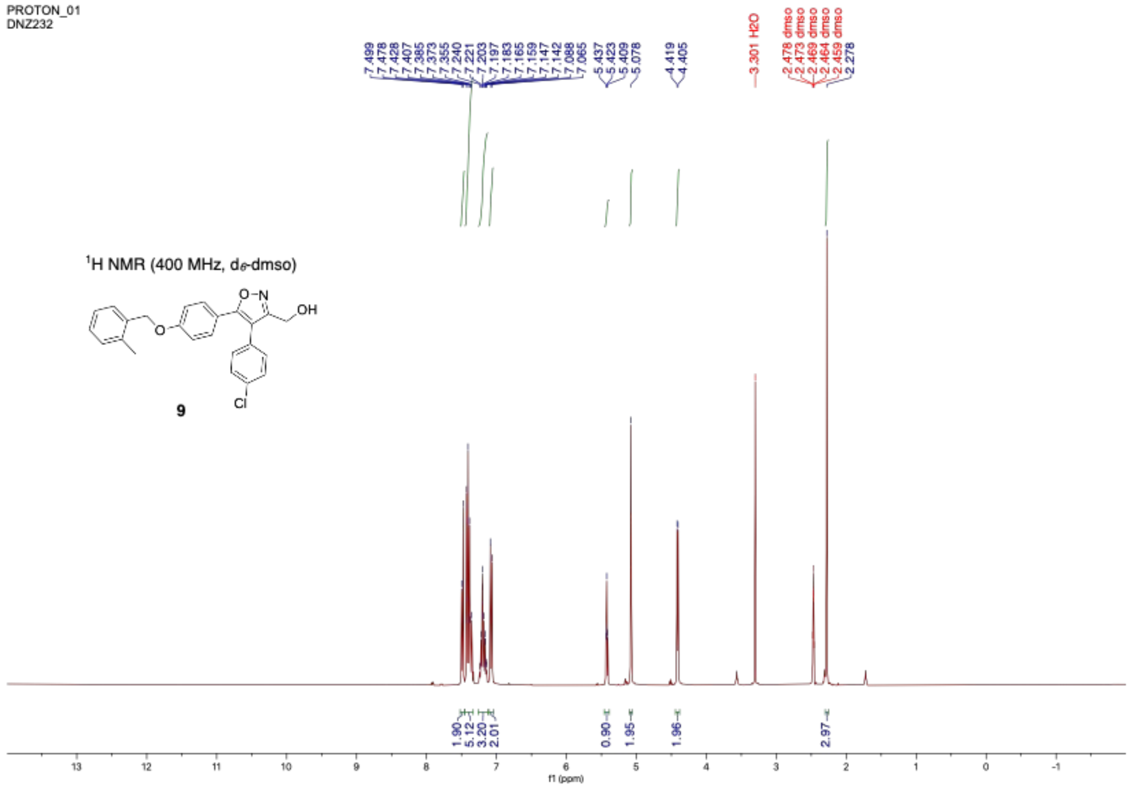

NMR Spectra of Biotinylated Compounds (C160 and C161)

Figure S7. ^{1}H NMR and ^{13}C NMR analysis of **C160**

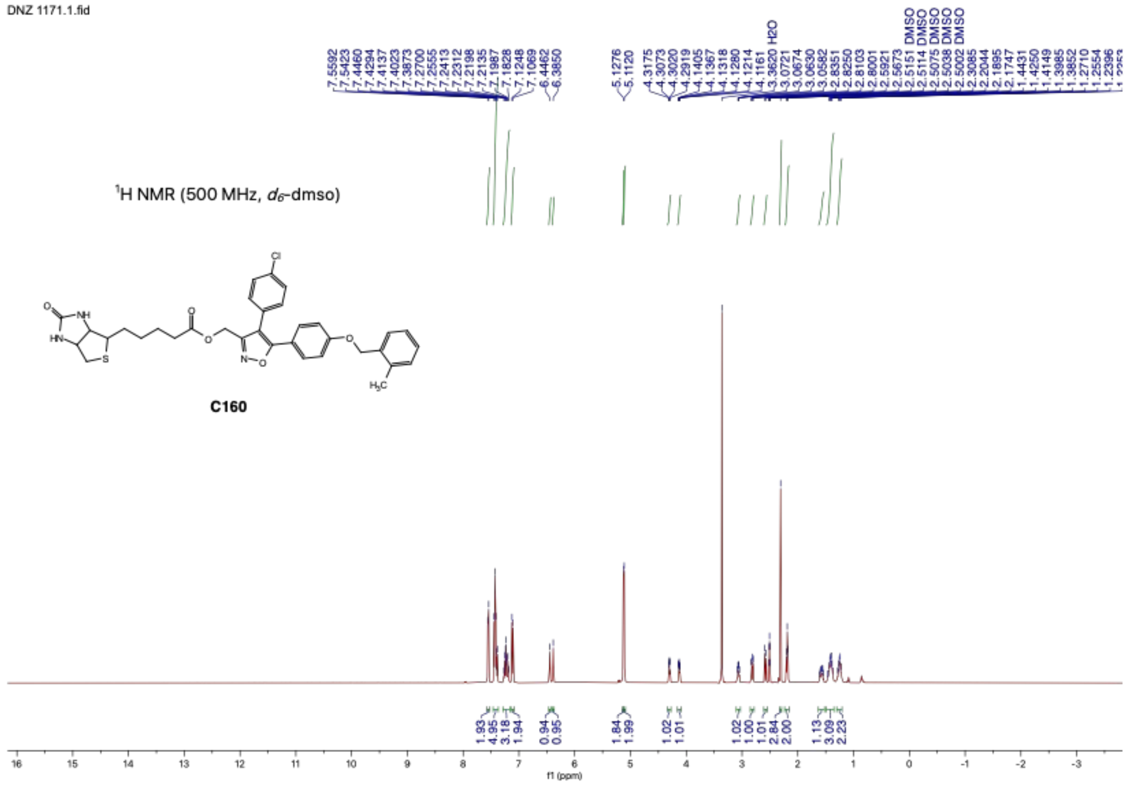

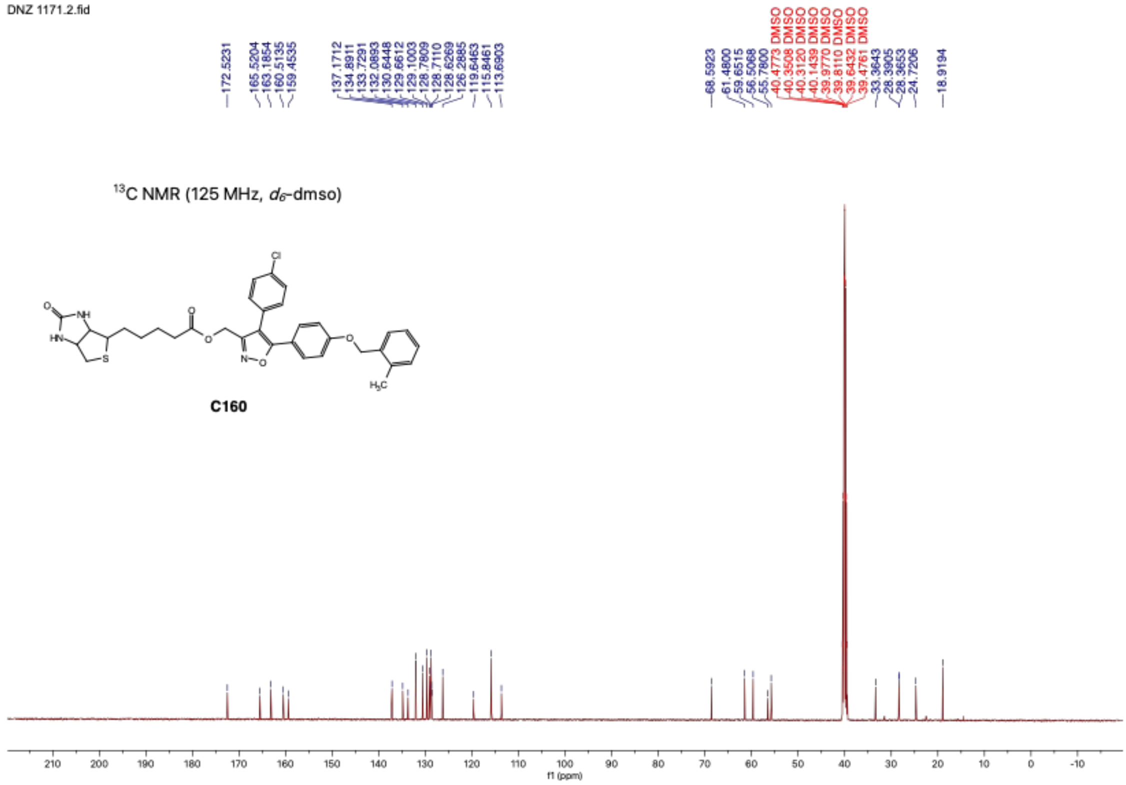

Figure S8. HSQC and HMBC (^1H-^{13}C) NMR analysis of **C160**

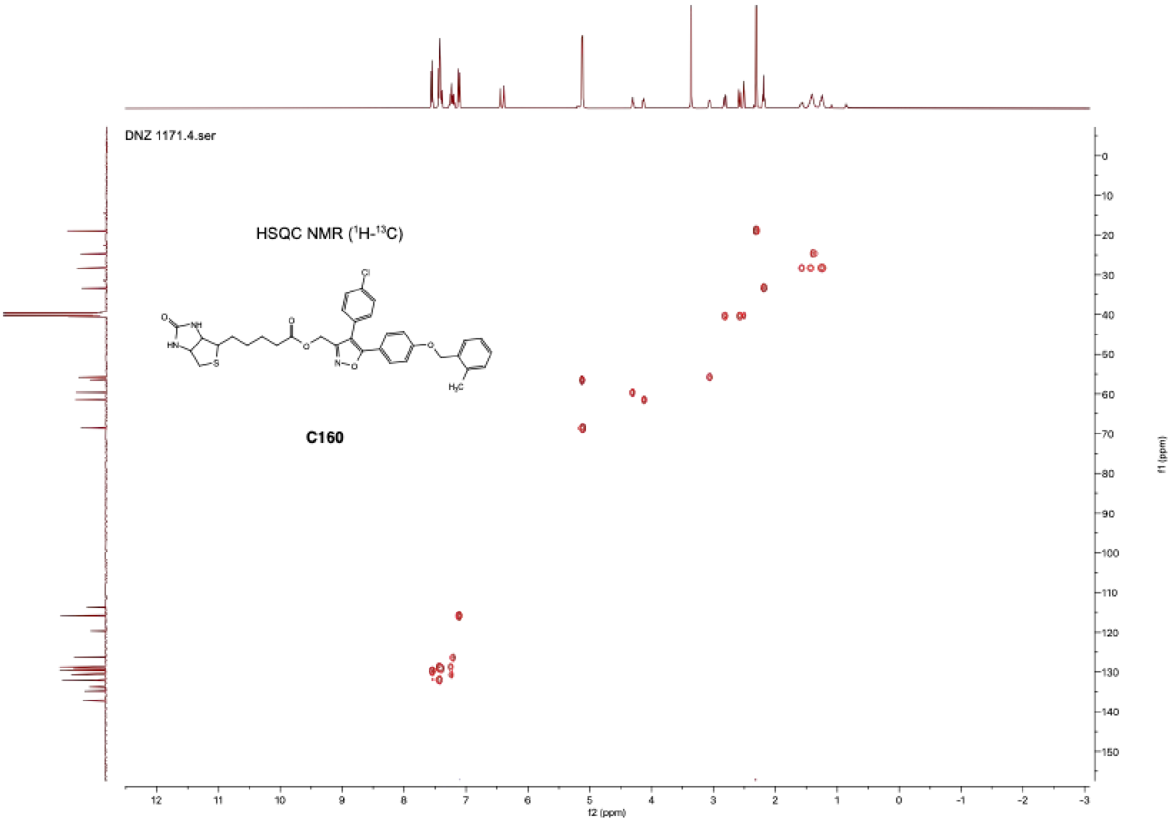

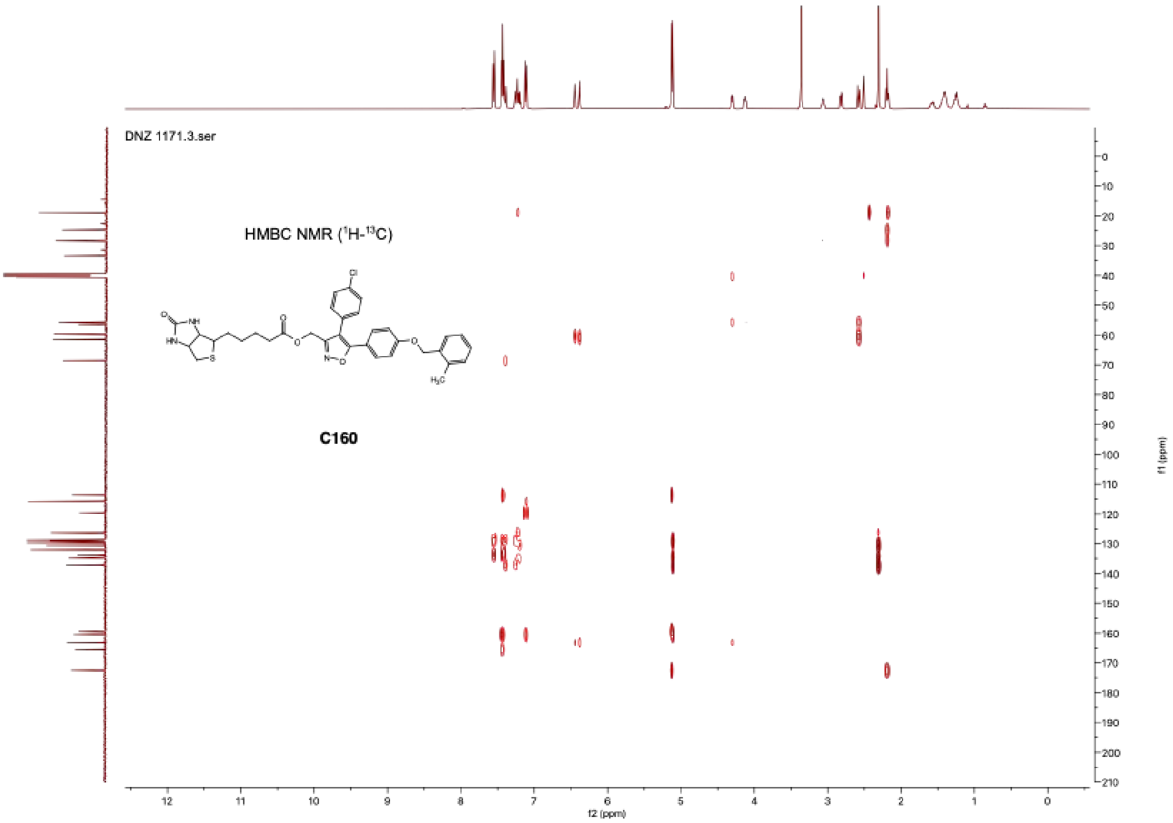

Figure S9. Partial HMBC (^{1}H-^{13}C) spectra of **C160**

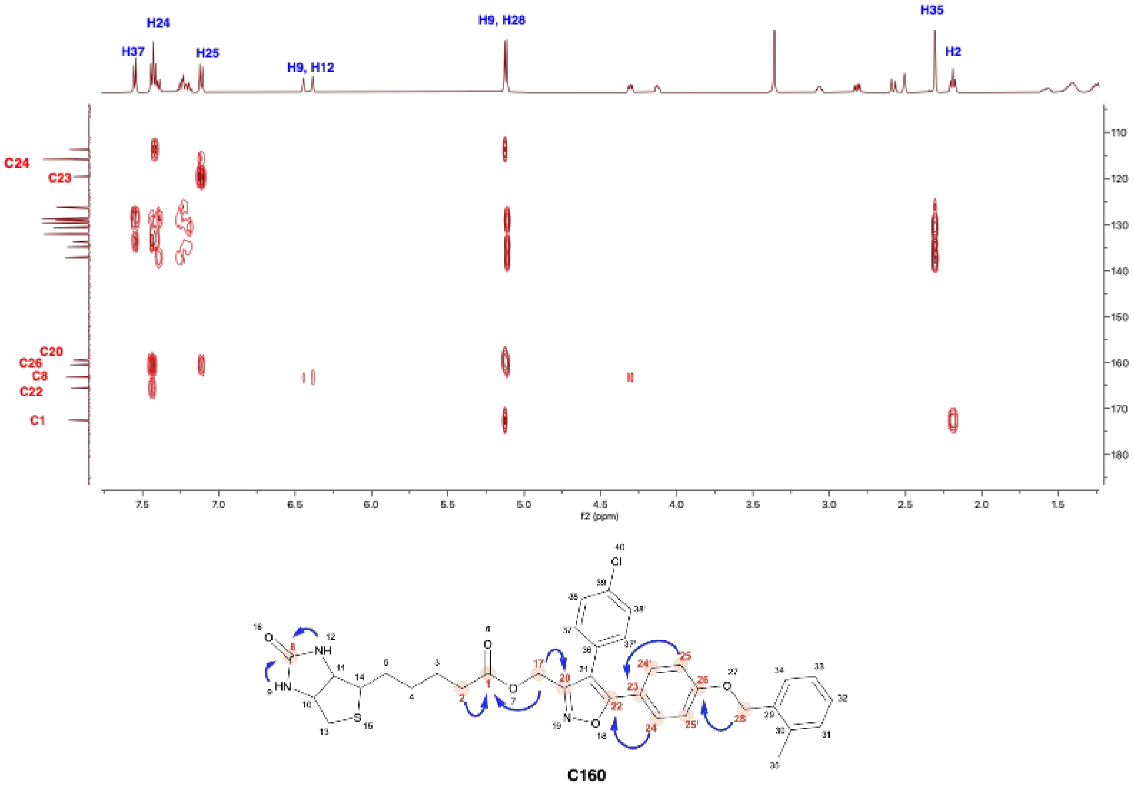

Figure S10. ^{1}H NMR and ^{13}C NMR analysis of **C161**

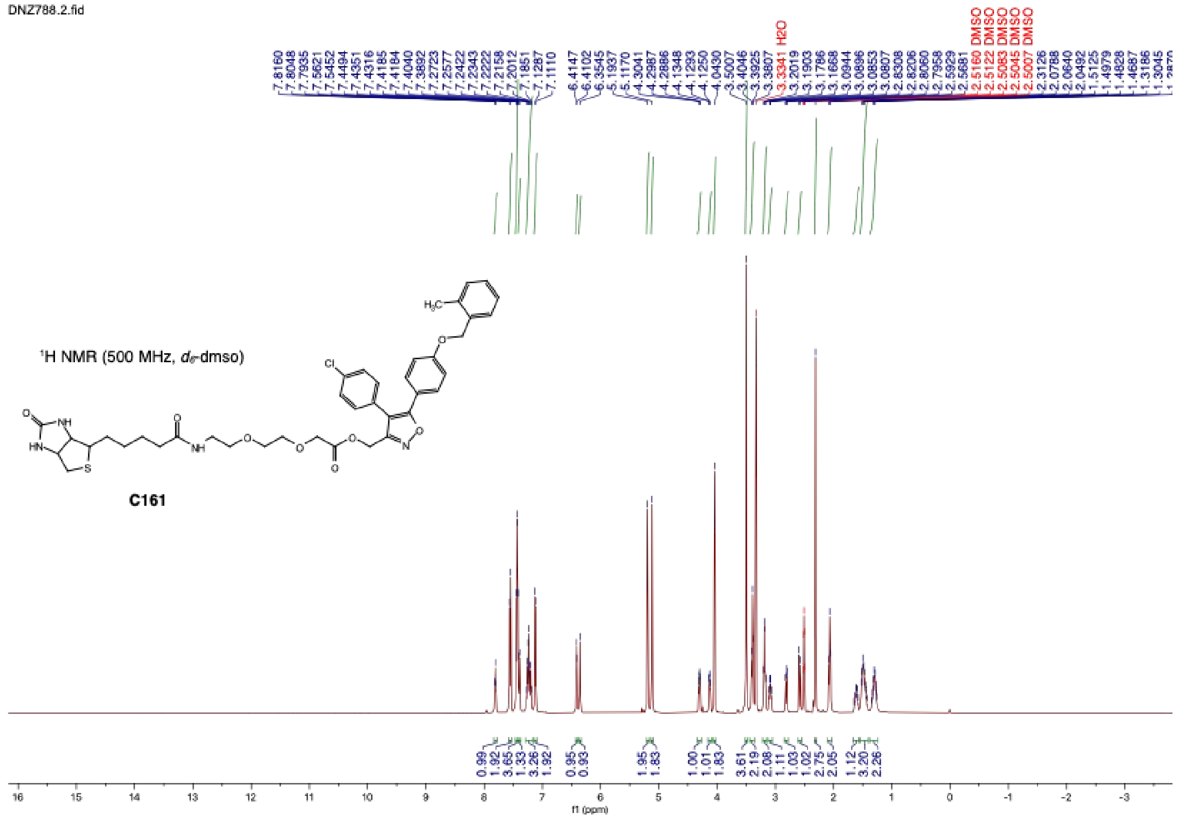

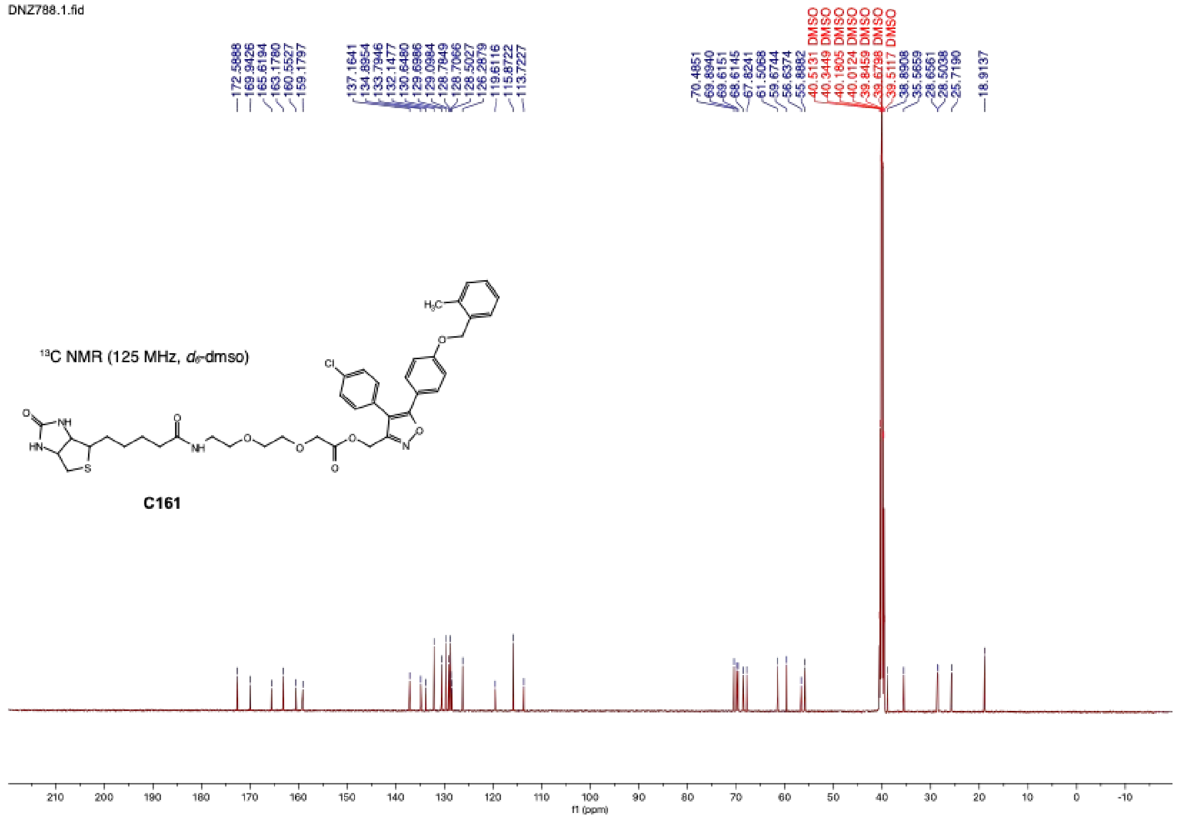

Figure S11. HSQC and HMBC (^{1}H-^{13}C) NMR analysis of **C161**

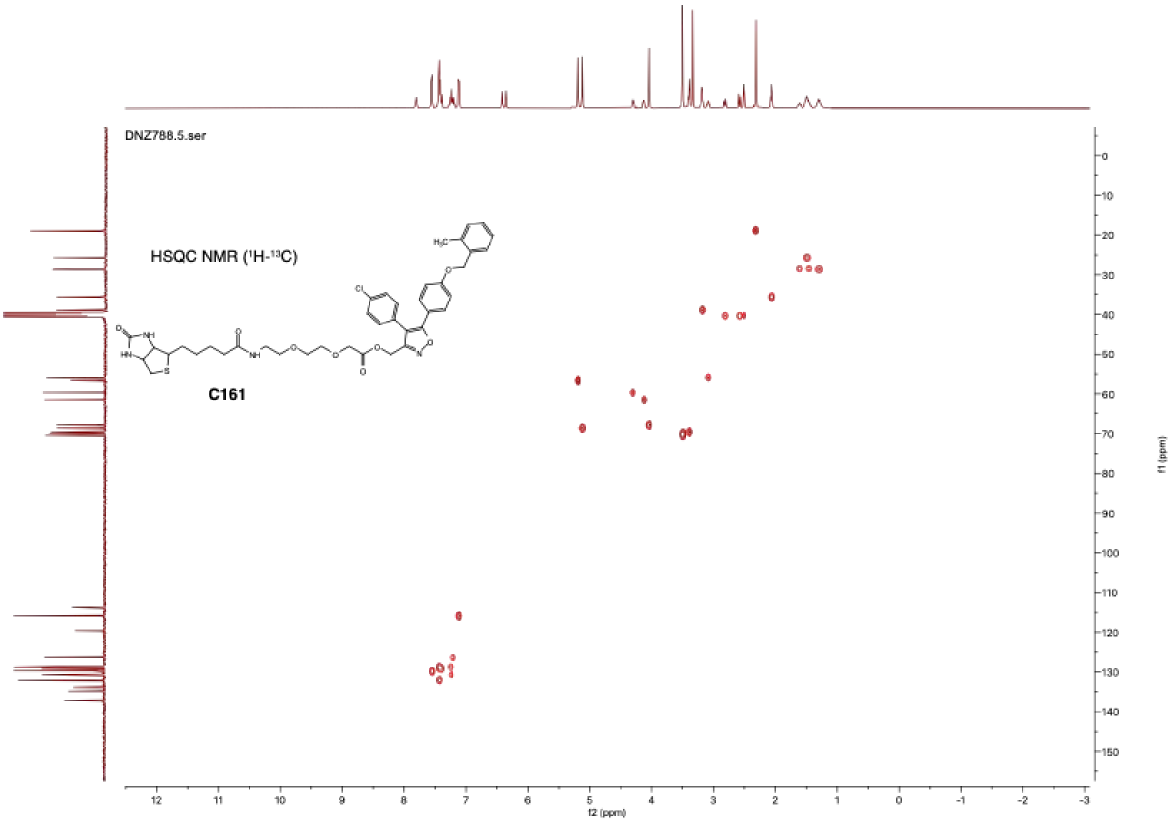

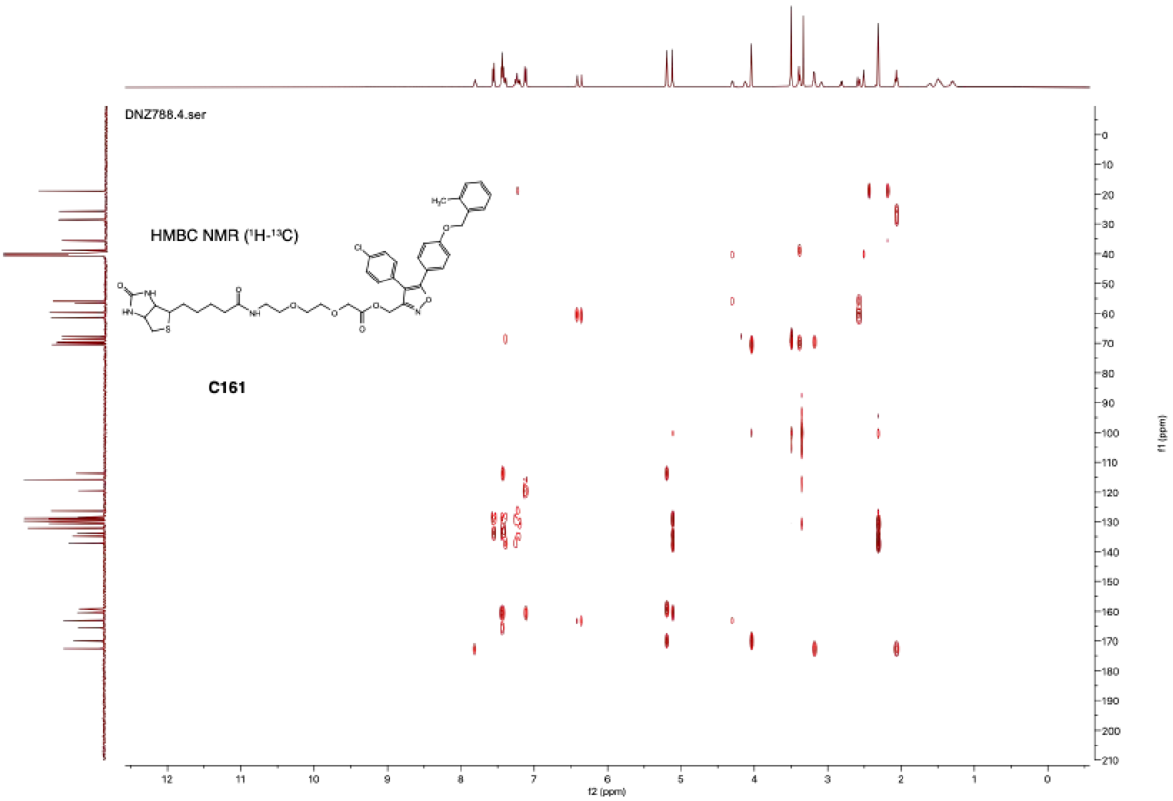

Figure S12. Partial HMBC (^{1}H-^{13}C) spectra of **C161**

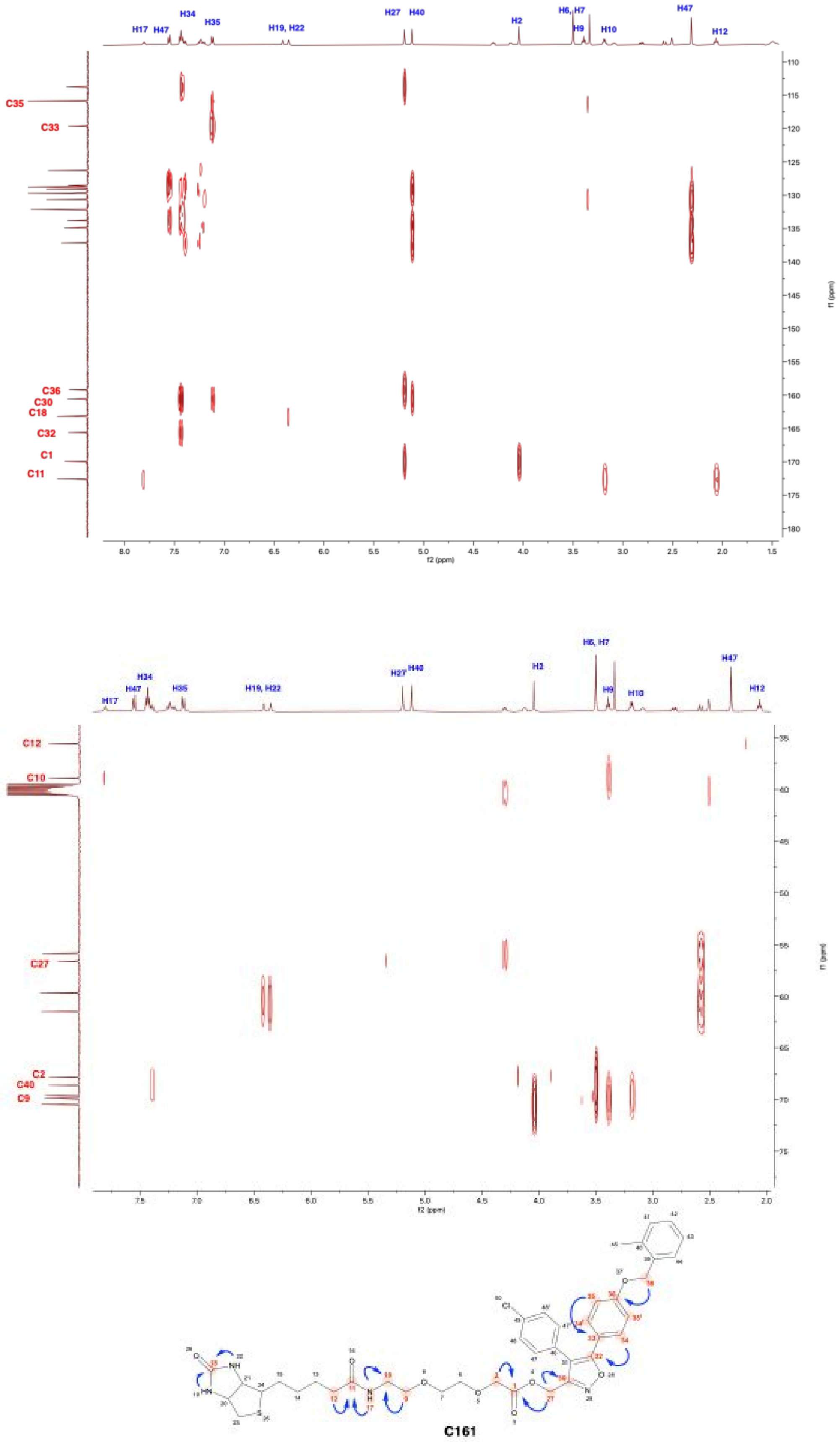

UPLC and HRMS Analysis of the Compound 4-9, C160 and C161

UPLC chromatograms of the synthesized compounds were acquired at a detection wavelength of 254 nm. Reported purity values represent UV-based area%.

Figure S13. UPLC and HRMS chromatograms of **Compound 4**

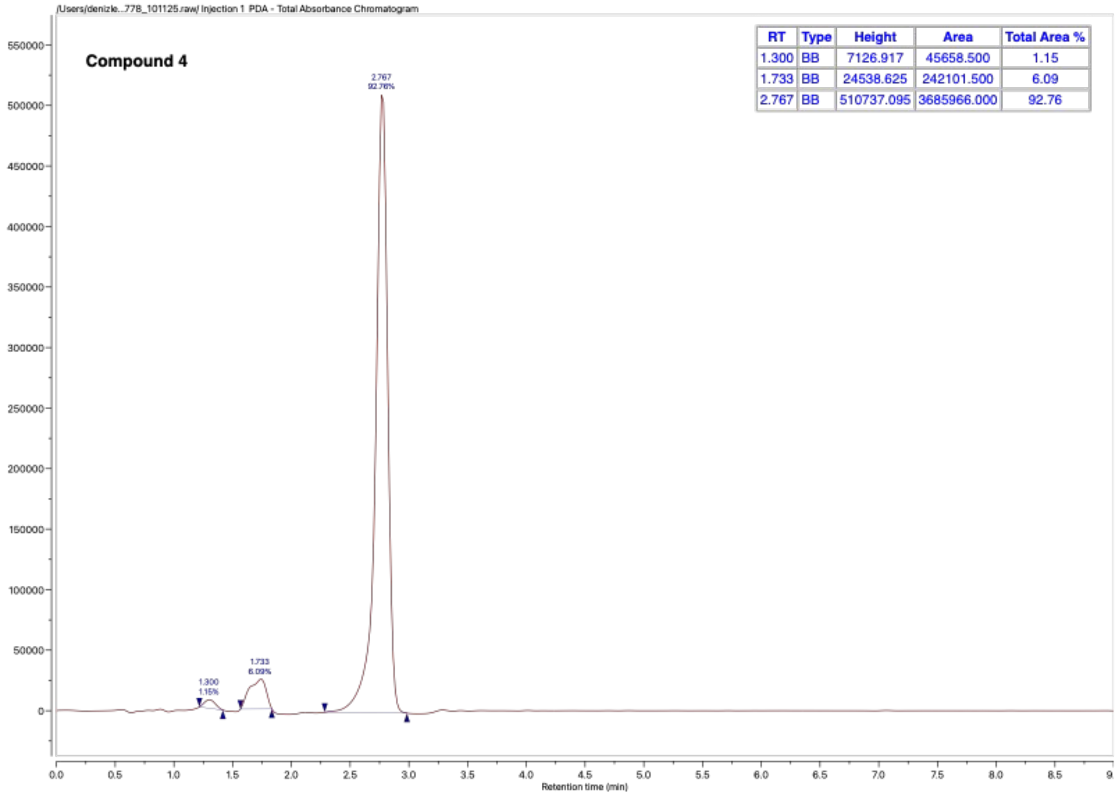

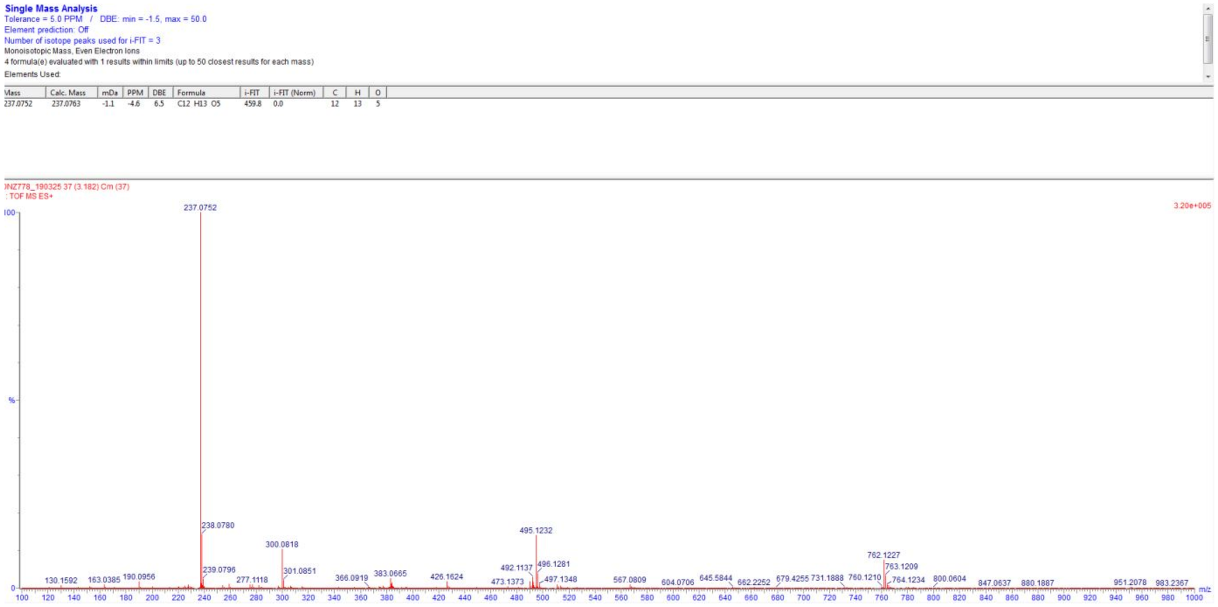

Figure S14. UPLC and HRMS chromatograms of **Compound 5**

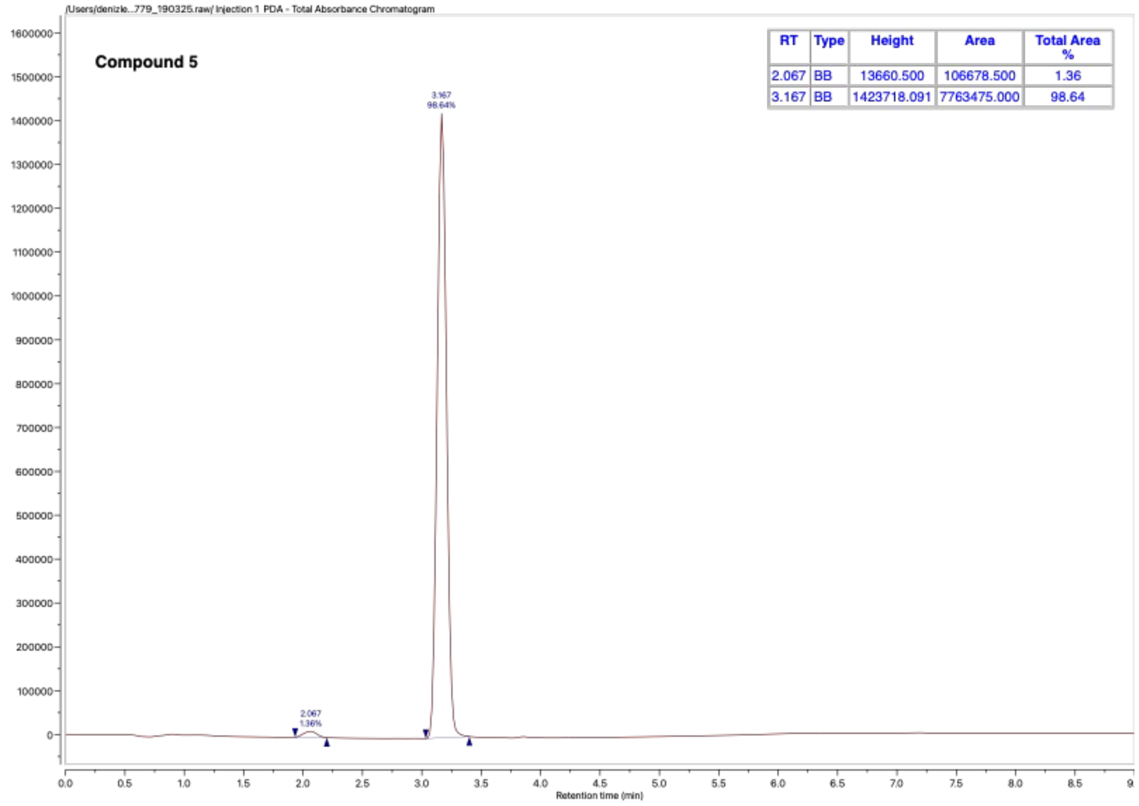

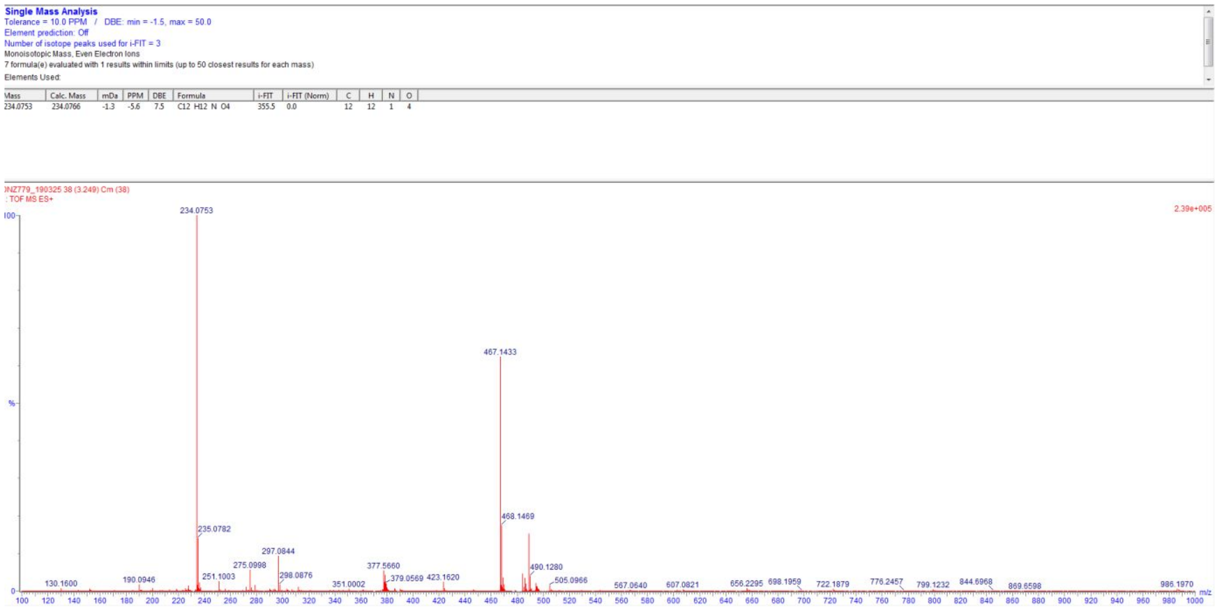

Figure S15. UPLC and HRMS chromatograms of **Compound 6**

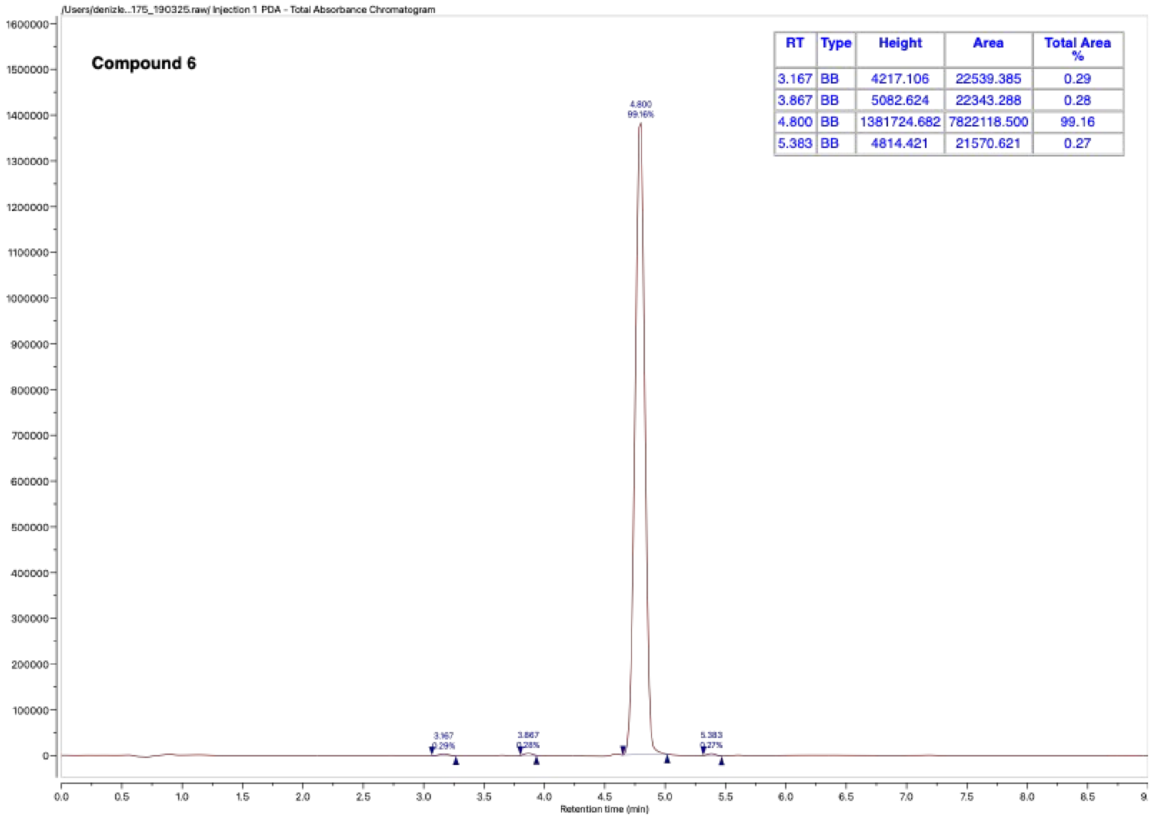

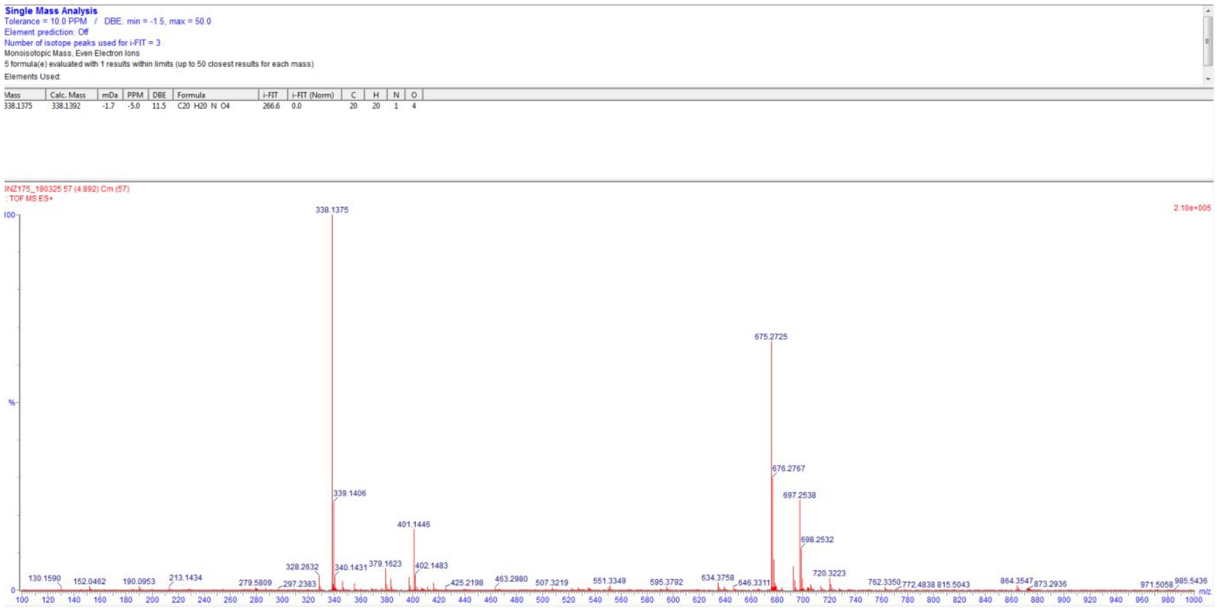

Figure S16. UPLC and HRMS chromatograms of **Compound 7**

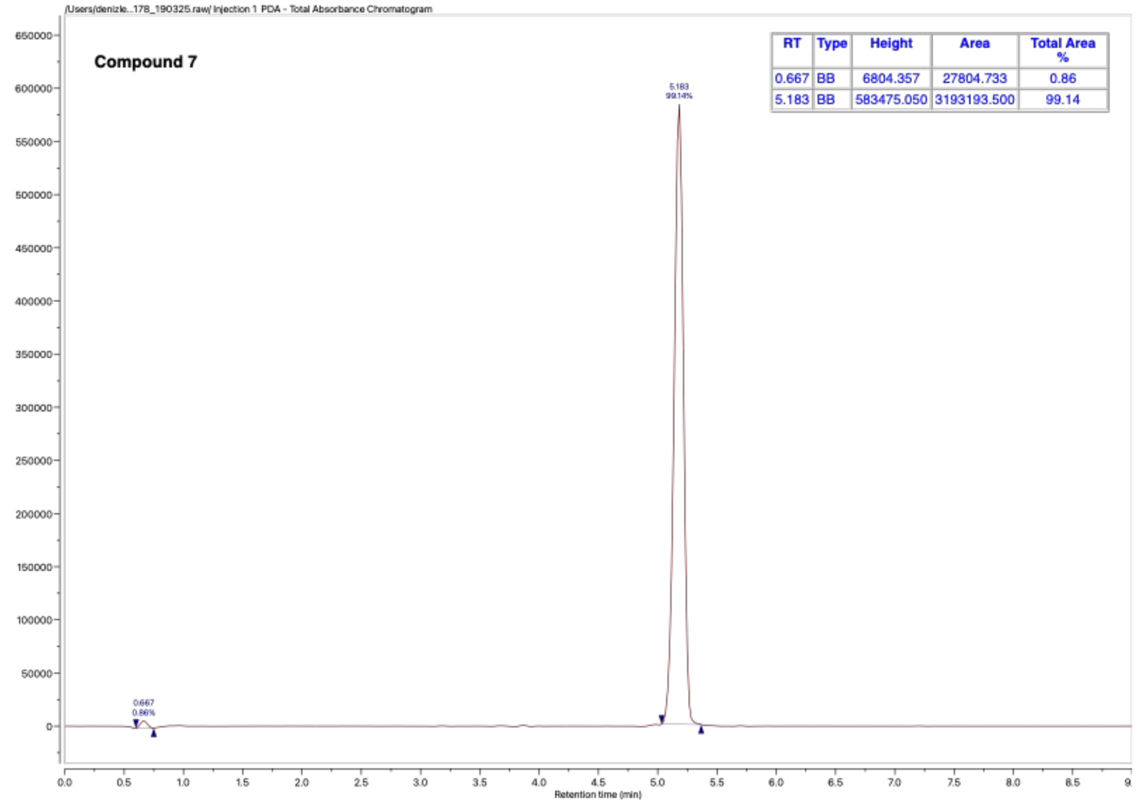

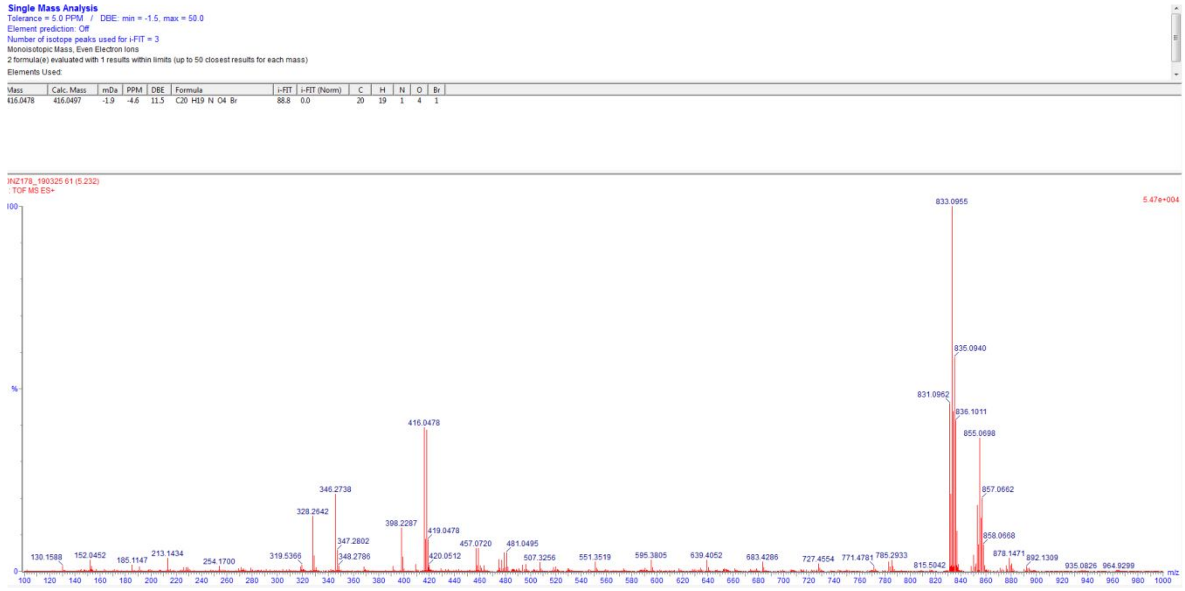

Figure S17. UPLC and HRMS chromatograms of **Compound 8**

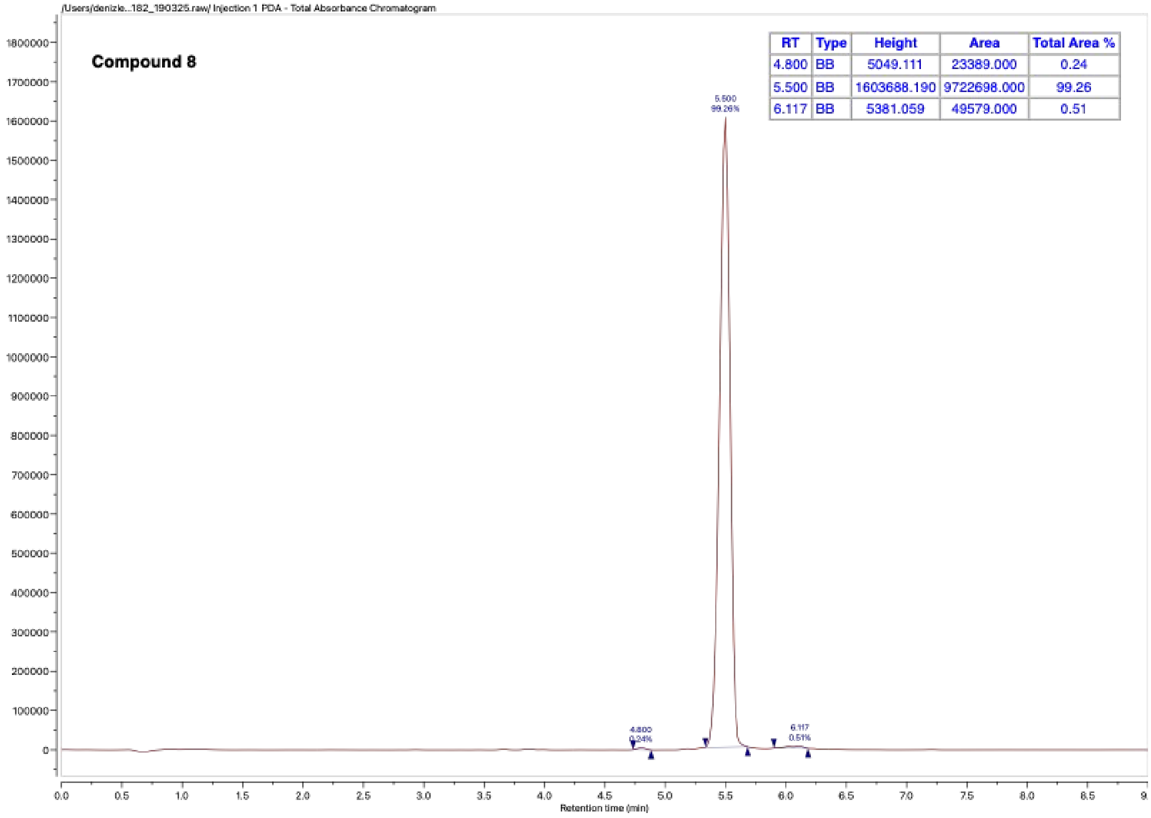

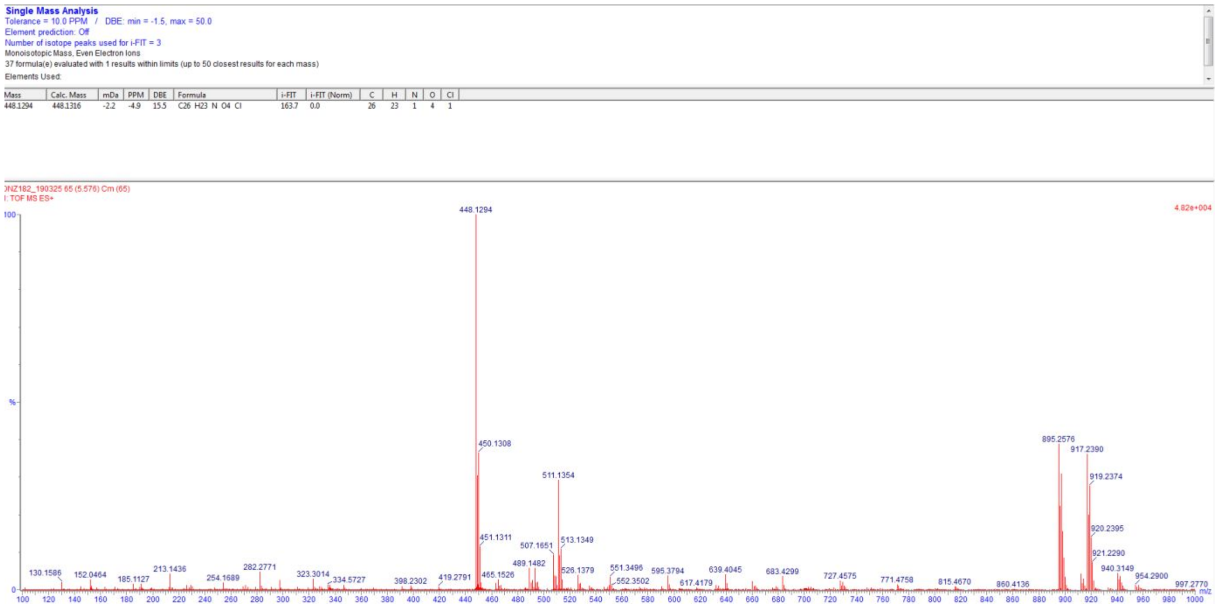

Figure S18. UPLC and HRMS chromatograms of **Compound 9**

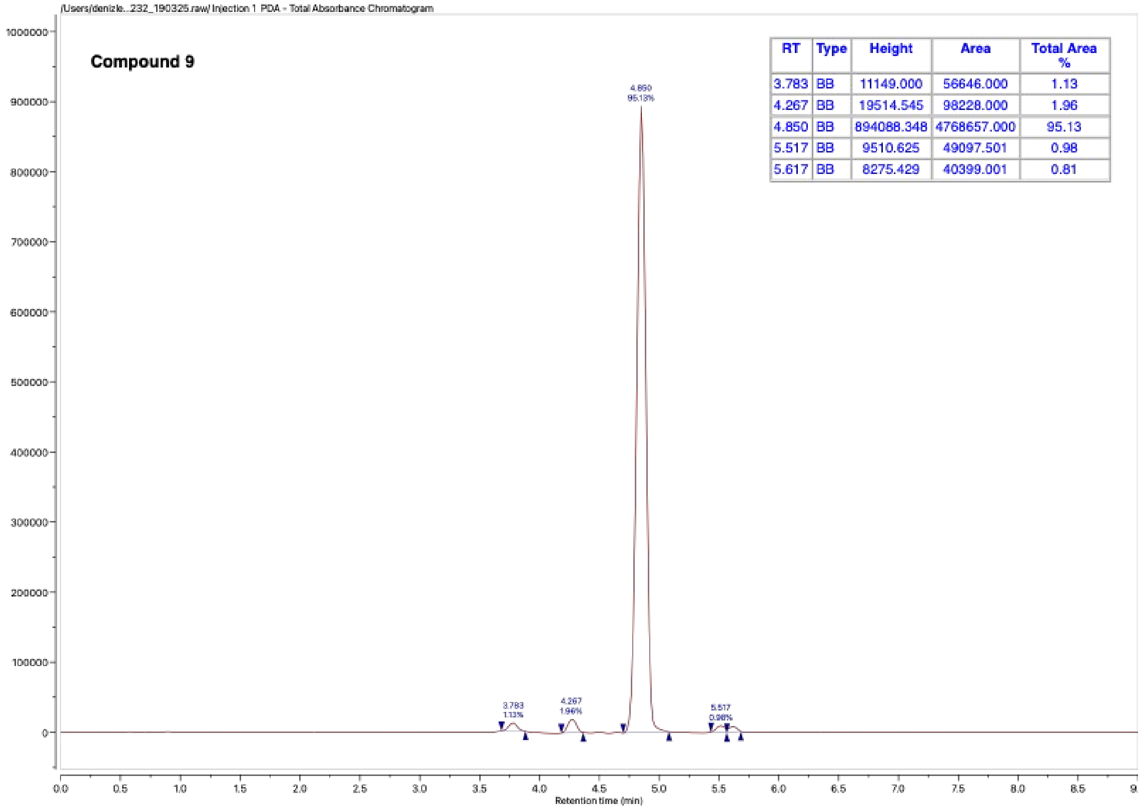

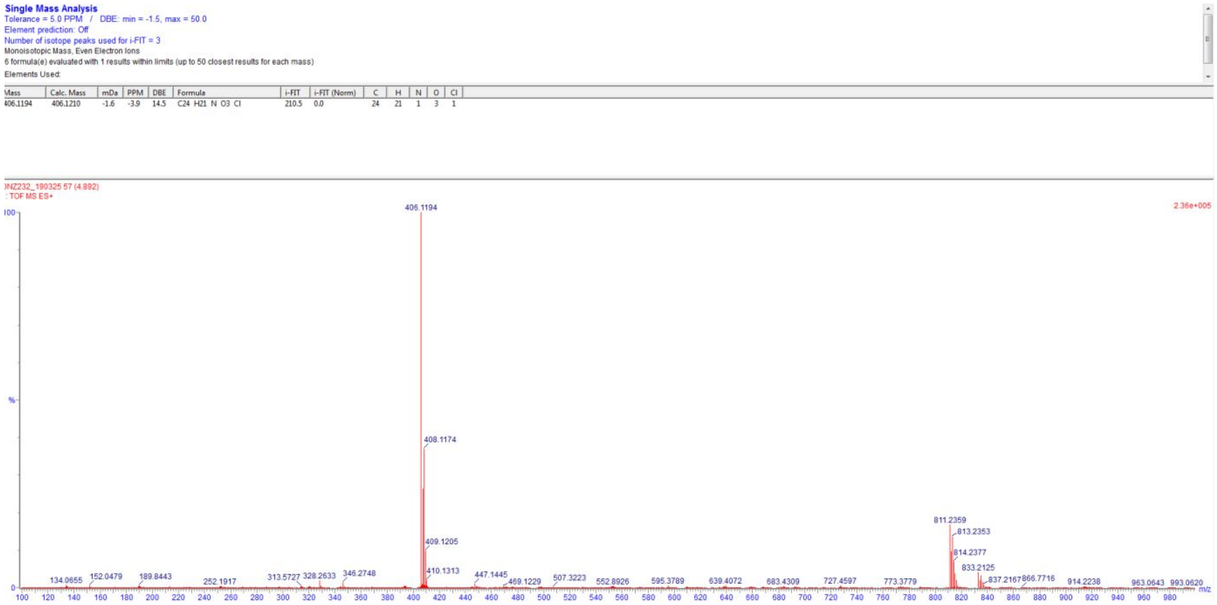

Figure S19. UPLC and HRMS analysis of **C160**

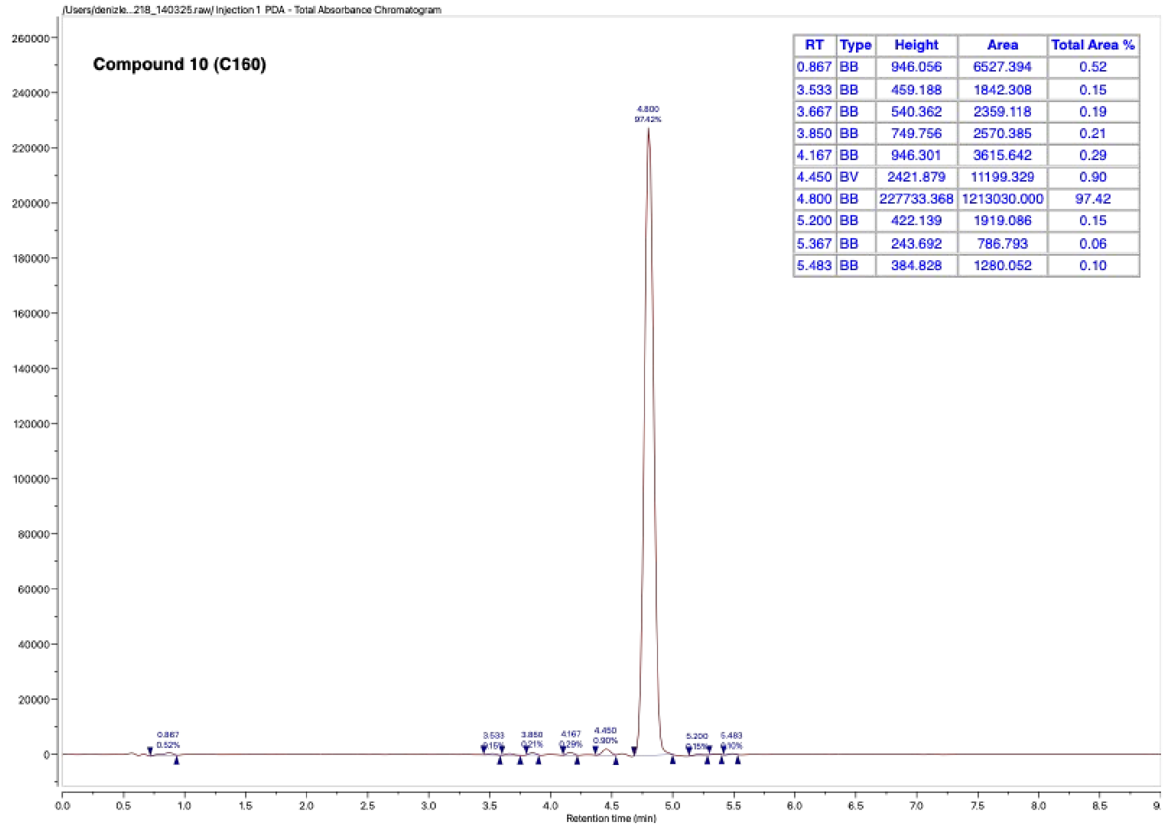

RT	Type	Height	Area	Total Area %
0.867	BB	946.056	6527.394	0.52
3.533	BB	459.188	1842.308	0.15
3.667	BB	540.362	2359.118	0.19
3.850	BB	749.756	2570.385	0.21
4.167	BB	946.301	3615.642	0.29
4.450	BV	2421.879	11199.329	0.90
4.800	BB	227733.368	1213030.000	97.42
5.200	BB	422.139	1919.086	0.15
5.367	BB	243.692	786.793	0.06
5.483	BB	384.828	1280.052	0.10

Single Mass Analysis
Tolerance = 5.0 PPM / DBE: min = -1.5, max = 50.0
Element prediction: Off
Number of isotope peaks used for i-FIT = 3
Monoisotopic Mass, Even Electron Ions
62 formula(e) evaluated with 1 results within limits (up to 50 closest results for each mass)
Elements Used:

Mass	Calc. Mass	mDa	PPM	DBE	Formula	i-FIT	i-FIT (Norm)	C	H	N	O	S	Cl
632.1995	632.1986	0.9	1.4	18.5	C34 H35 N3 O5 S Cl	113.3	0.0	34	35	3	5	1	1

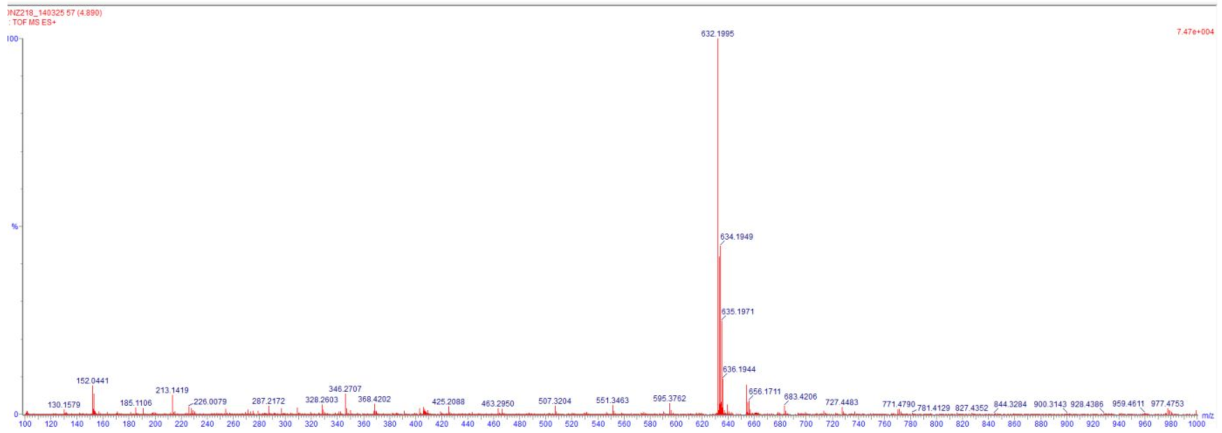

Figure S20. UPLC and HRMS analysis of **C161**

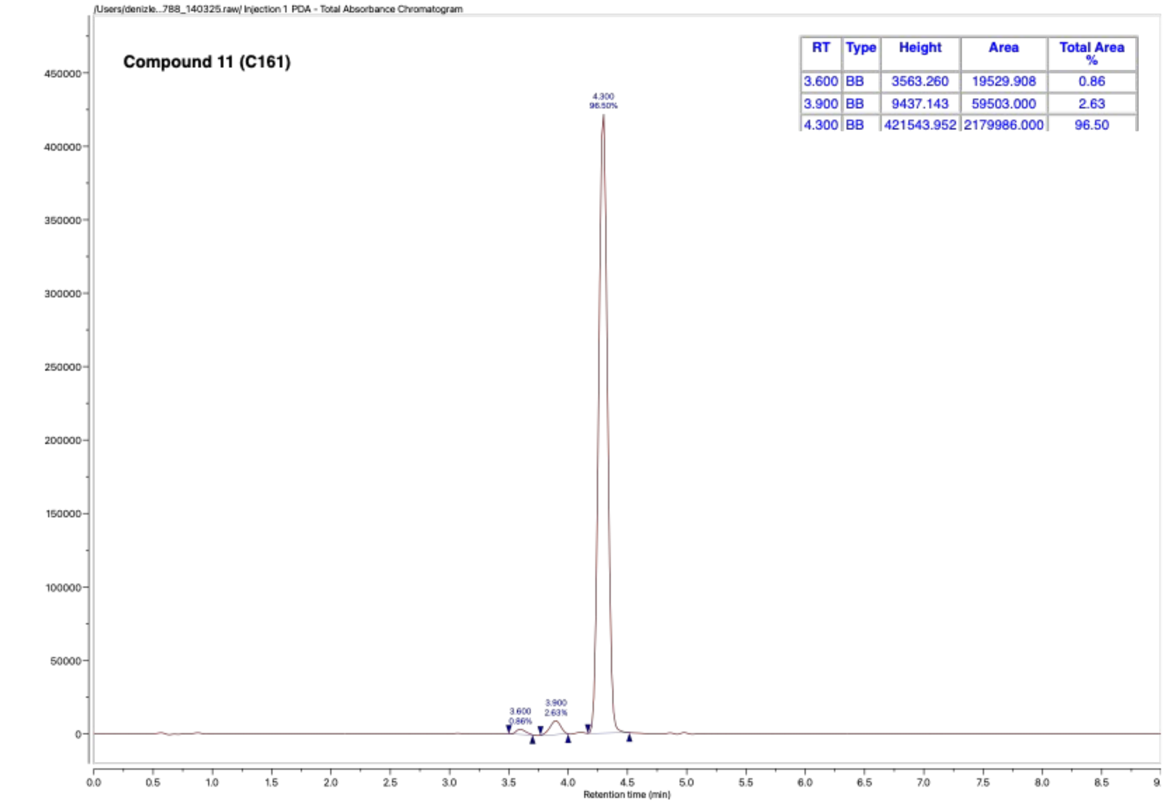

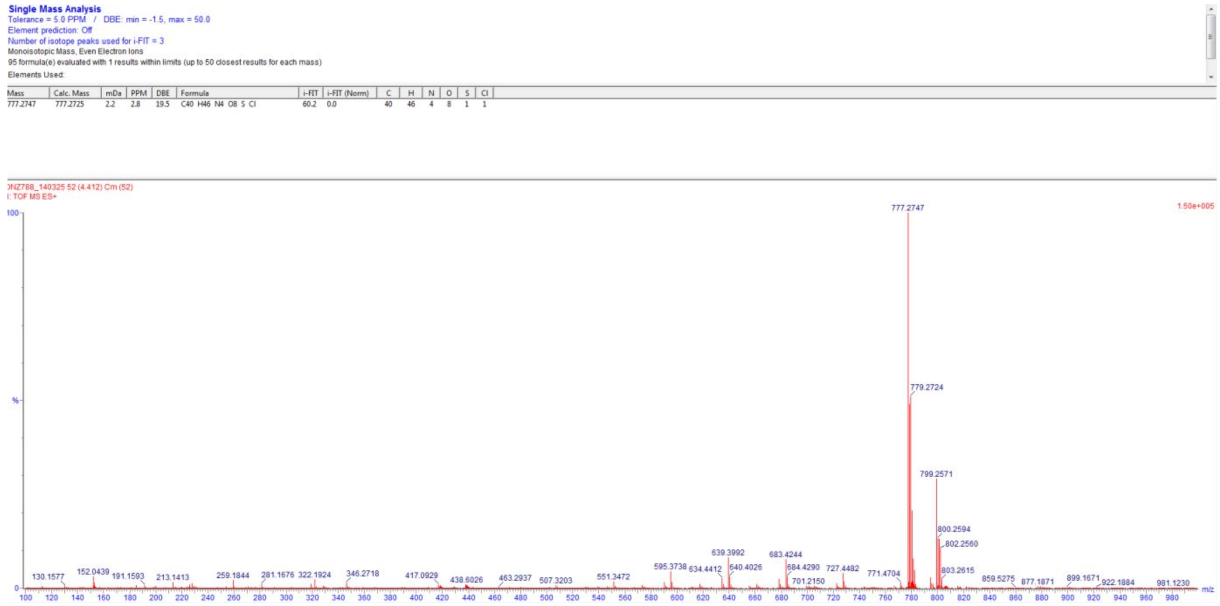

Figure S21. Autodock Vina Binding Affinities for the Compounds Ruxolitinib and **EB38**

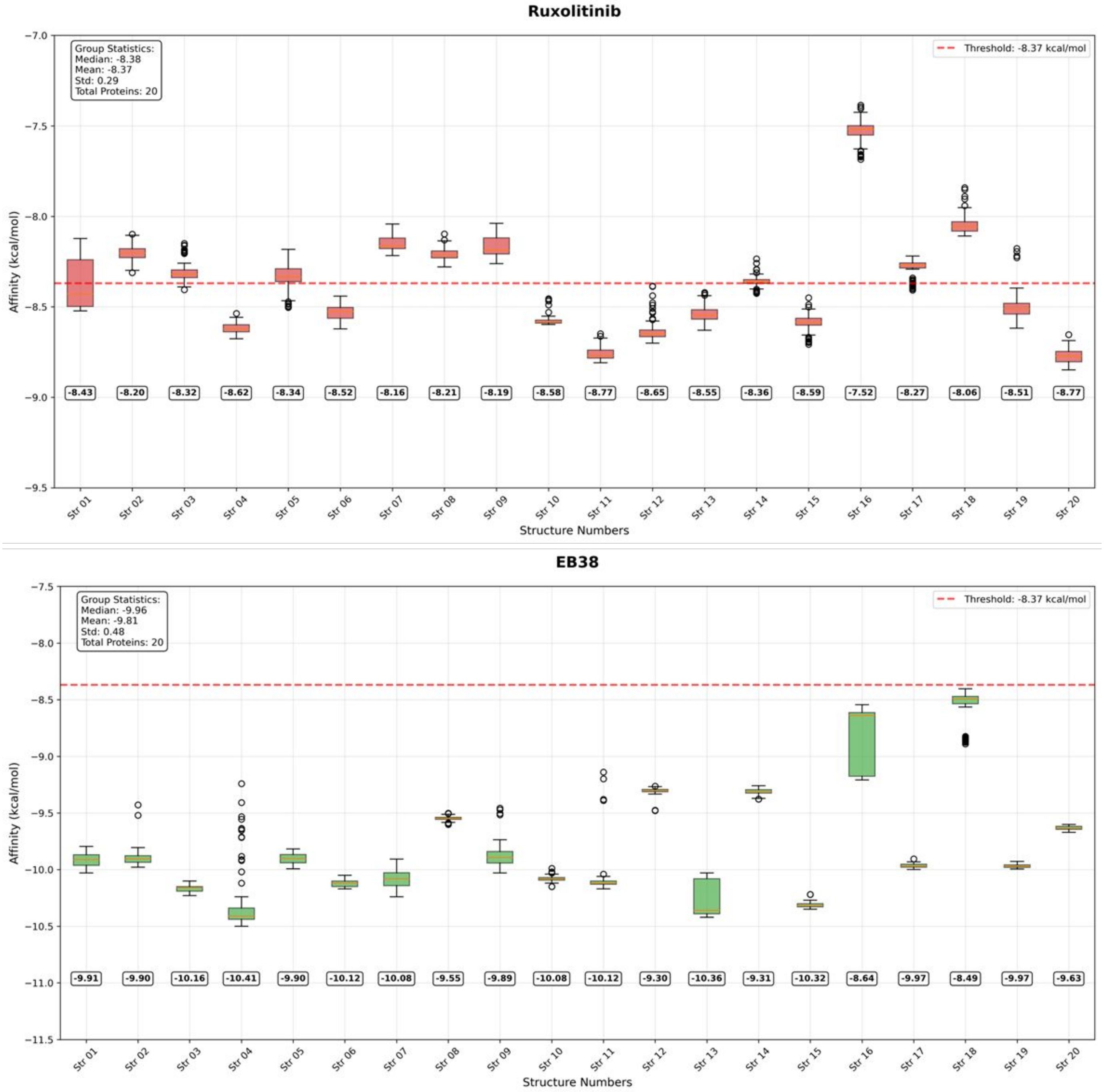

Figure S22. Autodock Vina Binding Affinities for **C160** and **C161**

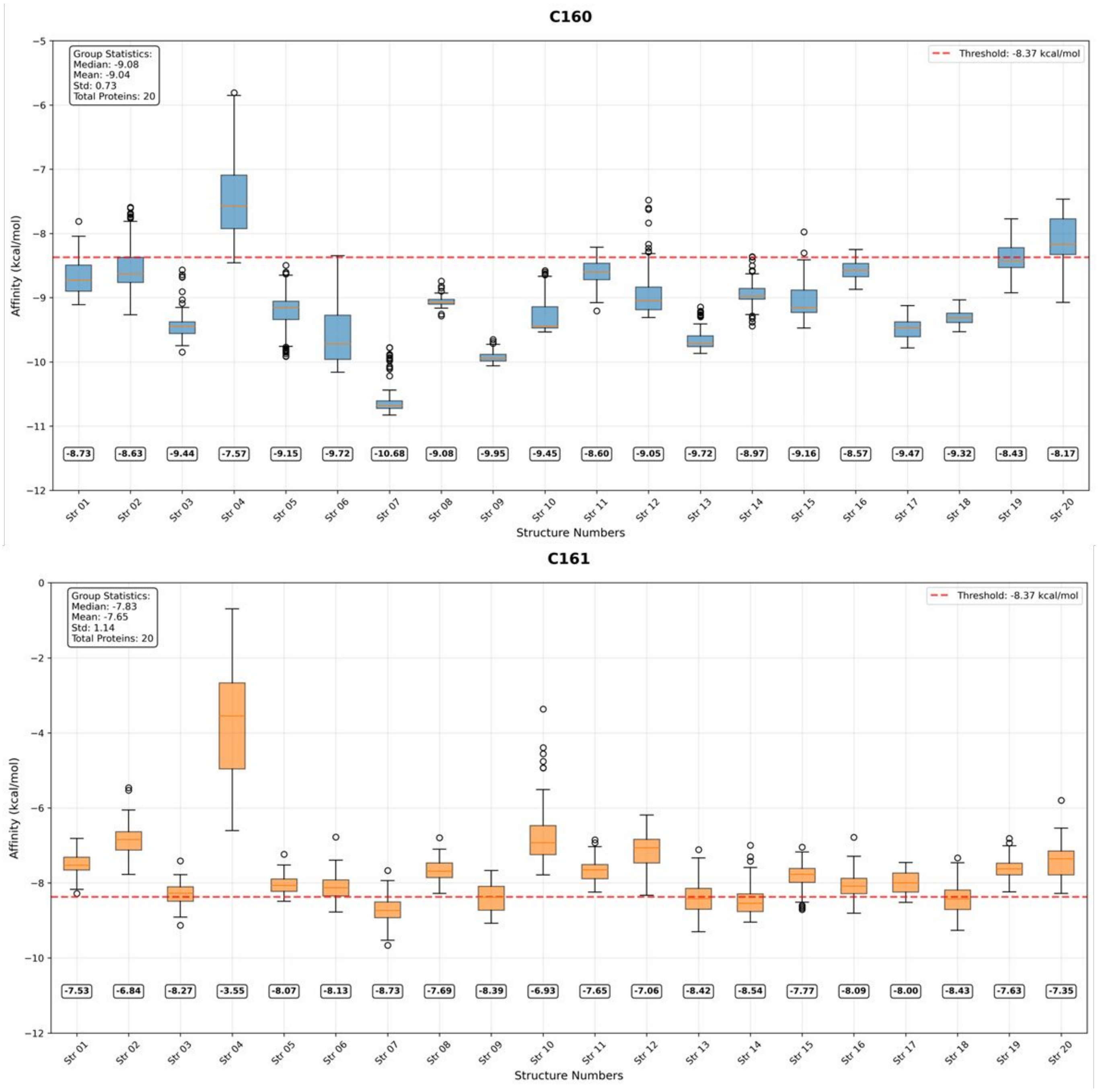

Table S1. Stability of test and control compounds in human and mouse plasma *

Compound ID	Species	Remaining Percentages (%)					$t_{1/2}$ (min)
		0 min	15 min	30 min	60 min	120 min	
Propantheline	Human	100,00	71,29	47,89	20,87	4,08	26,34
	Mouse	100,00	65,48	46,72	20,25	5,14	26,27
C160	Human	100,00	95,45	96,44	91,75	89,02	> 372.67
	Mouse	100,00	3,50	2,58	3,02	4,09	3,10
C161	Human	100,00	98,99	91,50	80,00	67,13	198,11
	Mouse	100,00	0,01	0,02	0,01	0,02	1,11

***Plasma stability test was carried at Pharmaron Inc. (Beijing, China).**

Table S2. pKA Values of the Test and Control Compounds

Ligand	Index	Smiles	acid_or_base	pKa
C160	000	ClC(C=C1)=CC=C1C(C(COC(CCCCC2SCC(N3)C2NC3=O)=O)=NO4)=C4C5=CC=C(OCC6=C(C)C=CC=C6)C=C5	Acid	14.0
C160	001	ClC(C=C1)=CC=C1C(C(COC(CCCCC2SCC(N3)C2NC3=O)=O)=NO4)=C4C5=CC=C(OCC6=C(C)C=CC=C6)C=C5	Acid	14.0
C161	000	ClC(C=C1)=CC=C1C(C(COC(COCCOCCNC(CCCCC2SCC(N3)C2NC3=O)=O)=O)=NO4)=C4C5=CC=C(OCC6=C(C)C=CC=C6)C=C5	Acid	13.8
C161	001	ClC(C=C1)=CC=C1C(C(COC(COCCOCCNC(CCCCC2SCC(N3)C2NC3=O)=O)=O)=NO4)=C4C5=CC=C(OCC6=C(C)C=CC=C6)C=C5	Acid	13.9
C161	002	ClC(C=C1)=CC=C1C(C(COC(COCCOCCNC(CCCCC2SCC(N3)C2NC3=O)=O)=O)=NO4)=C4C5=CC=C(OCC6=C(C)C=CC=C6)C=C5	Acid	13.9
EB38		ClC(C=C1)=CC=C1C2=C(C)ON=C2C3=CC=C(OCC4=C(C)C=CC=C4)C=C3	No ionizable groups found	
Ruxolitinib	000	C1CCC(C1)[C@@H](CC#N)N2C=C(C=N2)C3=C4C=CNC4=NC=N3	Acid	11.8
Ruxolitinib	001	C1CCC(C1)[C@@H](CC#N)N2C=C(C=N2)C3=C4C=CNC4=NC=N3	Base	3.2
Ruxolitinib	002	C1CCC(C1)[C@@H](CC#N)N2C=C(C=N2)C3=C4C=CNC4=NC=N3	Base	2.4
Ruxolitinib	003	C1CCC(C1)[C@@H](CC#N)N2C=C(C=N2)C3=C4C=CNC4=NC=N3	Base	3.6
Ruxolitinib	004	C1CCC(C1)[C@@H](CC#N)N2C=C(C=N2)C3=C4C=CNC4=NC=N3	Base	3.1
